# Supplementary material for: Crystalline hydrogen bonding of water molecules confined in a metal-organic framework
Source: Commun Chem. 2022 Apr 8;5:51. doi: 10.1038/s42004-022-00666-8 (PMC9814150; doi:10.1038/s42004-022-00666-8)
Supplement: Supplementary file 1 — Supplementary Information [file 42004_2022_666_MOESM1_ESM.pdf]

Supplementary Information

Crystalline hydrogen bonding of water molecules confined in a metal-organic framework

Jinhee Bae[1], Sun Ho Park[1], Dohyun Moon[2]* & Nak Cheon Jeong[1,3]*

[1]Department of Physics and Chemistry, DGIST, Daegu 42988, Korea
[2]Beamline Department, Pohang Accelerator Laboratory, Pohang 37673, Korea
[3]Center for Emerging Materials Science, DGIST, Daegu 42988, Korea

*To whom correspondence should be addressed. E-mail: nc@dgist.ac.kr

Table of contents

Supplementary Section 1. Raman spectra of various solvents-coordinating HKUST-1 .... S3
Supplementary Section 2. Topological Views of HKUST-1 .... S4
Supplementary Section 3. ^{1}H NMR and PXRD data of as-prepared HKUST-1 samples S5
Supplementary Section 4. Raman spectra of HKUST-1 crystal measured at 220 K S6
Supplementary Section 5. SCXRD data of HKUST-1 crystals collected at 220 K S7
Supplementary Section 6. Raman spectra of an HKUST-1 crystal measured at 298 K S12
Supplementary Section 7. Raman spectra of bulk water and water-coordinating HKUST-1 measured at various temperatures S13
Supplementary Section 8. Raman spectra of isotopic water-coordinating HKUST-1 .... S14
Supplementary Section 9. Raman spectra of EtOH- and MeOH-filling HKUST-1 crystals measured at 298 K S15
Supplementary Section 10. Raman spectra of bulk methanol measured at 20 and –110 °C S18
Supplementary Section 11. In situ Raman spectra of an HKUST-1 crystal measured at room temperature S19
Supplementary Section 12. In situ infra-red spectra of an HKUAT-1 crystal measured at room temperature S20
Supplementary Section 13. Theoretical number of hydrogen bondings around a coordinating H_2O molecule S21
Supplementary Section 14. UV-vis absorption spectra of activated and water-filling HKUST-1s S22
Supplementary Section 15. In situ SCXRD data of HKUST-1 collected at 298 K S23
(1) Activated HKUST-1 (Act-HKUST-1) S24
(2) Moisture-exposed HKUST-1 for 5 min (H_2O-HKUST-1(1st)) S25
(3) Moisture-exposed HKUST-1 for 10 min (H_2O-HKUST-1(2nd)) S26
(4) Moisture-exposed HKUST-1 for 15 min (H_2O-HKUST-1(3rd)) S27
(5) Moisture-exposed HKUST-1 for 20 min (H_2O-HKUST-1(4th)) S28
(6) Moisture-exposed HKUST-1 for 30 min (H_2O-HKUST-1(5th)) S29
(7) Moisture-exposed HKUST-1 for 60 min (H_2O-HKUST-1(6th)) S30
(8) Moisture-exposed HKUST-1 for 120 min (H_2O-HKUST-1(7th)) S31
(9) Moisture-exposed HKUST-1 for 180 min (H_2O-HKUST-1(8th)) S32
(10) Moisture-exposed HKUST-1 for 240 min (H_2O-HKUST-1(9th)) S33
Supplementary Section 16. Summary of the HKUST-1 crystal structure obtained from in situ SCXRD S34
Supplementary Section 17. Changes in lattice parameters of HKUST-1 .... S35
Supplementary Section 18. Water vapor sorption of HKUST-1 .... S38
References S39

Supplementary Section 1. Raman spectra of various solvents-coordinating HKUST-1.

Our previous report demonstrated that the stretching vibration of the Cu−Cu metallic bonding is sensitive to the coordination environment around the Cu^{2+} node. Raman spectra of H_2O-, MeOH-, EtOH- and DMF-coordinated HKUST-1 samples show that the Cu–Cu stretching vibration exhibited at approximately 163-175 cm^{-1}, depending on the coordinating solvent molecule[S1-S2].

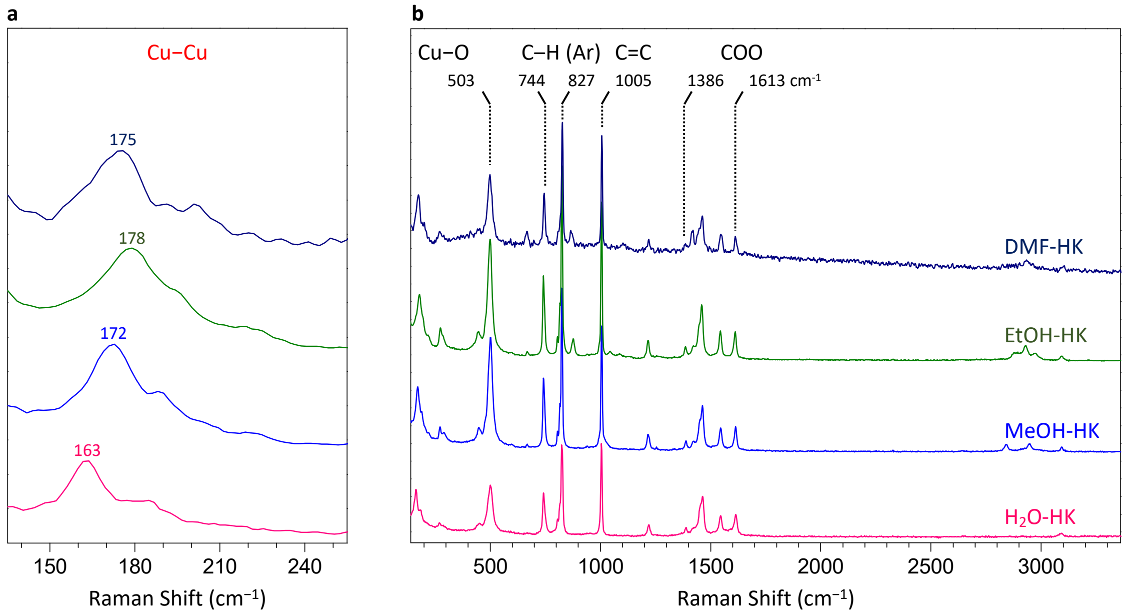

Supplementary Figure 1. (a) Expanded and (b) broad views of Raman spectra of H_2O-, MeOH-, EtOH- and DMF-coordinated HKUST-1 crystals recorded at room temperature.

Supplementary Section 2. Topological Views of HKUST-1.

HKUST-1 consists of two types of large cages with the shape of a truncated cube (**type-I** and **-II** cages) and a type of small cage with the shape of a sodalite-like truncated octahedron (**type-III**). In a topological view, alternating stacking of type-I and -II large cages in the fashion of face-centred cube constructs HKSUT-1, where the type-III small cages are placed at the corner of the cube[S3]. In the other topological view, stacking type-III small cages in the fashion of primitive cube also constructs HKUST-1[S3].

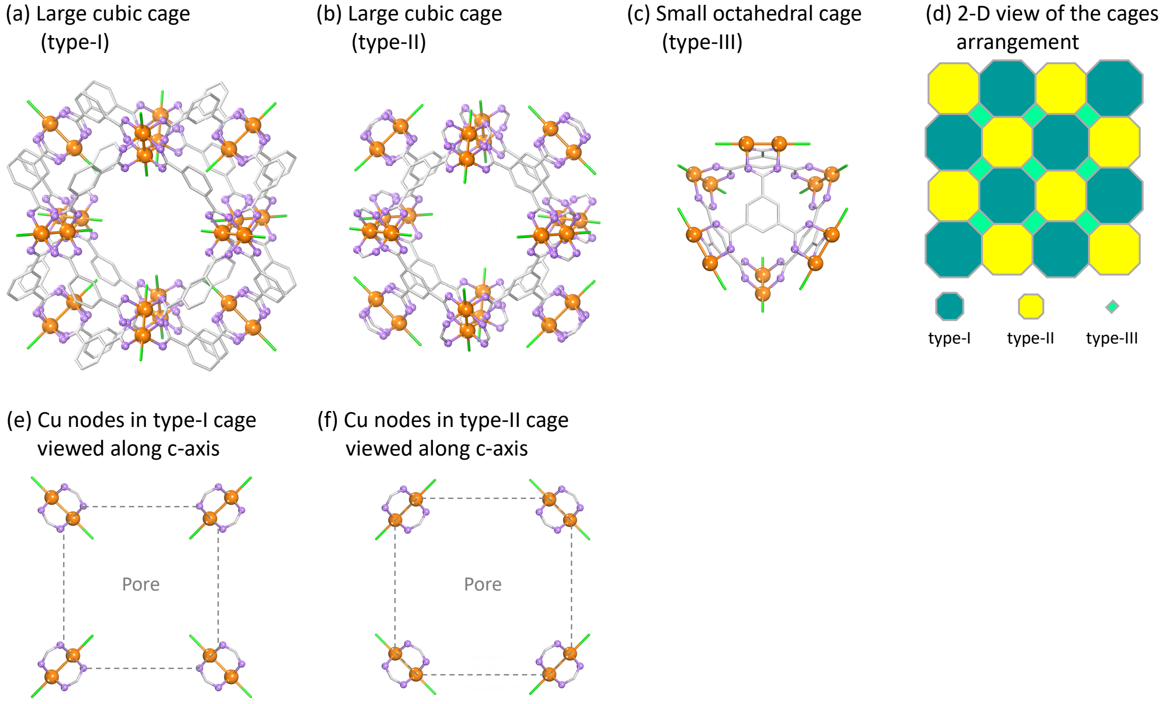

Supplementary Figure 2. (a-c) Illustrations of three types of cages in HKUST-1 and (d) a 2-dimensional (2-D) representation of the topological arrangement of the cages. (e-f) 2-D views of conformational arrangement of Cu nodes in (e) type-I and (f) type-II cages.

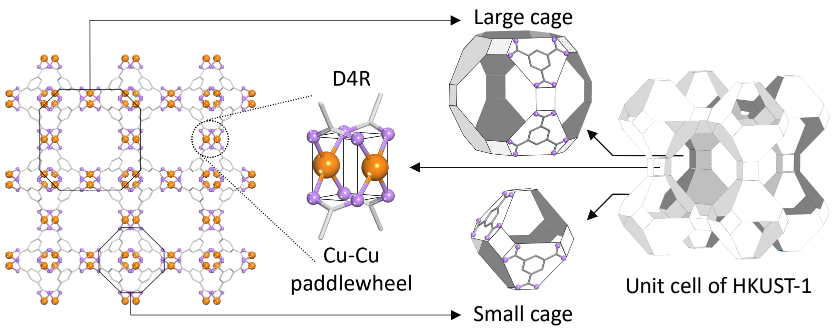

Supplementary Figure 3. [100] View of the crystal structure of HKUST-1 framework (left) and 3-dimensional illustration of the unit cell and building units of HKUST-1 (right). Cu-Cu paddlewheel node in a D4R secondary building unit constitutes the wall of a large cubic cage and a small octahedral cage in HKUST-1. Hydrogen atoms bound to carbon atoms in the benzene moieties are omitted for clarity.

Supplementary Section 3. ^{1}H NMR and PXRD data of as-prepared HKUST-1 samples.

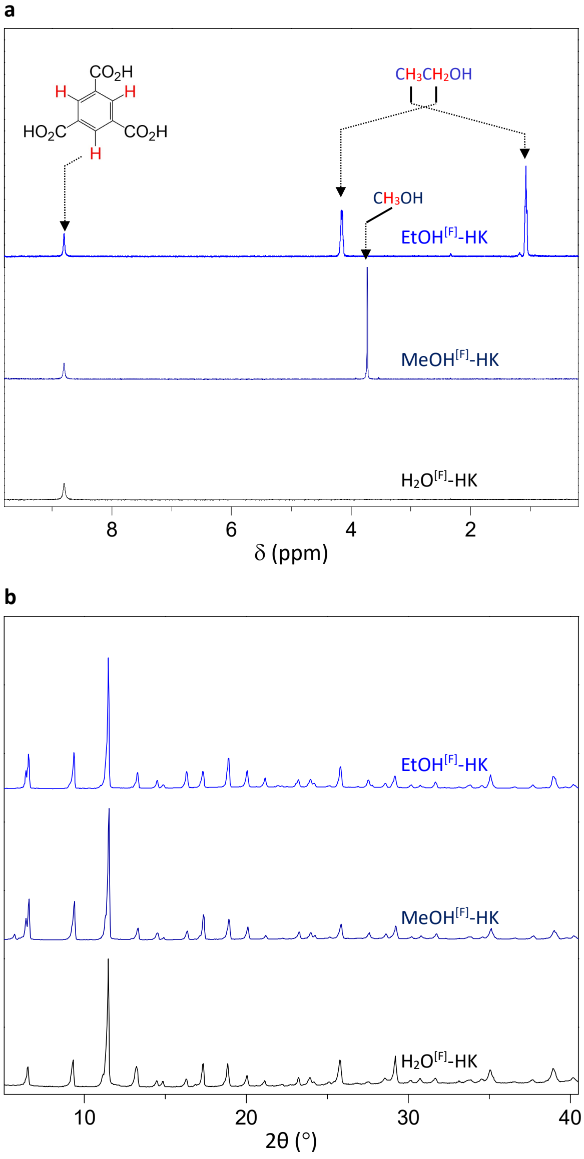

Supplementary Figure 4. (a) ^{1}H nuclear magnetic resonance (NMR) spectra and (b) powder X-ray diffraction (PXRD) patterns of as-prepared H_2O[F]-HKUST-1, MeOH[F]-HKUST-1 and EtOH[F]-HKUST-1 crystal samples as designated.

Supplementary Section 4. Raman spectra of HKUST-1 crystal measured at 220 K.

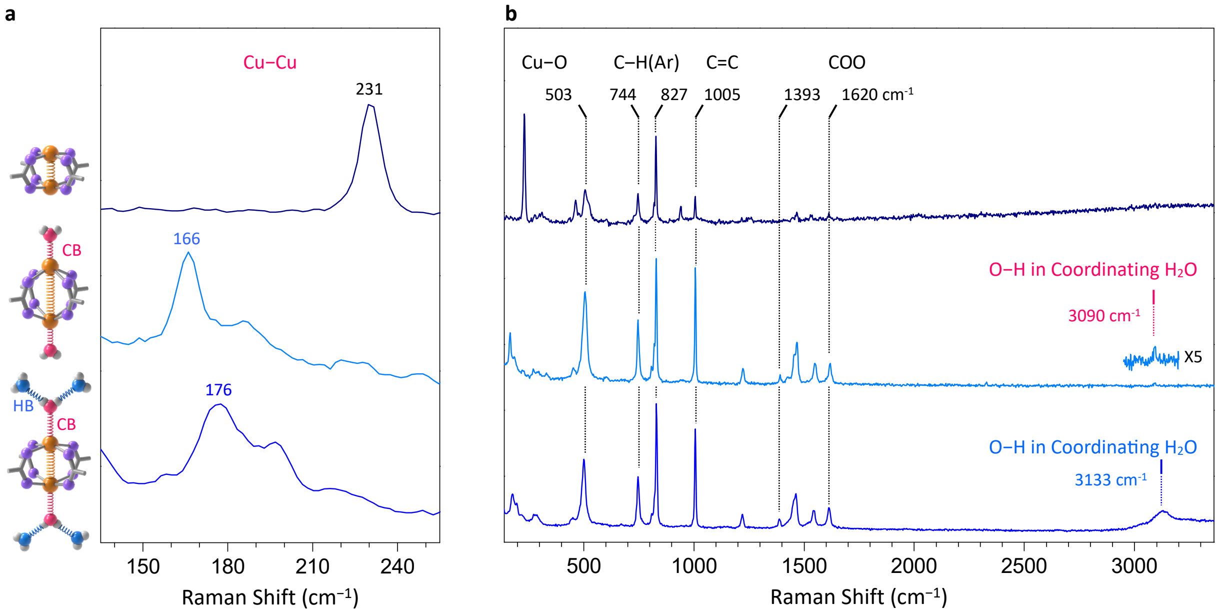

Supplementary Figure 5. (a) Expanded and (b) wide views of Raman spectra of Act-HKUST-1, $H_2O^{[C]}$-HKUST-1 and $H_2O^{[F]}$-HKUST-1 crystals measured at 220 K.

Supplementary Section 5. SCXRD data of HKUST-1 crystals collected at 220 K.

To obtain the single-crystal X-ray diffraction (SCXRD) structures of Act-HKUST-1, $H_2O^{[C]}$-HKUST-1 and $H_2O^{[F]}$-HKUST-1, first, we coated the single crystals with Parabar 10312 oil (Hampton Research Inc.). After a single crystal is placed in the gripper, the single-crystal X-ray diffraction data of the crystals were collected at 220 K by using silicon (111) double crystal monochromator, synchrotron radiation with the wavelength (λ) of 0.61000 Å and Rayonix MX225HS detectors, which are installed at BL2D-SMC in the Pohang Accelerator Laboratory (PAL). Using PAL BL2D-SMDC software[S4], the diffraction data were collected every 0.6 seconds for a frame at the detector distance of approximately 66 mm with the omega scan ($\Delta\omega$) of 3°. Also, a software of HKL3000sm (Ver. 720)[S5] was used for the cell refinement, reduction, and absorption correction. Then, the crystal structures of the series HKUST-1 crystals were solved by the intrinsic phasing method with the software of the SHELXT-2018[S6] and subsequently refined by full-matrix least-squares calculations with a software of the SHELXL-2018[S7]. All atoms except hydrogen were refined, considering the atomic anisotropy. Hydrogen atoms bound to carbon atoms in the benzene moieties were considered to be placed at geometrically ideal positions and constrained to ride on their parent atoms with the C-H bond length of 0.94 Å and the Uiso(H) value of 1.2 U_{eq} for the parent atoms. As for $H_2O^{[C]}$-HKUST-1 and $H_2O^{[F]}$-HKUST-1 samples, the hydrogen atoms in the coordinated water molecules were found in difference-Fourier maps and restrained by using DFIX and DANG commands during the least-squares refinement with U_{iso}(H) values of 1.2U_{eq} of the oxygen atom. Crystal data, data collection and structure refinement details are summarized in Supplementary Table 1−3.

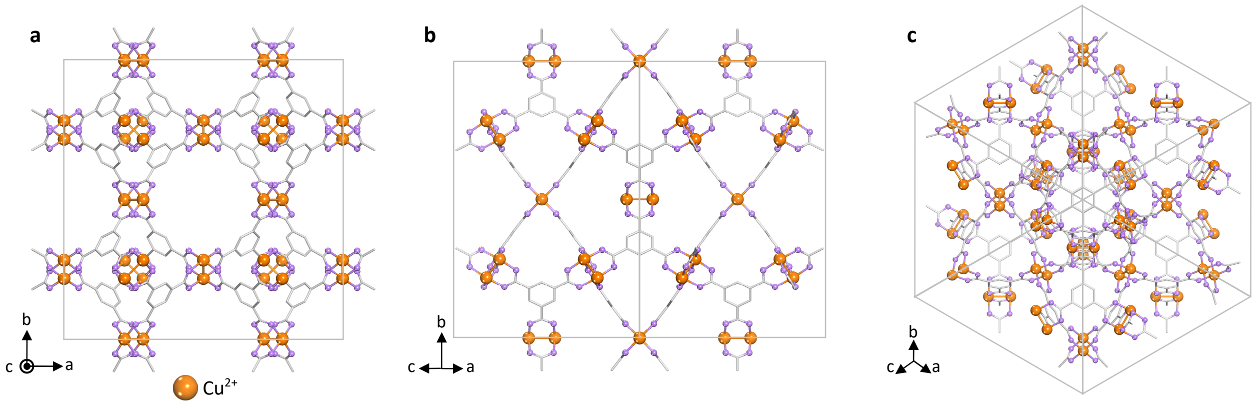

Supplementary Figure 6. (a) [001], (b) [101] and (c) [111] views of the SCXRD structure of an Act-HKUST-1 single-crystal recorded at 220 K. Hydrogen atoms bound to carbon atoms in benzene moieties are omitted for the sake of clarity.

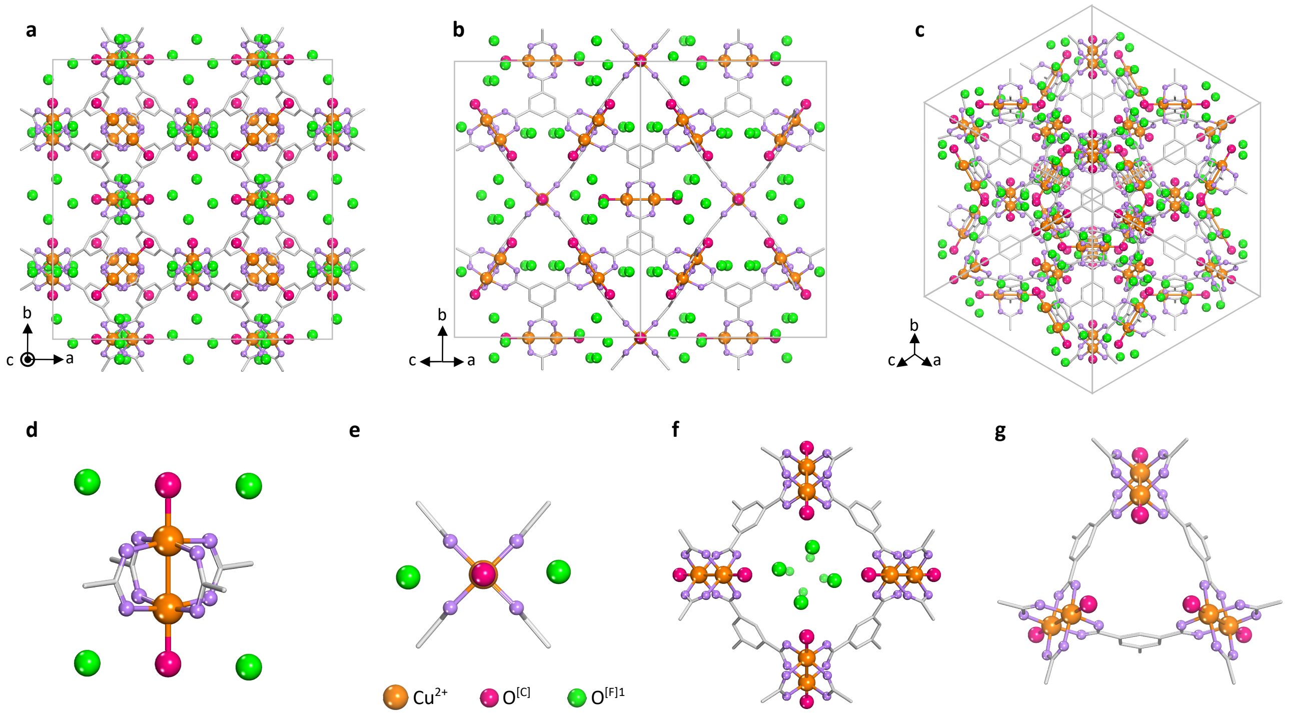

Supplementary Figure 7. (a) [001], (b) [101] and (c) [111] views of the SCXRD structure of an $H_2O^{[C]}$-HKUST-1 single-crystal taken at 220 K. (d-g) illustrations of the spatial configuration of pore-filling H_2O molecules (d-e) around Cu^{2+} node viewed (d) three-dimensionally and (e) two-dimensionally along the axial direction of Cu^{2+} centre and (f-g) around (f) type-I and (g) type-III cages. Red and green spheres indicate oxygen atoms in $H_2O^{[C]}$ and $H_2O^{[F]1}$ molecules, respectively. Hydrogen atoms bound to carbon atoms in benzene moieties and water molecules are omitted for clarity.

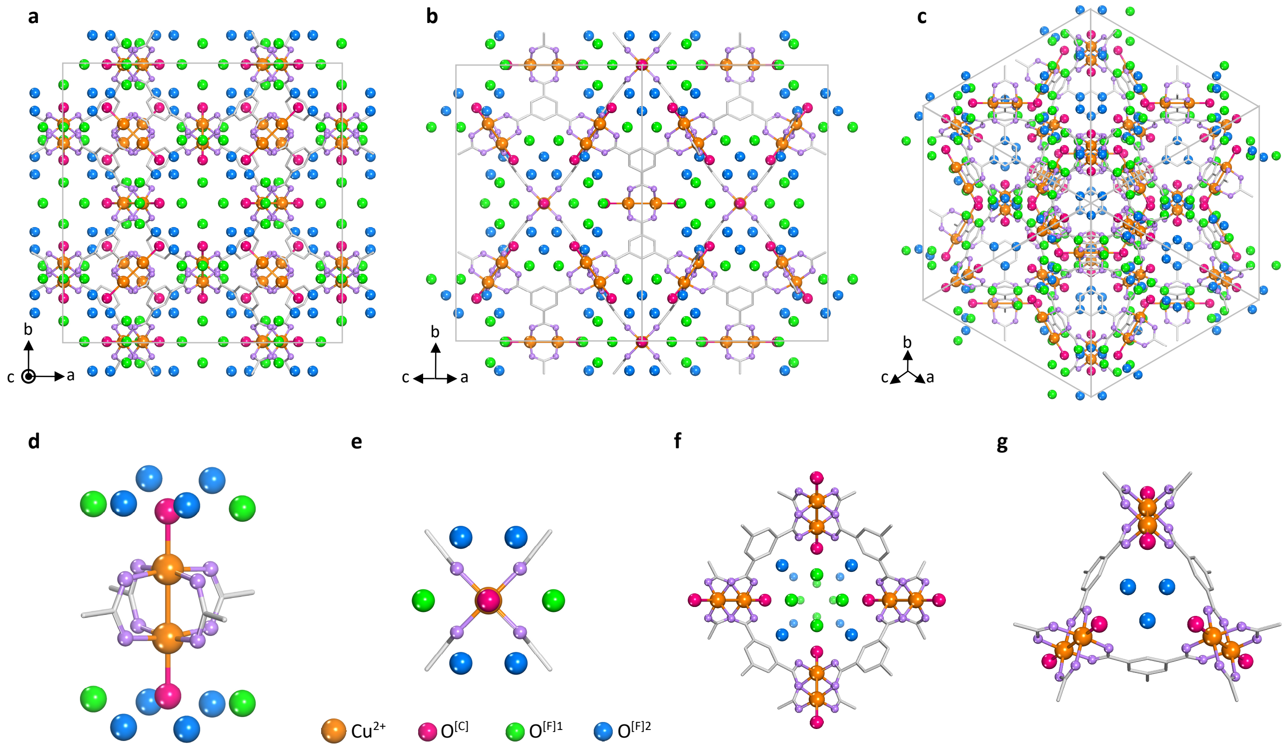

Supplementary Figure 8. (a) [001], (b) [101] and (c) [111] views of the SCXRD structure of an $H_2O^{[F]}$-HKUST-1 single-crystal measured at 220 K. (d-g) illustrations of the spatial configuration of pore-filling H_2O molecules (d-e) around Cu^{2+} node viewed (d) three-dimensionally and (e) two-dimensionally along the axial direction of Cu^{2+} centre and (f-g) around (f) type-I and (g) type-III cages. Red, green and blue spheres indicate oxygen atoms in $H_2O^{[C]}$, $H_2O^{[F]1}$ and $H_2O^{[F]2}$ molecules, respectively. Hydrogen atoms bound to carbon atoms in benzene moieties and water molecules are omitted for the sake of clarity.

Supplementary Table 1. Crystal data and structure refinement of Act-HKUST-1, $H_2O^{[C]}$-HKUST-1 and $H_2O^{[F]}$-HKUST-1 crystals measured at 220 K.

**Identification code**	**Act-HKUST-1**	**$H_2O^{[C]}$-HKUST-1**	**$H_2O^{[F]}$-HKUST-1**
Empirical formula	$C_{18} H_6 Cu_3 O_{12}$	$C_{18} H_{16.8} Cu_3 O_{17.4}$	$C_{18} H_{24} Cu_3 O_{21}$
Formula weight	604.85	702.13	766.99
Temperature	220(2) K	220(2) K	220(2) K
Wavelength	0.610 Å	0.610 Å	0.610 Å
Crystal system	Cubic	Cubic	Cubic
Space group	Fm-3m	Fm-3m	Fm-3m
Unit cell dimensions	a = 26.267(3) Å α = 90°<br>b = 26.267(3) Å β = 90°<br>c = 26.267(3) Å γ = 90°	a = 26.210(3) Å α = 90°<br>b = 26.210(3) Å β = 90°<br>c = 26.210(3) Å γ = 90°	a = 26.275(3) Å α = 90°<br>b = 26.275(3) Å β = 90°<br>c = 26.275(3) Å γ = 90°
Cu−Cu distance	2.485	2.624	2.613
Volume	18123(6) $Å^3$	18006(6) $Å^3$	18139(6) $Å^3$
Z	16	16	16
Density (calculated)	0.887 Mg/m^3	1.036 Mg/m^3	1.123 Mg/m^3
Absorption coefficient	0.938 mm^{-1}	0.956 mm^{-1}	0.956 mm^{-1}
F(000)	4752	5616	6192
Crystal size	0.125 × 0.117 × 0.085 mm^3	0.125 × 0.115 × 0.103 mm^3	0.225 × 0.215 × 0.205 mm^3
Theta range for data collection	1.331 to 24.993°	2.212 to 24.970°	2.206 to 24.985°
Index ranges	$-36 \leq h \leq 36$<br>$-36 \leq k \leq 36$<br>$-36 \leq l \leq 36$	$-36 \leq h \leq 36$<br>$-36 \leq k \leq 36$<br>$-36 \leq l \leq 36$	$-36 \leq h \leq 36$<br>$-36 \leq k \leq 36$<br>$-36 \leq l \leq 36$
Reflections collected	46582	43354	45312
Independent reflections	1320 [R(int) = 0.0997]	1296 [R(int) = 0.1422]	1315 [R(int) = 0.0917]
Completeness to theta = 21.469°	99.9%	98.3%	99.3%
Absorption correction	Empirical	Empirical	Empirical
Max. and min. transmission	1.000 and 0.956	1.000 and 0.834	1.000 and 0.868
Refinement method	Full-matrix least-squares on F^2	Full-matrix least-squares on F^2	Full-matrix least-squares on F^2
Data / restraints / parameters	1320 / 0 / 33	1296 / 18 / 53	1315 / 9 / 59
Goodness-of-fit on F^2	1.073	1.479	1.158
Final R indices [I>2sigma(I)]	R1 = 0.0273, wR2 = 0.0730	R1 = 0.1220, wR2 = 0.3286	R1 = 0.0511, wR2 = 0.1528
R indices (all data)	R1 = 0.0275, wR2 = 0.0731	R1 = 0.1340, wR2 = 0.3532	R1 = 0.0580, wR2 = 0.1621
Extinction coefficient	0.000191(18)	n/a	n/a
Largest diff. peak and hole	0.450 and -0.509 e.$Å^{-3}$	1.456 and -1.162 e.$Å^{-3}$	0.638 and -0.362 e.$Å^{-3}$

Supplementary Table 2. Summary for Cu–Cu and Cu–O[C] length (Å), O_{BTC}–Cu–O_{BTC} angle (°), and H_2O occupancies (mol%) of the Act-HKUST-1, H_2O[C]-HKUST-1, and H_2O[F]-HKUST-1 samples collected from the SCXRD data at 220 K.

Samples	Length (Å)		Angle (°)	Occupancy of water molecules (mol%)		
	Cu–Cu	Cu–O[C]a	∠O_{BTC}–Cu–O_{BTC}	H_2O[C]b	H_2O[F]1c	H_2O[F]2c
Act-HKUST-1	2.485	–	172.86	0	0	0
H_2O[C]-HKUST-1	2.624	2.150	168.10	100	40	0
H_2O[F]-HKUST-1	2.613	2.199	169.13	100	50	50

aO[C] is oxygen in Cu^{2+}-coordinating H_2O.
bH_2O[C] is Cu^{2+}-coordinating H_2O.
cH_2O[F]1 and H_2O[F]2 are type-1 and -2 pore-filling H_2O molecules, respectively.

Supplementary Table 3. Cu–Cu and Cu–O[C] lengths (Å), Raman vibrational frequencies of Cu–Cu bonding ($\nu_{Cu\text{-}Cu}$, cm^{-1}), and O–O distances (Å) in Act-HKUST-1, H_2O[C]-HKUST-1, and H_2O[F]-HKUST-1 samples measured at 220 K.

Identification code	Act-HKUST-1	H_2O[C]-HKUST-1	H_2O[F]-HKUST-1
Cu–Cu	2.485	2.624	2.613
$\nu_{Cu\text{-}Cu}$ (cm^{-1})	231	166	176
Cu–O[C]a	–	2.150	2.199
O[C]–O[F]1b		3.118	2.825
O[C]–O[F]2b			3.065
O[F]1–O[F]1		2.721	2.772
O[F]1–O[F]2			3.218
O[F]2–O[F]2			2.389

aO[C] is oxygen in Cu^{2+}-coordinating H_2O.
bO[F]1 and O[F]2 are oxygen atoms in type-1 and -2 pore-filling H_2O.

Supplementary Section 6. Raman spectra of an HKUST-1 crystal measured at 298 K.

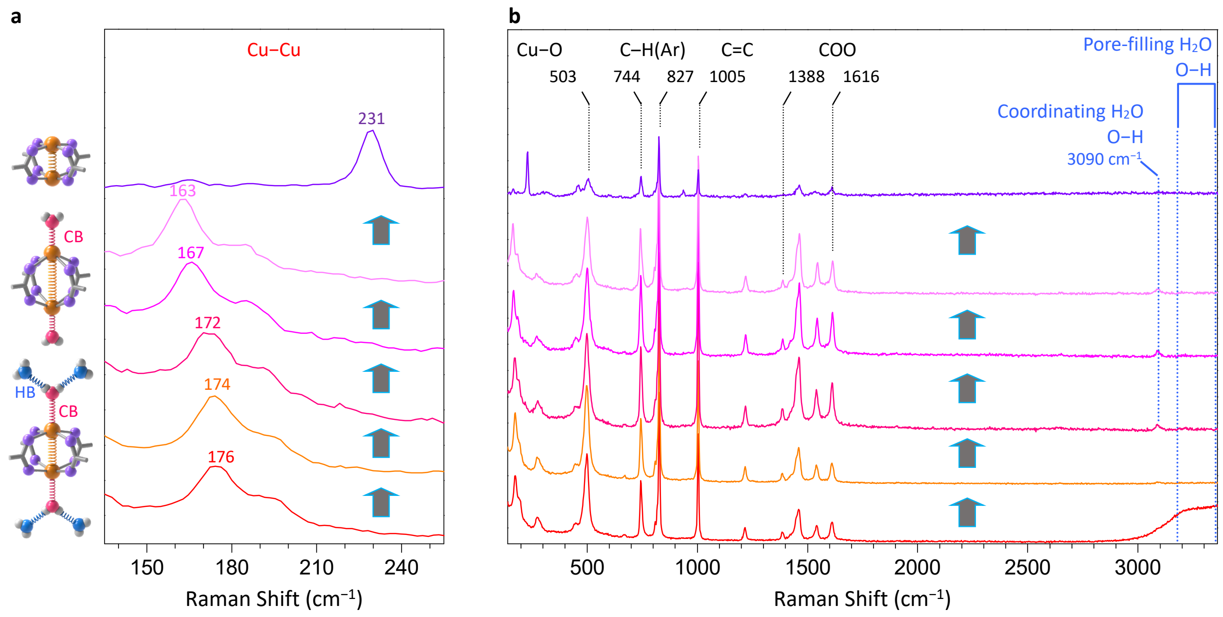

Supplementary Figure 9. (a) Expanded and (b) wide views of successive Raman spectra of an $H_2O^{[F]}$-HKUST-1 crystal sample recorded at 298 K, simultaneously removing pore-filling and coordinating H_2O molecules from the crystals under vacuum conditions.

Supplementary Section 7. Raman spectra of bulk water and water-coordinating HKUST-1 measured at various temperatures.

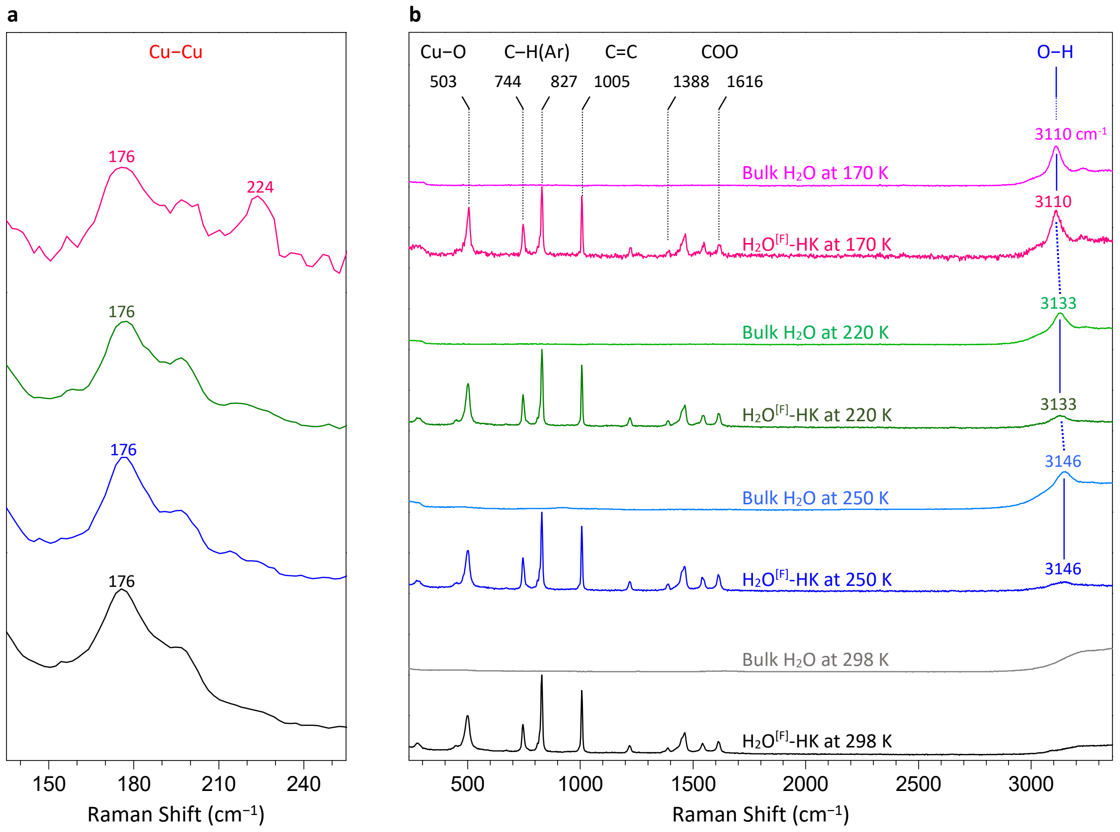

Supplementary Figure 10. (a) Expanded and (b) wide views of Raman spectra of bulk H_2O and an $H_2O^{[F]}$-HKUST-1 recorded at the temperatures of 298, 250, 220 and 170 K as indicated.

Supplementary Section 8. Raman spectra of isotopic water-coordinating HKUST-1.

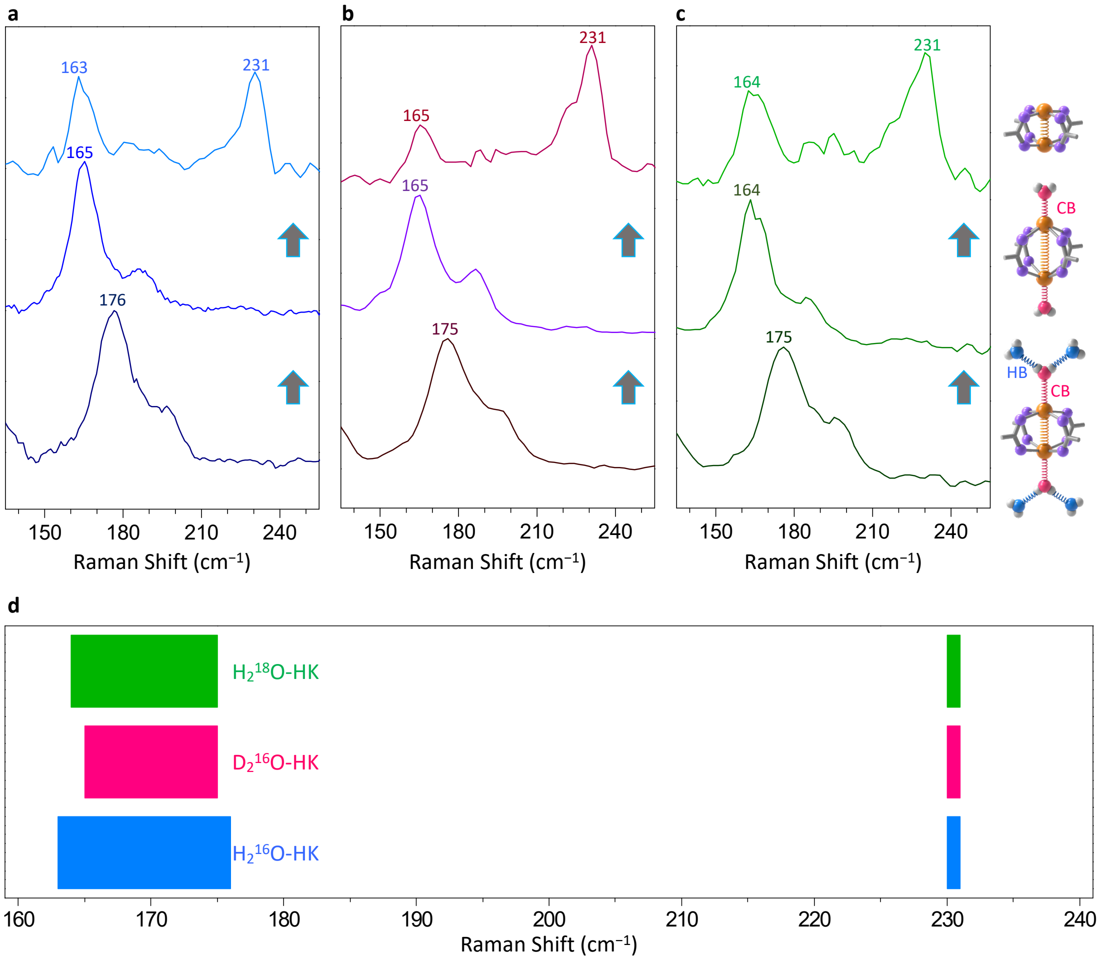

Supplementary Figure 11. (a-c) Expanded views of Raman spectra of (a) $H_2O^{[F]}$-HKUST-1, (b) $D_2O^{[F]}$-HKUST-1 and (c) $H_2{}^{18}O^{[F]}$-HKUST-1 samples recorded at 298 K, removing pore-filling and coordinating H_2O molecules from the crystals under vacuum conditions. (d) distributions of Raman frequencies of Cu−Cu vibration, which depend on the state of Cu^{2+} node−activated, only coordinated and pore-filling.

Supplementary Section 9. Raman spectra of EtOH- and MeOH-filling HKUST-1 crystals measured at 298 K.

EtOH and MeOH confined in nanopores have shown a behaviour different from bulk in terms of their C−O and C−C vibrations[S8]. Whereas the C−C band of bulk EtOH appears at 885 cm^{-1}, the band of confined EtOH appears at lower energies of 876−881 cm^{-1} depending on the EtOH state. The pattern of C−O vibrational modes was also similar to that of C−C mode. Whereas the C−O bands of bulk EtOH appear at 1053 and 1097 cm^{-1}, the bands of confined EtOH shifted to lower energies of 1044−1049 and 1085−1094 cm^{-1}, respectively. The C−O vibration of MeOH[F]-HKUST-1 has also shown a similar trend as EtOH. Whereas the C−O band of bulk MeOH appear at 1035 cm^{-1}, the band of confined MeOH appeared at lower energies of 1011−1019cm^{-1}. Given that the binding of a molecule governs its vibrational energies, the above red-shifts can be ascribed to enhanced H-bond strength of confined EtOH and MeOH molecules (see Supplementary Figure 14).

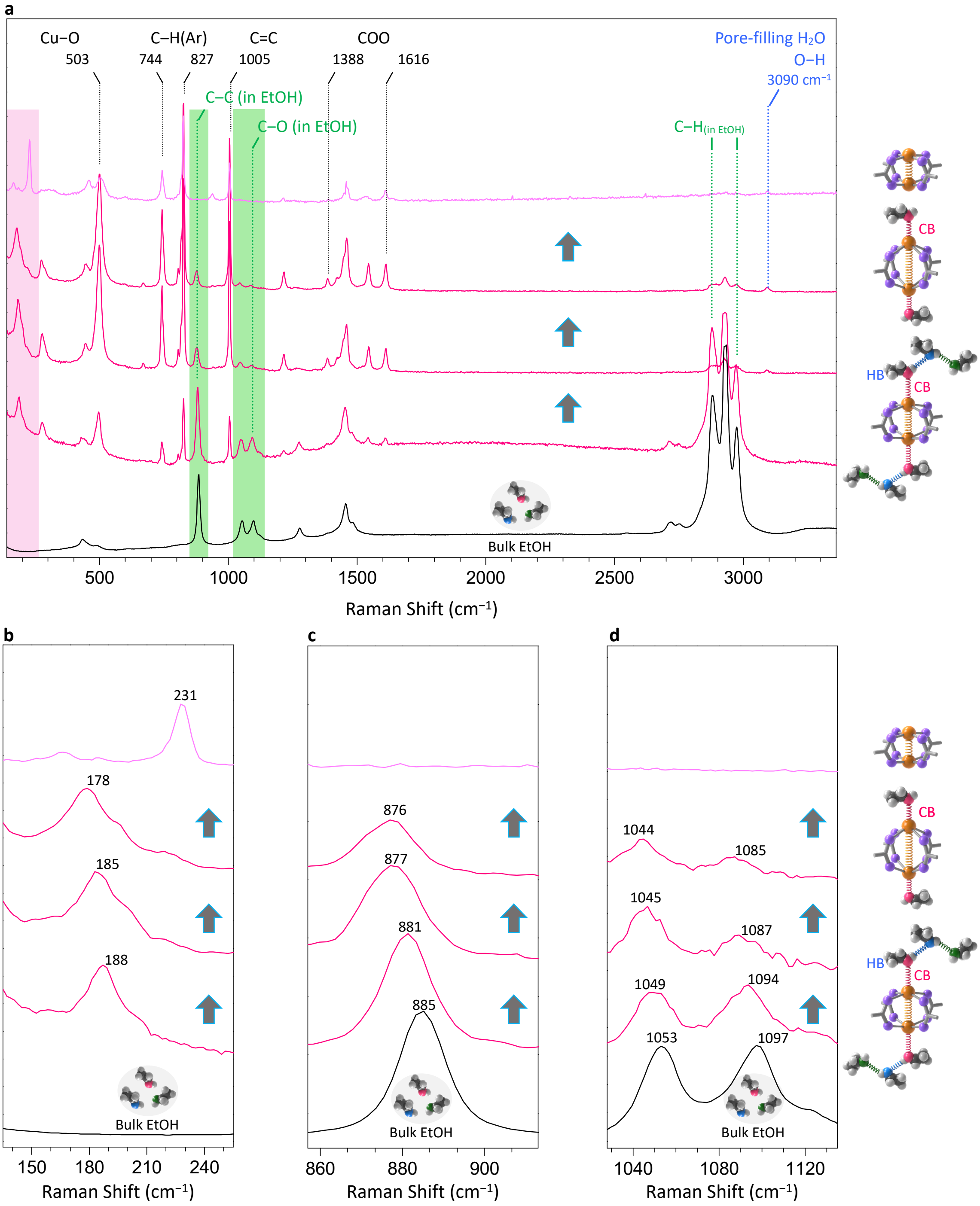

Supplementary Figure 12. (a) Wide and (b-d) expanded views of Raman spectra of an EtOH[F]-HKUST-1 sample recorded at 298 K, removing pore-filling and coordinating EtOH molecules from the crystals under vacuum conditions. Raman spectrum of the bulk EtOH was also taken for comparison. Raman spectrum of the bulk EtOH agrees well with the spectrum reported previously in the literature[S8].

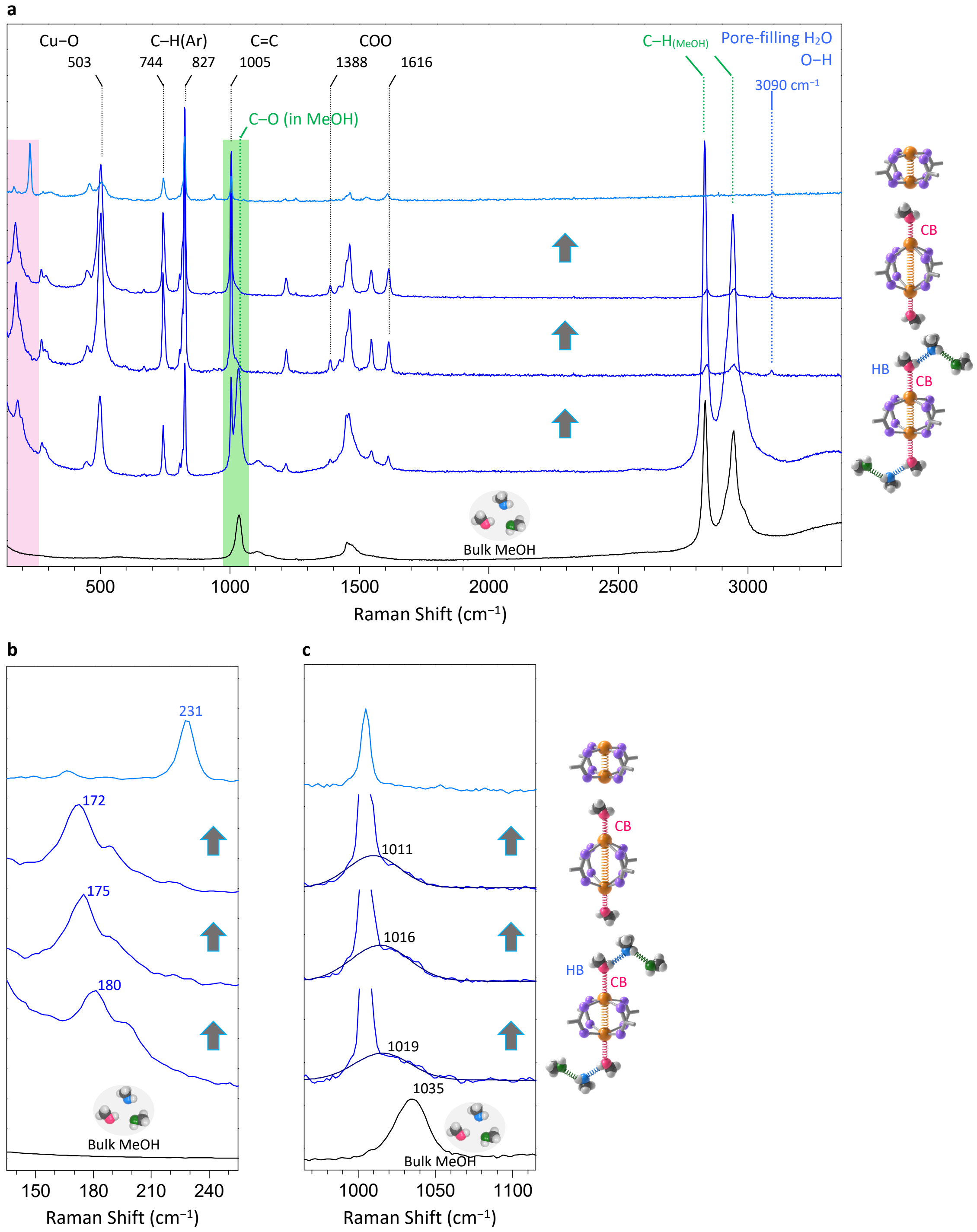

Supplementary Figure 13. (a) Wide and (b-c) expanded views of Raman spectra of a MeOH[F]-HKUST-1 sample recorded at 298 K, removing pore-filling and coordinating MeOH molecules from the crystals under vacuum conditions. Raman spectrum of the bulk MeOH was also taken for comparison. Raman spectrum of the bulk MeOH agrees well with the spectrum reported previously in the literature[S8].

Supplementary Section 10. Raman spectra of bulk methanol measured at 20 and -110 °C.

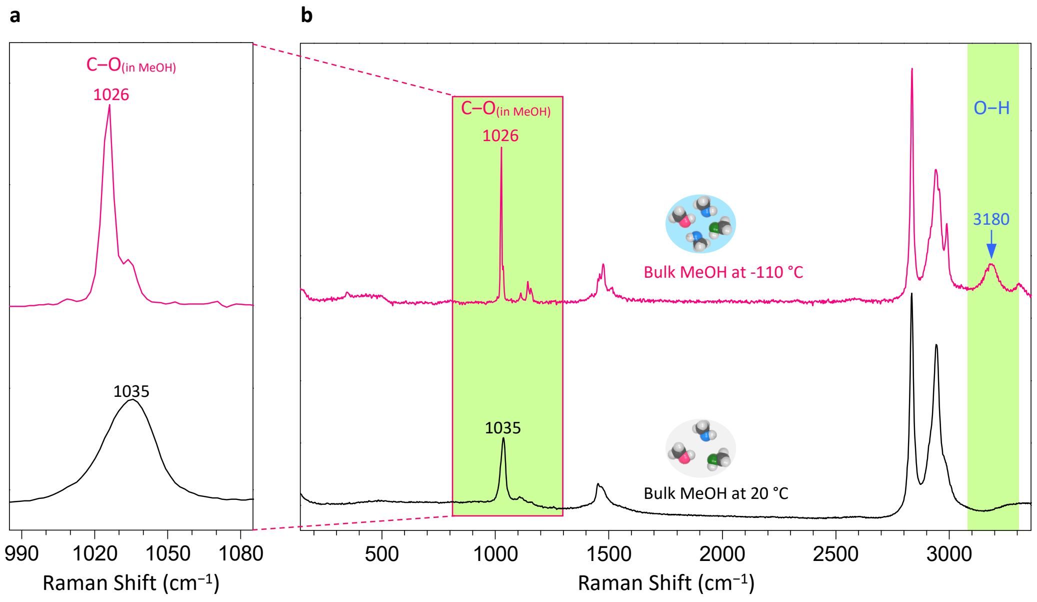

Supplementary Figure 14. (a) Expanded and (b) broad views of Raman spectra of bulk MeOH recorded at 20 and -110 °C, respectively, as indicated.

Supplementary Section 11. In situ Raman spectra of an HKUST-1 crystal measured at room temperature.

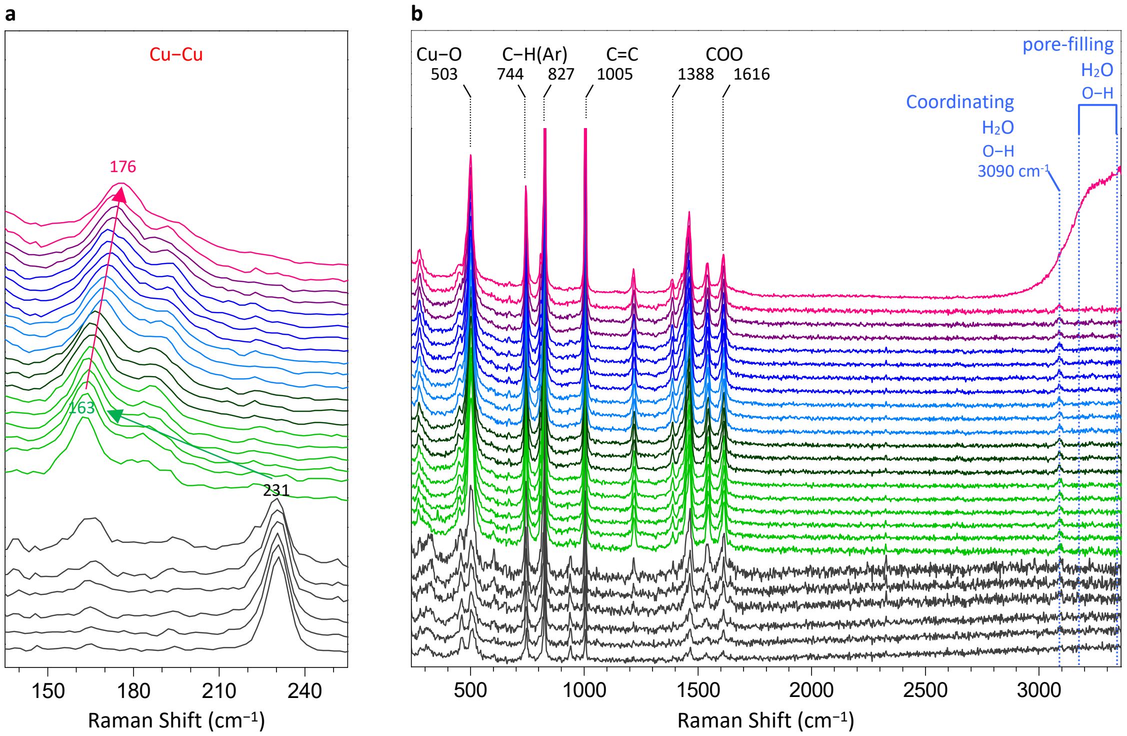

cc

Supplementary Figure 15. (a) Expanded and (b) broad views of successive in situ confocal micro-Raman spectra of an Act-HKUST-1 sample recorded at room temperature, infusing H_2O molecules into the crystal under humid conditions.

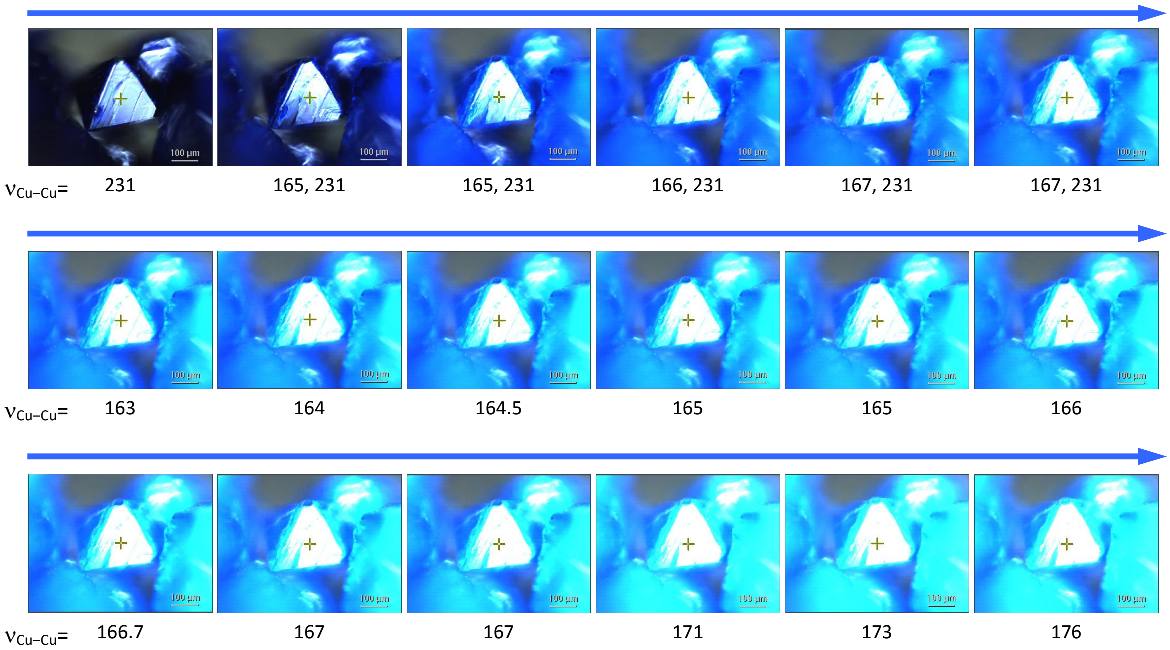

Supplementary Figure 16. Successive optical microscope images of an Act-HKUST-1 crystal taken during the measurements of the confocal micro-Raman spectra.

Supplementary Section 12. In situ infra-red spectra of an HKUST-1 crystal measured at room temperature.

An insightful reviewer asked us to demonstrate the presence of water in the air-exposed HKUST-1 samples and figure out the coordination of $Cu–O_{H2O}$. To address these questions, we have tried to observe the Cu–O bond vibrations directly by taking IR spectroscopy, where the vibrations appear at approximately 450 – 520 cm^{-1}. Unfortunately, however, we could not observe the $Cu–O_{H2O}$ vibration because of the noisy signal in 450 – 550 cm^{-1} region with the limit of reliable energies capable with commercial IR equipment. Instead, we subtracted the Act-HKUST-1 spectrum from each H_2O-HKUST-1 (10, 30, and 60 min) spectrum, respectively, to find a difference in the 500 – 600 cm^{-1} region[S9]. Then, we found two bands at approximately 550 and 595 cm^{-1}. Thus, we ascribe the two bands to the vibration of $Cu–O_{H2O}$. Also, we have observed the appearance of broad O–H vibration of water molecules, where the broadness comes from the hydrogen bondings (we speculate these hydrogen bondings are formed between coordinating water and pore-filling water and between pore-filling waters). Specifically, the Act-HKUST-1 does not show the broad vibration initially, but the band begins to appear instantaneously after the exposure to moist air. This pattern has also been observed in Raman spectra. (See Raman spectra in Supplementary Sections 6, 7, and 11.)

On the other hand, we observed the $Cu–O_{H2O}$ bond vibrations from the Raman spectra. As the Act-HKUST-1 is exposed to humid air, the band at 503 cm^{-1} begins to grow up in terms of its intensity, indicating that the amount of coordinating water increases as the exposure time increases. (See Supplementary Sections 6, 7, and 11.)

Conclusively, based on the X-ray diffraction results and Raman and IR spectroscopic results, we are convinced that, while Act-HKUST-1 does not include coordinating water, the moisture-exposed HKUST-1 does include the coordinating water at the OMSs.

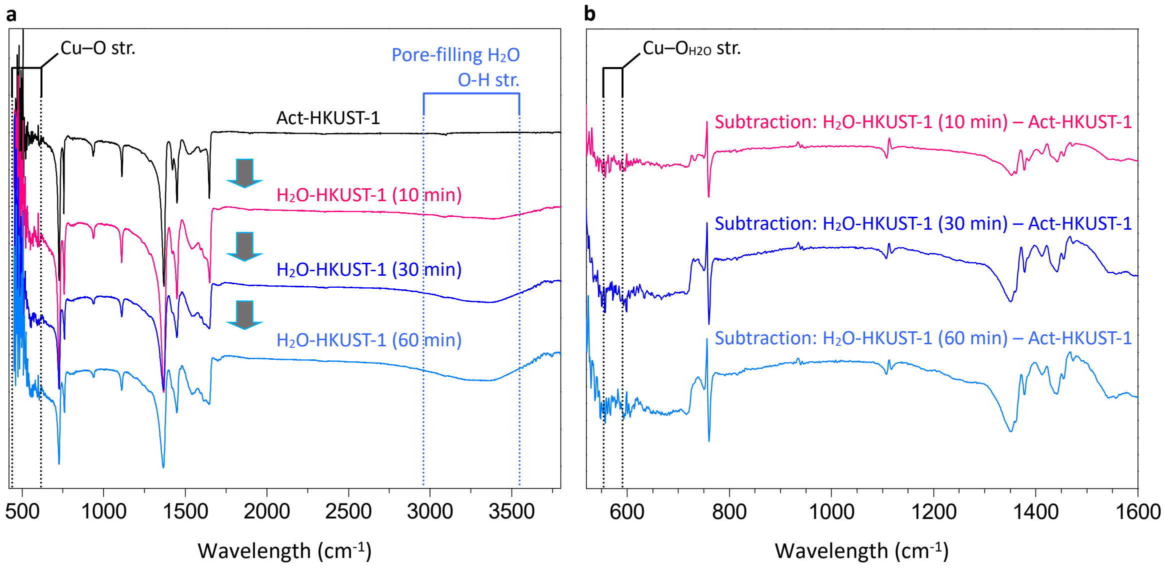

Supplementary Figure 17. (a) In situ infra-red spectra of activated and H_2O-exposed HKUST-1 crystal samples as indicated. (b) Difference spectra of H_2O-HKUST-1 samples obtained by subtracting the Act-HKUST-1 spectrum from each H_2O-HKUST-1 spectrum as indicated.

Supplementary Section 13. Theoretical number of hydrogen bondings around a coordinating H_2O molecule.

Supplementary Table 4. The theoretical maximum number of Cu–Cu nodes, Cu^{2+} ions, $H_2O^{[C]}$ and $H_2O^{[F]}$ in a unit cell of HKUST-1 and theoretical H_2O/Cu^{2+} ratio.

Component	Theoretical numbers	Ratio of H_2O/Cu^{2+}
Cu−Cu node	24	–
Cu^{2+} ion	48	–
[a]$H_2O^{[C]}$	48	1.00
[b]$H_2O^{[F]1}$	96	2.00
[b]$H_2O^{[F]2}$	96	2.00
[b]$H_2O^{[F]3}$	32	0.66_6

[a]$H_2O^{[C]}$ is Cu^{2+}-coordinating H_2O.

[b]$H_2O^{[F]1}$, $H_2O^{[F]2}$ and $H_2O^{[F]3}$ are type-1, -2 and -3 pore-filling H_2O molecules, respectively.

Supplementary Section 14. UV-vis absorption spectra of activated and water-filling HKUST-1s.

While the colour of the Act-HKUST-1 crystal is a deep navy blue, the colour was turned to pale blue as the moisture-exposure time increases (see Fig. 3c in the text and Supplementary Figure 16). Using UV-vis absorption spectroscopy, we confirmed that the colour change agrees well with the shift of the visible absorption band (see Supplementary Figure 18). The absorption band at wavelengths greater than approximately 500 nm (less than 2.5 eV in energies) is due to the d−d transition around Cu^{2+} centres. Thus, the aforementioned colour changes ascribe to the shift of the d−d transition, reflecting the change in the coordination environment of the Cu^{2+} centres from coordination-free to H_2O-coordinating state.

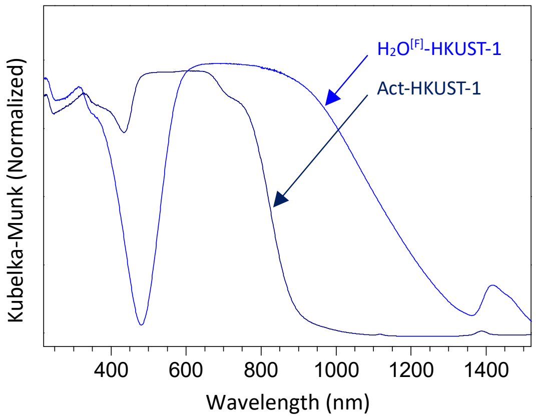

Supplementary Figure 18. Solid-state diffuse reflectance UV−vis absorption spectra of Act-HKUST-1 (black curve) and $H_2O^{[F]}$-HKUST-1 (blue curve). The absorption spectra were taken after the samples were contained in disc-shaped quartz cells.

Supplementary Section 15. In situ SCXRD data of HKUST-1 collected at 298 K.

In situ SCXRD measurements were performed with a custom-made vacuum manifold, specially designed gonio-head and a 50 μm gripper (MiTeGen LLC, USA) installed at BL2D-SMC in the PAL. Before collecting diffraction data, a single crystal placed in the gripper was outgassed at 423 K under vacuum conditions until residual electron densities for coordinated water molecules and the interior of the nanochannel disappeared. Then, the single crystal was allowed to cool to 298 K using a Cryojet 5 and kept at the temperature during the in situ SCXRD measurement. The in situ SCXRD data were collected at 298 K by using silicon (111) double crystal monochromator, synchrotron radiation with the wavelength (λ) of 0.63000 Å and Rayonix MX225HS detectors, exposing the crystal to moist air for 0, 5, 10, 15, 20, 30, 60, 120, 180 and 240 min in order to record the changes in the crystal structure during water sorption. Using PAL BL2D-SMDC software[S4], the diffraction data were collected every 2 seconds for a frame at the detector distance of 66 mm with the omega scan ($\Delta\omega$) of 3°. Also, a software of HKL3000sm (Ver. 720)[S5] was used for cell refinement, reduction and absorption correction. Then, the crystal structures of the crystals were solved by the intrinsic phasing method with the software of the SHELXT-2018[S6] and subsequently refined by full-matrix least-squares calculations with the SHELXL-2018[S7]. All atoms except hydrogen were refined, considering the atomic anisotropy. Hydrogen atoms bound to carbon atoms in the benzene moieties were considered to place at geometrically ideal positions and constrained to ride on their parent atoms with the C-H bond length of 0.94 Å and the U_{iso}(H) value of 1.2 U_{eq} for the parent atoms. The hydrogen atoms in the coordinated and pore-filling water molecules were found in difference-Fourier maps and restrained by using DFIX and DANG commands during the least-squares refinement with U_{iso}(H) values of 1.2U_{eq} of the oxygen atom. The water molecule occupancies in the crystal have been checked during least-squares refinement. Crystal data, data collection and structure refinement details are summarized in Supplementary Table 4−13.

(1) Activated HKUST-1 (Act-HKUST-1)

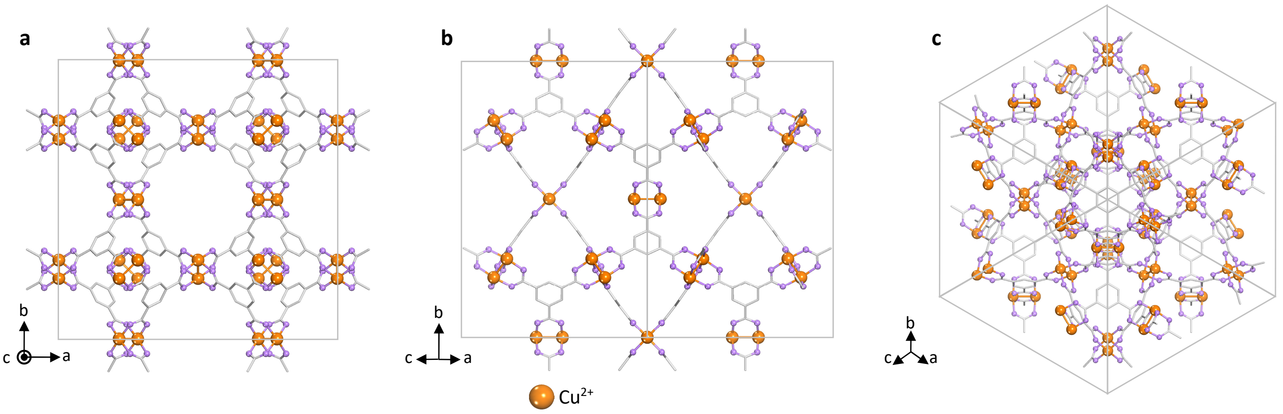

Supplementary Figure 19. (a) [001], (b) [101] and (c) [111] views of the SCXRD structure of an Act-HKUST-1 single-crystal recorded at 298 K. Hydrogen atoms bound to carbon atoms in benzene moieties are omitted for the sake of clarity.

Supplementary Table 5. Crystal data and structure refinement of the Act-HKUST-1 measured at 298 K.

**Identification code**	**Act-HKUST-1**	
Empirical formula	$C_{18} H_6 Cu_3 O_{12}$	
Formula weight	604.85	
Temperature	298(2) K	
Wavelength	0.630 Å	
Crystal system	Cubic	
Space group	Fm-3m	
	a = 26.249(3) Å	α = 90°
Unit cell dimensions	b = 26.249(3) Å	β = 90°
	c = 26.249(3) Å	γ = 90°
Cu−Cu distance	2.484 Å	
Volume	18086(6) Å^3	
Z	16	
Density (calculated)	0.889 Mg/m^3	
Absorption coefficient	1.027 mm^{-1}	
F(000)	4752	
Crystal size	0.054 × 0.052 × 0.050 mm^3	
Theta range for data collection	1.191 to 25.975°	
Index ranges	$-36 \leq h \leq 36$; $-36 \leq k \leq 36$; $-36 \leq l \leq 36$	
Reflections collected	46344	
Independent reflections	1323 [R(int) = 0.1856]	
Completeness to theta = 22.210°	98.9%	
Absorption correction	Empirical	
Max. and min. transmission	1.000 and 0.824	
Refinement method	Full-matrix least-squares on F^2	
Data / restraints / parameters	1323 / 0 / 32	
Goodness-of-fit on F^2	1.031	
Final R indices [I>2sigma(I)]	R1 = 0.0548, wR2 = 0.1565	
R indices (all data)	R1 = 0.0817, wR2 = 0.1707	
Extinction coefficient	n/a	
Largest diff. peak and hole	0.499 and -0.493 e.Å^{-3}	

(2) Moisture-exposed HKUST-1 for 5 min (H_2O-HKUST-1(1st))

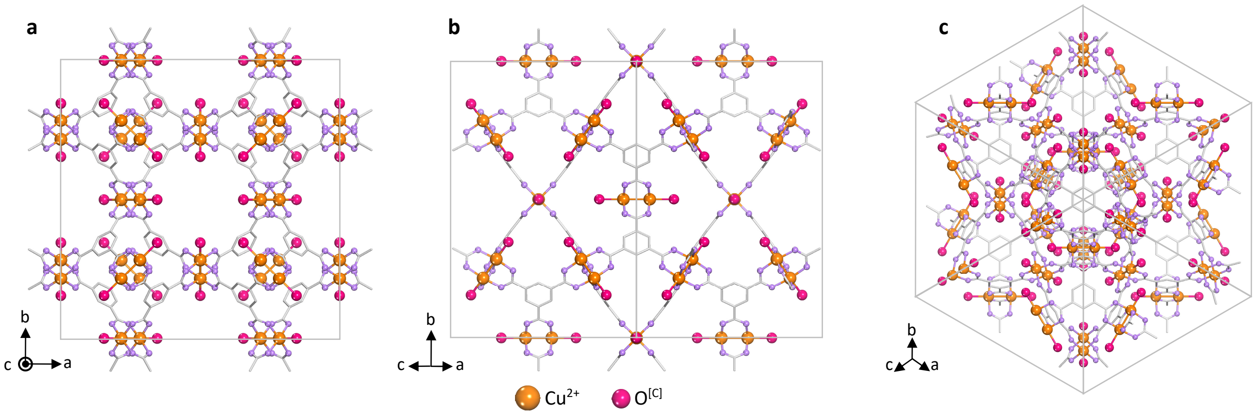

Supplementary Figure 20. (a) [001], (b) [101] and (c) [111] views of the SCXRD structure of an Act-HKUST-1 single-crystal recorded at 298 K after exposure to moist air for 5 min. Hereafter we denote it as H_2O-HKUST-1(1st). Hydrogen atoms bound to carbon atoms in benzene moieties and coordinated water molecules are omitted for the sake of clarity.

Supplementary Table 6. Crystal data and structure refinement of the H_2O-HKUST-1(1st) measured at 298 K.

**Identification code**	**H_2O-HKUST-1(1st)**	
Empirical formula	$C_{18}H_{6.60}Cu_3O_{12.30}$	
Formula weight	610.25	
Temperature	298(2) K	
Wavelength	0.630 Å	
Crystal system	Cubic	
Space group	Fm-3m	
Unit cell dimensions	a = 26.275(3) Å	α = 90°
	b = 26.275(3) Å	β = 90°
	c = 26.275(3) Å	γ = 90°
Cu−Cu distance	2.505	
Volume	18139(6) Å^3	
Z	16	
Density (calculated)	0.894 Mg/m^3	
Absorption coefficient	1.025 mm^{-1}	
F(000)	4800	
Crystal size	0.054 × 0.052 × 0.050 mm^3	
Theta range for data collection	2.749 to 25.989°	
Index ranges	$-36 \leq h \leq 36$; $-36 \leq k \leq 36$; $-36 \leq l \leq 36$	
Reflections collected	46856	
Independent reflections	1329 [R(int) = 0.1930]	
Completeness to theta = 22.210°	99.2%	
Absorption correction	Empirical	
Max. and min. transmission	1.000 and 0.786	
Refinement method	Full-matrix least-squares on F^2	
Data / restraints / parameters	1329 / 9 / 38	
Goodness-of-fit on F^2	1.052	
Final R indices [I>2sigma(I)]	R1 = 0.0638, wR2 = 0.1795	
R indices (all data)	R1 = 0.0956, wR2 = 0.1979	
Extinction coefficient	n/a	
Largest diff. peak and hole	0.559 and -0.331 e.Å^{-3}	

(3) Moisture-exposed HKUST-1 for 10 min (H_2O-HKUST-1(2nd))

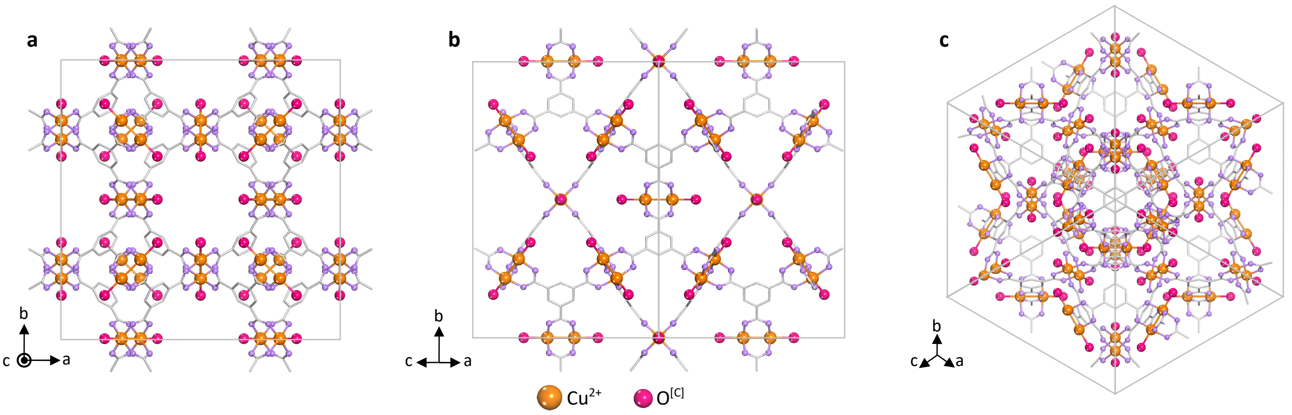

Supplementary Figure 21. (a) [001], (b) [101] and (c) [111] views of the SCXRD structure of an Act-HKUST-1 single-crystal recorded at 298 K after exposure to moist air for 10 min. Hereafter we denote it as H_2O-HKUST-1(2nd). Hydrogen atoms bound to carbon atoms in benzene moieties and coordinated water molecules are omitted for the sake of clarity.

Supplementary Table 7. Crystal data and structure refinement of the H_2O-HKUST-1(2nd) measured at 298 K.

**Identification code**	**H_2O-HKUST-1(2nd)**	
Empirical formula	C_{18} $H_{10.56}$ Cu_3 $O_{14.28}$	
Formula weight	645.92	
Temperature	298(2) K	
Wavelength	0.630 Å	
Crystal system	Cubic	
Space group	Fm-3m	
	a = 26.303(3) Å	α = 90°
Unit cell dimension	b = 26.303(3) Å	β = 90°
	c = 26.303(3) Å	γ = 90°
Cu−Cu distance	2.561 Å	
Volume	18197(6) $Å^3$	
Z	16	
Density (calculated)	0.943 Mg/m^3	
Absorption coefficient	1.026 mm^{-1}	
F(000)	5117	
Crystal size	0.054 × 0.052 × 0.050 mm^3	
Theta range for data collection	2.276 to 25.991°	
Index ranges	$-36 \leq h \leq 36$; $-36 \leq k \leq 36$; $-36 \leq l \leq 36$	
Reflections collected	47029	
Independent reflections	1331 [R(int) = 0.1963]	
Completeness to theta = 22.210°	99.1%	
Absorption correction	Empirical	
Max. and min. transmission	1.000 and 0.809	
Refinement method	Full-matrix least-squares on F^2	
Data / restraints / parameters	1331 / 9 / 38	
Goodness-of-fit on F^2	1.006	
Final R indices [I>2sigma(I)]	R1 = 0.0649, wR2 = 0.1961	
R indices (all data)	R1 = 0.1025, wR2 = 0.2210	
Extinction coefficient	n/a	
Largest diff. peak and hole	0.525 and -0.318 e.$Å^{-3}$	

(4) Moisture-exposed HKUST-1 for 15 min (H_2O-HKUST-1(3rd))

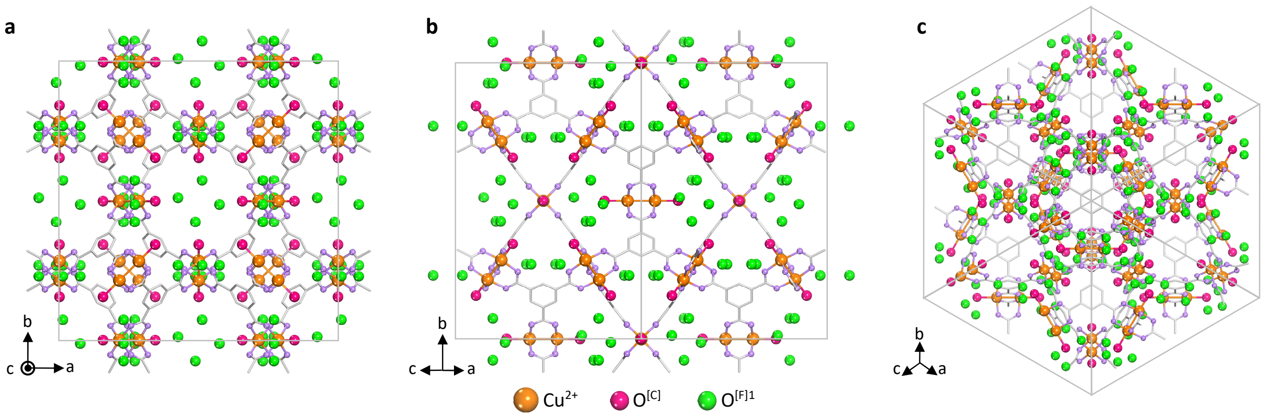

Supplementary Figure 22. (a) [001], (b) [101] and (c) [111] views of the SCXRD structure of an Act-HKUST-1 single-crystal recorded at 298 K after exposure to moist air for 15 min. Hereafter we denote it as H_2O-HKUST-1(3rd). Hydrogen atoms bound to carbon atoms in benzene moieties and water molecules are omitted for the sake of clarity.

Supplementary Table 8. Crystal data and structure refinement of the H_2O-HKUST-1(3rd) measured at 298 K.

**Identification code**	**H_2O-HKUST-1(3rd)**	
Empirical formula	$C_{18} H_{13.68} Cu_3 O_{15.84}$	
Formula weight	674.03	
Temperature	298(2) K	
Wavelength	0.630 Å	
Crystal system	Cubic	
Space group	Fm-3m	
	a = 26.282(3) Å	α = 90°
Unit cell dimensions	b = 26.282(3) Å	β = 90°
	c = 26.282(3) Å	γ = 90°
Cu−Cu distance	2.605	
Volume	18155(6) $Å^3$	
Z	16	
Density (calculated)	0.986 Mg/m^3	
Absorption coefficient	1.032 mm^{-1}	
F(000)	5366	
Crystal size	0.054 × 0.052 × 0.050 mm^3	
Theta range for data collection	2.748 to 25.981°	
Index ranges	$-36 \leq h \leq 36$; $-36 \leq k \leq 36$; $-36 \leq l \leq 36$	
Reflections collected	46830	
Independent reflections	1324 [R(int) = 0.2113]	
Completeness to theta = 22.210°	98.6%	
Absorption correction	Empirical	
Max. and min. transmission	1.000 and 0.787	
Refinement method	Full-matrix least-squares on F^2	
Data / restraints / parameters	1324 / 18 / 53	
Goodness-of-fit on F^2	0.996	
Final R indices [I>2sigma(I)]	R1 = 0.0699, wR2 = 0.2200	
R indices (all data)	R1 = 0.1176, wR2 = 0.2521	
Extinction coefficient	n/a	
Largest diff. peak and hole	0.553 and -0.379 e.$Å^{-3}$	

(5) Moisture-exposed HKUST-1 for 20 min (H_2O-HKUST-1(4th))

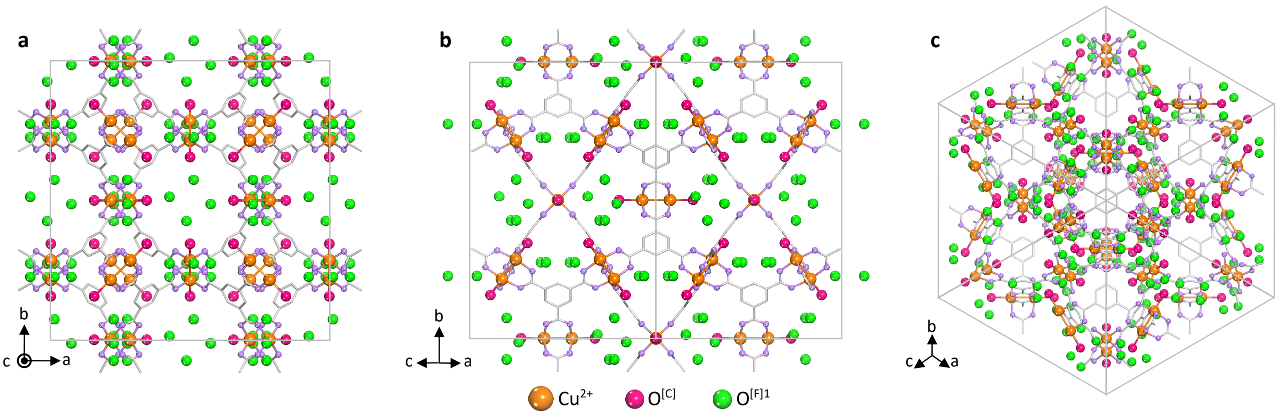

Supplementary Figure 23. (a) [001], (b) [101] and (c) [111] views of the SCXRD structure of an Act-HKUST-1 single-crystal recorded at 298 K after exposure to moist air for 20 min. Hereafter we denote it as H_2O-HKUST-1(4th). Hydrogen atoms bound to carbon atoms in benzene moieties and water molecules are omitted for the sake of clarity.

Supplementary Table 9. Crystal data and structure refinement of the H_2O-HKUST-1(4th) measured at 298 K.

**Identification code**	**H_2O-HKUST-1(4th)**	
Empirical formula	$C_{18} H_{14.40} Cu_3 O_{16.20}$	
Formula weight	680.51	
Temperature	298(2) K	
Wavelength	0.630 Å	
Crystal system	Cubic	
Space group	Fm-3m	
	a = 26.296(3) Å	α = 90°
Unit cell dimensions	b = 26.296(3) Å	β = 90°
	c = 26.296(3) Å	γ = 90°
Cu−Cu distance	2.621	
Volume	18184(6) Å^3	
Z	16	
Density (calculated)	0.994 Mg/m^3	
Absorption coefficient	1.031 mm^{-1}	
F(000)	5424	
Crystal size	0.054 × 0.052 × 0.050 mm^3	
Theta range for data collection	1.373 to 25.998°	
Index ranges	$-36 \leq h \leq 36$; $-36 \leq k \leq 36$; $-36 \leq l \leq 36$	
Reflections collected	46990	
Independent reflections	1326 [R(int) = 0.2499]	
Completeness to theta = 22.210°	98.4%	
Absorption correction	Empirical	
Max. and min. transmission	1.000 and 0.836	
Refinement method	Full-matrix least-squares on F^2	
Data / restraints / parameters	1326 / 18 / 53	
Goodness-of-fit on F^2	0.941	
Final R indices [I>2sigma(I)]	R1 = 0.0841, wR2 = 0.2496	
R indices (all data)	R1 = 0.1469, wR2 = 0.2930	
Extinction coefficient	n/a	
Largest diff. peak and hole	0.612 and -0.435 e.Å^{-3}	

(6) Moisture-exposed HKUST-1 for 30 min (H_2O-HKUST-1(5th))

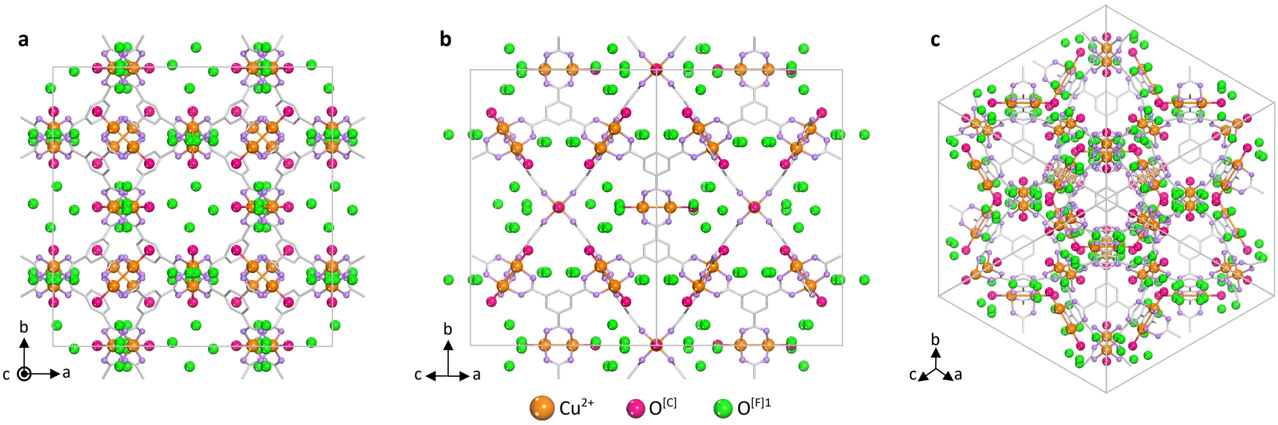

Supplementary Figure 24. (a) [001], (b) [101] and (c) [111] views of the SCXRD structure of an Act-HKUST-1 single-crystal recorded at 298 K after exposure to moist air for 30 min. Hereafter we denote it as H_2O-HKUST-1(5th). Hydrogen atoms bound to carbon atoms in benzene moieties and water molecules are omitted for the sake of clarity.

Supplementary Table 10. Crystal data and structure refinement of the H_2O-HKUST-1(5th) measured at 298 K.

**Identification code**	**H_2O-HKUST-1(5th)**	
Empirical formula	$C_{18}H_{15.36}Cu_3O_{16.68}$	
Formula weight	689.16	
Temperature	298(2) K	
Wavelength	0.630 Å	
Crystal system	Cubic	
Space group	Fm-3m	
	a = 26.243(3) Å	α = 90°
Unit cell dimensions	b = 26.243(3) Å	β = 90°
	c = 26.243(3) Å	γ = 90°
Cu−Cu distance	2.626 Å	
Volume	18073(6) $Å^3$	
Z	16	
Density (calculated)	1.013 Mg/m^3	
Absorption coefficient	1.038 mm^{-1}	
F(000)	5501	
Crystal size	0.054 × 0.052 × 0.050 mm^3	
Theta range for data collection	1.376 to 25.981°	
Index ranges	$-36 \leq h \leq 36$; $-36 \leq k \leq 36$; $-36 \leq l \leq 36$	
Reflections collected	46269	
Independent reflections	1321 [R(int) = 0.3242]	
Completeness to theta = 22.210°	98.8%	
Absorption correction	Empirical	
Max. and min. transmission	1.000 and 0.829	
Refinement method	Full-matrix least-squares on F^2	
Data / restraints / parameters	1321 / 19 / 53	
Goodness-of-fit on F^2	0.965	
Final R indices [I>2sigma(I)]	R1 = 0.0916, wR2 = 0.2900	
R indices (all data)	R1 = 0.1844, wR2 = 0.3539	
Extinction coefficient	n/a	
Largest diff. peak and hole	0.582 and -0.412 e.$Å^{-3}$	

(7) Moisture-exposed HKUST-1 for 60 min (H_2O-HKUST-1(6th))

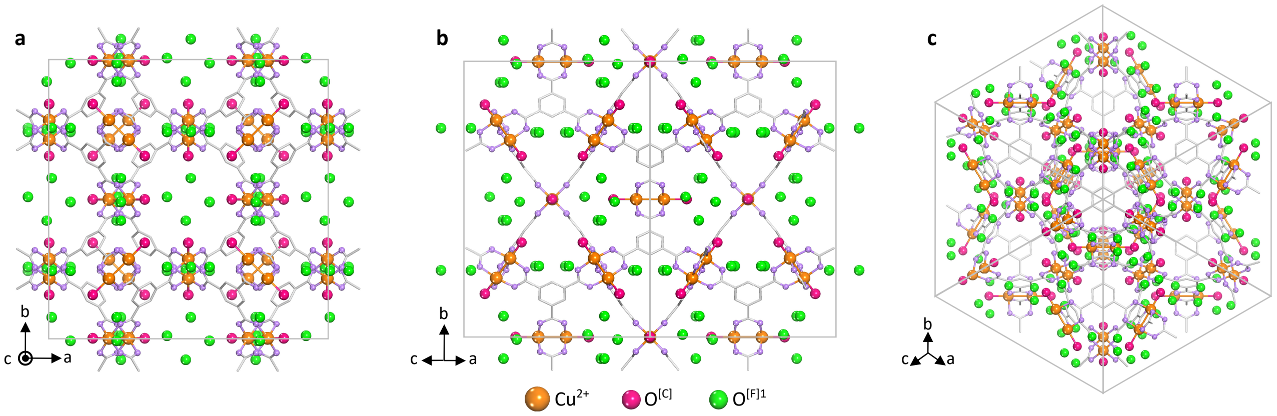

Supplementary Figure 25. (a) [001], (b) [101] and (c) [111] views of the SCXRD structure of an Act-HKUST-1 single-crystal recorded at 298 K after exposure to moist air for 60 min. Hereafter we denote it as H_2O-HKUST-1(6th). Hydrogen atoms bound to carbon atoms in benzene moieties and water molecules are omitted for the sake of clarity.

Supplementary Table 11. Crystal data and structure refinement of the H_2O-HKUST-1(6th) measured at 298 K.

**Identification code**	**H_2O-HKUST-1(6th)**	
Empirical formula	$C_{18} H_{16.08} Cu_3 O_{17.04}$	
Formula weight	695.65	
Temperature	298(2) K	
Wavelength	0.630 Å	
Crystal system	Cubic	
Space group	Fm-3m	
	a = 26.245(3) Å	α = 90°
Unit cell dimensions	b = 26.245(3) Å	β = 90°
	c = 26.245(3) Å	γ = 90°
Cu−Cu distance	2.632 Å	
Volume	18078(6) $Å^3$	
Z	16	
Density (calculated)	1.022 Mg/m^3	
Absorption coefficient	1.039 mm^{-1}	
F(000)	5558	
Crystal size	0.054 × 0.052 × 0.050 mm^3	
Theta range for data collection	2.281 to 25.979°	
Index ranges	$-36 \le h \le 36$; $-36 \le k \le 36$; $-36 \le l \le 36$	
Reflections collected	46014	
Independent reflections	1319 [R(int) = 0.4030]	
Completeness to theta = 22.210°	98.6%	
Absorption correction	Empirical	
Max. and min. transmission	1.000 and 0.821	
Refinement method	Full-matrix least-squares on F^2	
Data / restraints / parameters	1319 / 19 / 53	
Goodness-of-fit on F^2	0.939	
Final R indices [I>2sigma(I)]	R1 = 0.0991, wR2 = 0.2927	
R indices (all data)	R1 = 0.2267, wR2 = 0.3761	
Extinction coefficient	n/a	
Largest diff. peak and hole	0.563 and -0.477 e.$Å^{-3}$	

(8) Moisture-exposed HKUST-1 for 120 min (H_2O-HKUST-1(7th))

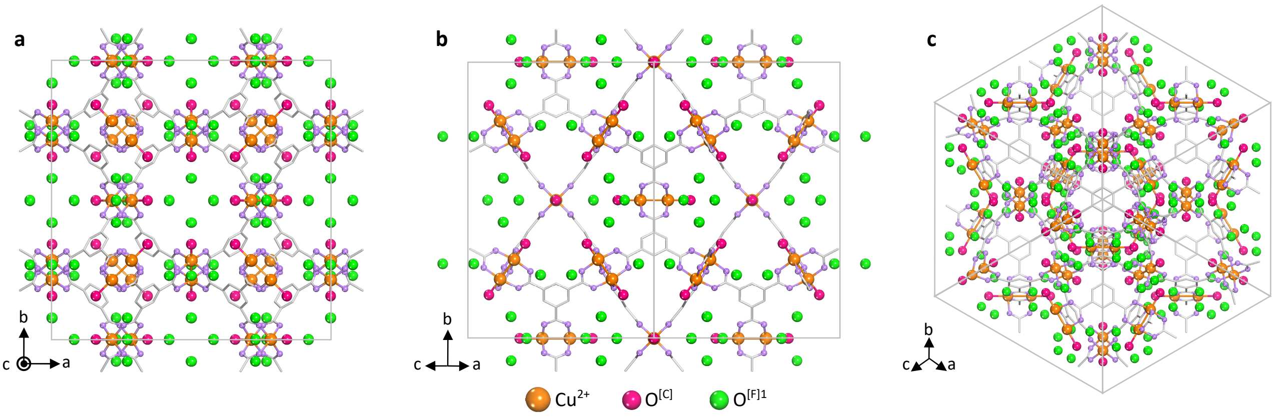

Supplementary Figure 26. (a) [001], (b) [101] and (c) [111] views of the SCXRD structure of an Act-HKUST-1 single-crystal recorded at 298 K after exposure to moist air for 120 min. Hereafter we denote it as H_2O-HKUST-1(7th). Hydrogen atoms bound to carbon atoms in benzene moieties and water molecules are omitted for the sake of clarity.

Supplementary Table 12. Crystal data and structure refinement of the H_2O-HKUST-1(7th) measured at 298 K.

**Identification code**	**H_2O-HKUST-1(7th)**	
Empirical formula	$C_{18}H_{18.72}Cu_3O_{18.36}$	
Formula weight	719.43	
Temperature	298(2) K	
Wavelength	0.630 Å	
Crystal system	Cubic	
Space group	Fm-3m	
	a = 26.265(3) Å	α = 90°
Unit cell dimensions	b = 26.265(3) Å	β = 90°
	c = 26.265(3) Å	γ = 90°
Cu–Cu distance	2.622 Å	
Volume	18118(6) Å^3	
Z	16	
Density (calculated)	1.055 Mg/m^3	
Absorption coefficient	1.039 mm^{-1}	
F(000)	5770	
Crystal size	0.054 × 0.052 × 0.050 mm^3	
Theta range for data collection	1.190 to 26.000°	
Index ranges	$-36 \leq h \leq 36$; $-36 \leq k \leq 36$; $-36 \leq l \leq 36$	
Reflections collected	46041	
Independent reflections	1319 [R(int) = 0.3929]	
Completeness to theta = 22.210°	98.5%	
Absorption correction	Empirical	
Max. and min. transmission	1.000 and 0.834	
Refinement method	Full-matrix least-squares on F^2	
Data / restraints / parameters	1319 / 19 / 47	
Goodness-of-fit on F^2	1.014	
Final R indices [I>2sigma(I)]	R1 = 0.1144, wR2 = 0.3225	
R indices (all data)	R1 = 0.2286, wR2 = 0.3950	
Extinction coefficient	n/a	
Largest diff. peak and hole	0.662 and -0.323 e.Å^{-3}	

(9) Moisture-exposed HKUST-1 for 180 min (H_2O-HKUST-1(8th))

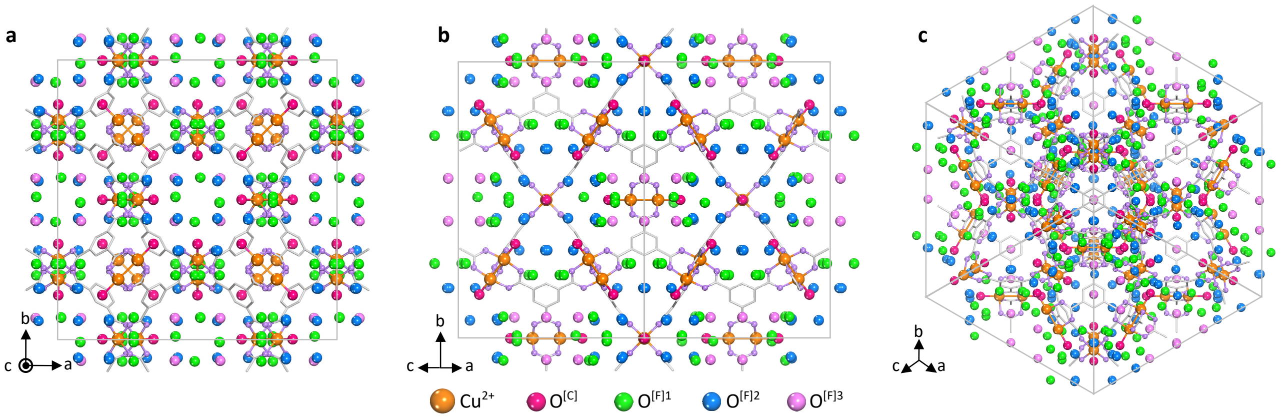

Supplementary Figure 27. (a) [001], (b) [101] and (c) [111] views of the SCXRD structure of an Act-HKUST-1 single-crystal recorded at 298 K after exposure to moist air for 180 min. Hereafter we denote it as H_2O-HKUST-1(8th). Hydrogen atoms bound to carbon atoms in benzene moieties and water molecules are omitted for the sake of clarity.

Supplementary Table 13. Crystal data and structure refinement of the H_2O-HKUST-1(8th) measured at 298 K.

**Identification code**	**H_2O-HKUST-1(8th)**	
Empirical formula	$C_{18} H_{24.48} Cu_3 O_{21.24}$	
Formula weight	771.31	
Temperature	298(2) K	
Wavelength	0.630 Å	
Crystal system	Cubic	
Space group	Fm-3m	
	a = 26.393(3) Å	α = 90°
Unit cell dimensions	b = 26.393(3) Å	β = 90°
	c = 26.393(3) Å	γ = 90°
Cu−Cu distance	2.619 Å	
Volume	18385(6) $Å^3$	
Z	16	
Density (calculated)	1.115 Mg/m^3	
Absorption coefficient	1.030 mm^{-1}	
F(000)	6230	
Crystal size	0.054 x 0.052 x 0.050 mm^3	
Theta range for data collection	1.185 to 25.989°	
Index ranges	$-36 \leq h \leq 36$; $-36 \leq k \leq 36$; $-36 \leq l \leq 36$	
Reflections collected	46968	
Independent reflections	1334 [R(int) = 0.3525]	
Completeness to theta = 22.210°	98.4%	
Absorption correction	Empirical	
Max. and min. transmission	1.000 and 0.815	
Refinement method	Full-matrix least-squares on F^2	
Data / restraints / parameters	1334 / 37 / 65	
Goodness-of-fit on F^2	1.035	
Final R indices [I>2sigma(I)]	R1 = 0.1070, wR2 = 0.3189	
R indices (all data)	R1 = 0.1989, wR2 = 0.3769	
Extinction coefficient	n/a	
Largest diff. peak and hole	0.599 and -0.419 e.$Å^{-3}$	

(10) Moisture-exposed HKUST-1 for 240 min (H_2O-HKUST-1(9th))

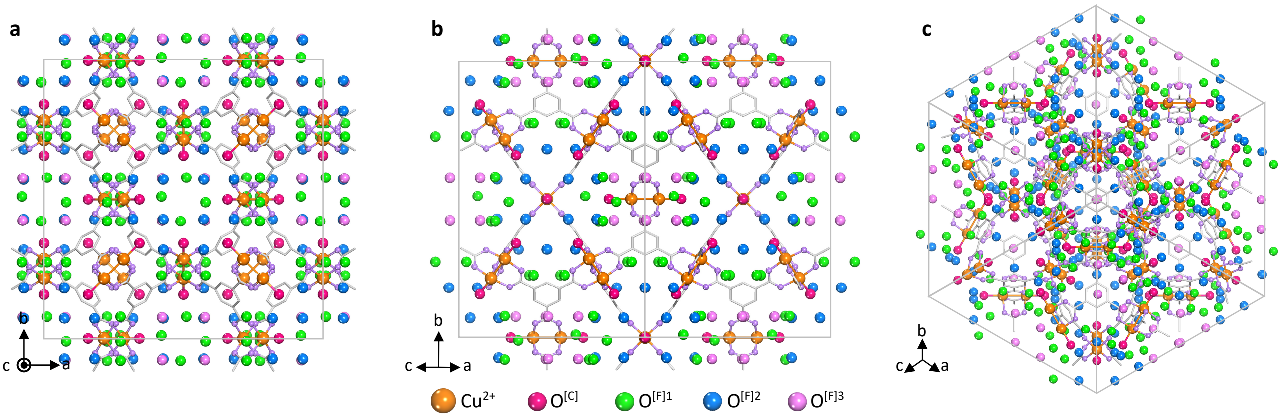

Supplementary Figure 28. (a) [001], (b) [101] and (c) [111] views of the SCXRD structure of an Act-HKUST-1 single-crystal recorded at 298 K after exposure to moist air for 240 min. Hereafter we denote it as H_2O-HKUST-1(9th). Hydrogen atoms bound to carbon atoms in benzene moieties and water molecules are omitted for the sake of clarity.

Supplementary Table 14. Crystal data and structure refinement of the H_2O-HKUST-1(9th) measured at 298 K.

**Identification code**	**H_2O-HKUST-1(9th)**	
Empirical formula	$C_{18}H_{29.04}Cu_3O_{23.52}$	
Formula weight	812.39	
Temperature	298(2) K	
Wavelength	0.630 Å	
Crystal system	Cubic	
Space group	Fm-3m	
	a = 26.399(3) Å	α = 90°
Unit cell dimensions	b = 26.399(3) Å	β = 90°
	c = 26.399(3) Å	γ = 90°
Cu−Cu distance	2.617 Å	
Volume	18398(6) Å^3	
Z	16	
Density (calculated)	1.173 Mg/m^3	
Absorption coefficient	1.035 mm^{-1}	
F(000)	6595	
Crystal size	0.054 x 0.052 x 0.050 mm^3	
Theta range for data collection	1.184 to 25.982°	
Index ranges	-36 ≤ h ≤ 36; -36 ≤ k ≤ 36; -36 ≤ l ≤ 36	
Reflections collected	46654	
Independent reflections	1332 [R(int) = 0.3442]	
Completeness to theta = 22.210°	98.4%	
Absorption correction	Empirical	
Max. and min. transmission	1.000 and 0.857	
Refinement method	Full-matrix least-squares on F^2	
Data / restraints / parameters	1332 / 37 / 62	
Goodness-of-fit on F^2	0.983	
Final R indices [I>2sigma(I)]	R1 = 0.1042, wR2 = 0.2995	
R indices (all data)	R1 = 0.1924, wR2 = 0.3557	
Extinction coefficient	n/a	
Largest diff. peak and hole	0.603 and -0.465 e.Å^{-3}	

Supplementary Section 16. Summary of the HKUST-1 crystal structure obtained from in situ SCXRD.

Supplementary Table 15. Summary for Cu–Cu and Cu–O[C] length (Å), O_{BTC}–Cu–O_{BTC} angle (°), and H_2O occupancies (mol%) of an Act-HKUST-1 crystal collected from the in situ SCXRD data at 298 K, simultaneously exposing it to moisture.

Samples	Exposure time (min)	Length (Å)		Angle (°)	Occupancy of water molecules (mol%)			
		Cu–Cu	Cu–O[C]a	∠O_{BTC}–Cu–O_{BTC}	H_2O[C]b	H_2O[F]1c	H_2O[F]2c	H_2O[F]3c
Act-HKUST-1	0	2.484	–	172.52	0	0	0	0
H_2O-HKUST-1(1st)	5	2.505	2.289	171.60	10	0	0	0
H_2O-HKUST-1(2nd)	10	2.561	2.242	170.51	76	0	0	0
H_2O-HKUST-1(3rd)	15	2.605	2.184	169.40	96	16	0	0
H_2O-HKUST-1(4th)	20	2.621	2.193	168.78	100	20	0	0
H_2O-HKUST-1(5th)	30	2.626	2.164	169.42	100	28	0	0
H_2O-HKUST-1(6th)	60	2.632	2.151	169.52	100	34	0	0
H_2O-HKUST-1(7th)	120	2.622	2.136	168.93	100	56	0	0
H_2O-HKUST-1(8th)	180	2.619	2.106	169.33	100	56	40	24
H_2O-HKUST-1(9th)	240	2.617	2.130	168.78	100	86	50	18

[a]O[C] is oxygen in Cu^{2+}-coordinating H_2O.
[b]H_2O[C] is Cu^{2+}-coordinating H_2O.
[c]H_2O[F]1, H_2O[F]2 and H_2O[F]3 are type-1, -2 and -3 pore-filling H_2O molecules, respectively.

Supplementary Table 16. Summary for O–O distance (Å) in an Act-HKUST-1 crystal collected from the in situ SCXRD data at 298 K, simultaneously exposing it to moisture.

Samples	Exposure time (min)	Oxygen–oxygen distance (Å)					
		O[C]–O[F]1	O[C]–O[F]2	O[F]1–O[F]1	O[F]1–O[F]2	O[F]2–O[F]2	[d]O[F]3–O[F]3
Act-HKUST-1	0						
H_2O-HKUST-1(1st)	5						
H_2O-HKUST-1(2nd)	10						
H_2O-HKUST-1(3rd)	15	2.909		2.809			
H_2O-HKUST-1(4th)	20	2.850		2.790			
H_2O-HKUST-1(5th)	30	3.017		2.780			
H_2O-HKUST-1(6th)	60	3.075		2.870			
H_2O-HKUST-1(7th)	120	3.598		2.953			
H_2O-HKUST-1(8th)	180	3.634	2.958	2.980	2.771	3.765	4.038
H_2O-HKUST-1(9th)	240	3.718	3.045	2.978	2.949	3.860	4.055

O[C], O[F]1, O[F]2 and O[F]3 are oxygen atoms in H_2O[C], H_2O[F]1, H_2O[F]2 and H_2O[F]3, respectively.

Supplementary Section 17. Changes in lattice parameters of HKUST-1.

An insightful reviewer asked us how the lattice parameter of an activated HKUST-1 can be changed during its water sorption. To address the question, we extracted powder X-ray diffraction (PXRD) patterns from the in situ SCXRD data set, which were measured with an activated HKUST-1 crystal, simultaneously exposing it to moist air. Then, we observed that the diffractions from (220) and (222) are most substantially influenced by the degree of moisture exposure. To confirm the extracted PXRD, we also measured PXRD patterns with a powder sample in the in situ manner. As shown in Supplementary Table 16 and Supplementary Figures 29-30 below, the unit cell length varied with respect to the moisture exposure. We expected the unit cell length to be linearly correlated or inversely correlated with the Cu–Cu length. However, our observation was different from our expectations. Initially, the unit cell length of the activated HKUST-1 increased as water molecules began to coordinate at the Cu^{2+} center. This point agreed well with our postulation. However, as the adsorbed water became considerable, the changes in the unit cell length did not show any trend. On the basis of this observation, we speculate that, when pore-filling water molecules begin to increase in the pores, the relation between the unit cell parameter and Cu–Cu length is more complex than what we expected, with many factors, such as interactions between different types of pore-filling water molecules and between the pore-filling water molecules and the organic components lining pores. Nonetheless, we think that the variation (26.243 – 26.399 Å; approximately 0.6% in length) is not enough to find the relationship between the lattice parameter and paddlewheel length.

Supplementary Table 17. Summary of lattice parameters of an Act-HKUST-1 crystal obtained from the in situ SCXRD (see the above Supplementary Tables 14 and 15).

Samples	Exposure time (min)	Unit cell (Å) a = b = c	Δa (Å)	Occupancy of water molecules (mol%)			
				$H_2O^{[C]a}$	$H_2O^{[F]1b}$	$H_2O^{[F]2b}$	$H_2O^{[F]3b}$
Act-HKUST-1	0	26.249(3)	–	0	0	0	0
H_2O-HKUST-1(1st)	5	26.275(3)	+0.026	10	0	0	0
H_2O-HKUST-1(2nd)	10	26.303(3)	+0.028	76	0	0	0
H_2O-HKUST-1(3rd)	15	26.282(3)	–0.021	96	16	0	0
H_2O-HKUST-1(4th)	20	26.296(3)	+0.014	100	20	0	0
H_2O-HKUST-1(5th)	30	26.243(3)	–0.053	100	28	0	0
H_2O-HKUST-1(6th)	60	26.245(3)	+0.002	100	34	0	0
H_2O-HKUST-1(7th)	120	26.265(3)	+0.020	100	56	0	0
H_2O-HKUST-1(8th)	180	26.393(3)	+0.128	100	56	40	24
H_2O-HKUST-1(9th)	240	26.399(3)	+0.006	100	86	50	18

[a]$H_2O^{[C]}$ is Cu^{2+}-coordinating H_2O.
[b]$H_2O^{[F]1}$, $H_2O^{[F]2}$ and $H_2O^{[F]3}$ are type-1, -2 and -3 pore-filling H_2O molecules, respectively.

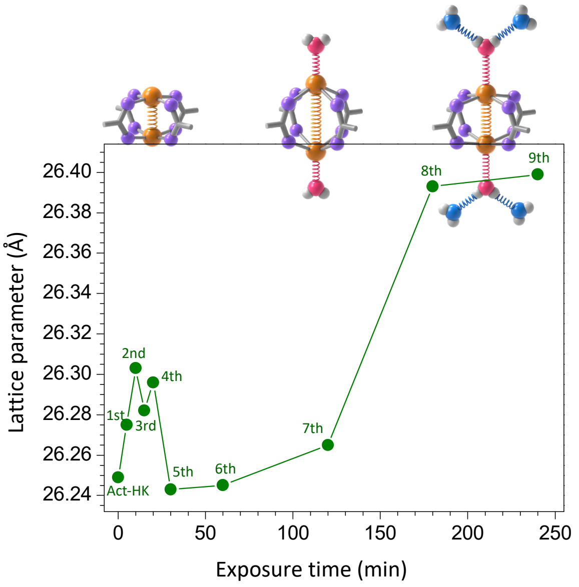

Supplementary Figure 29. Changes in the lattice parameters of an Act-HKUST-1 single crystal according to the moisture exposure at room temperature.

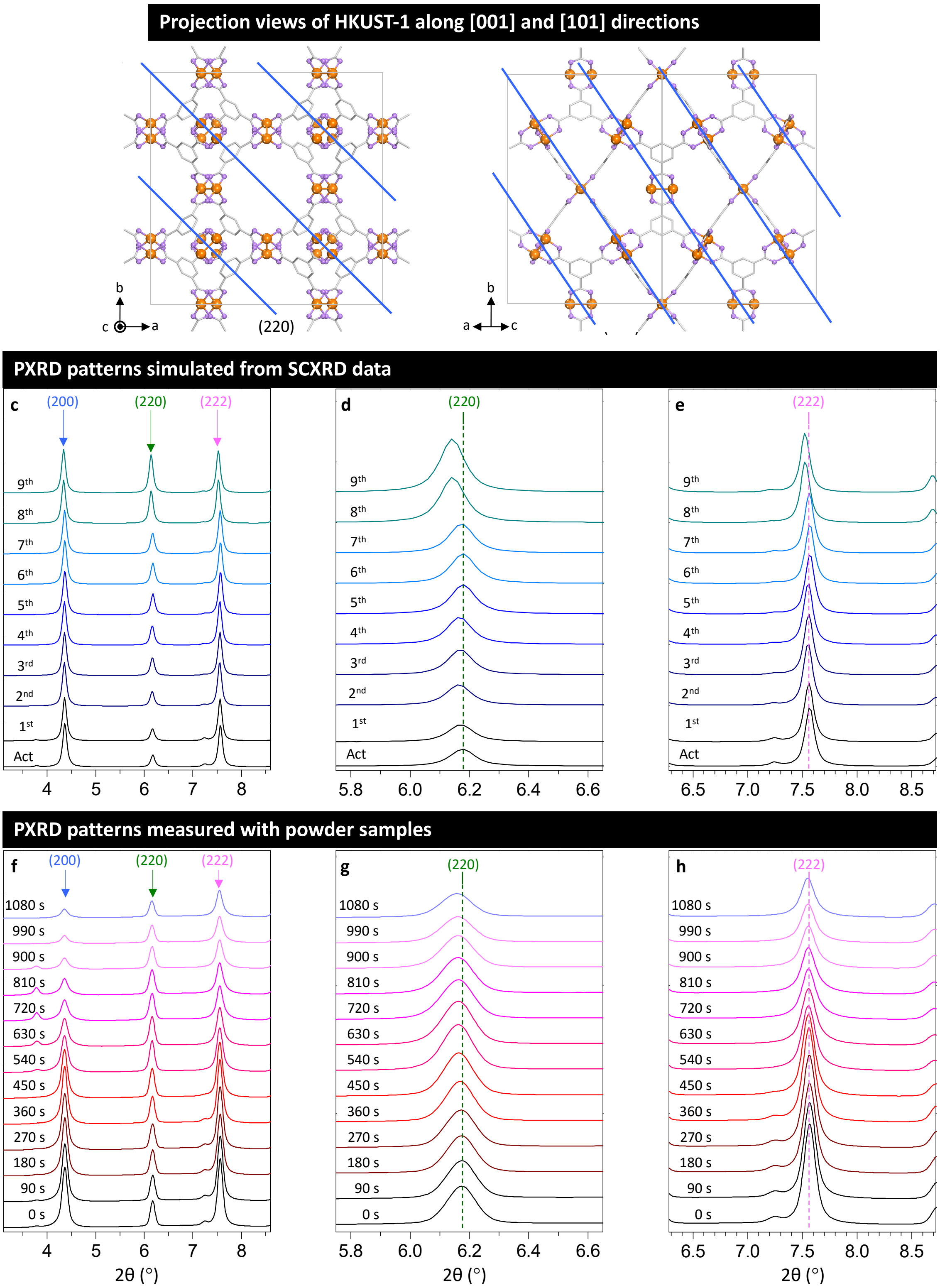

Supplementary Figure 30. (a-b) Projection images of HKUST-1 crystal viewed along (a) [001] and (b) [101] directions. (c) Wide and (d-e) expanded views of PXRD patterns of an Act-HKUST extracted from the in situ SCXRD data set, which were measured by exposing the crystal to moist air continuously. (c) Wide and (d-e) expanded views of in situ PXRD patterns of an activated HKUST-1 powder sample measured, continuously exposing it to moist air.

Supplementary Section 18. Water vapor sorption of HKUST-1.

Another insightful reviewer asked us to rationalize the water adsorption isotherm in light of water nucleation around preferred binding sites such as open-metal sites. To address this question, we measured water adsorption isotherm at 25 °C (see Supplementary Figure 31 below). From the isotherm, we found a few inflection points. We ascribe the inflection points to an influence from preferred water sites in pores and correspondingly classified (in this article) water types such as coordinating and type-1, -2, and -3 pore-filling water molecules ($H_2O^{[C]}$, $H_2O^{[F]1}$, $H_2O^{[F]2}$ and $H_2O^{[F]3}$, respectively)

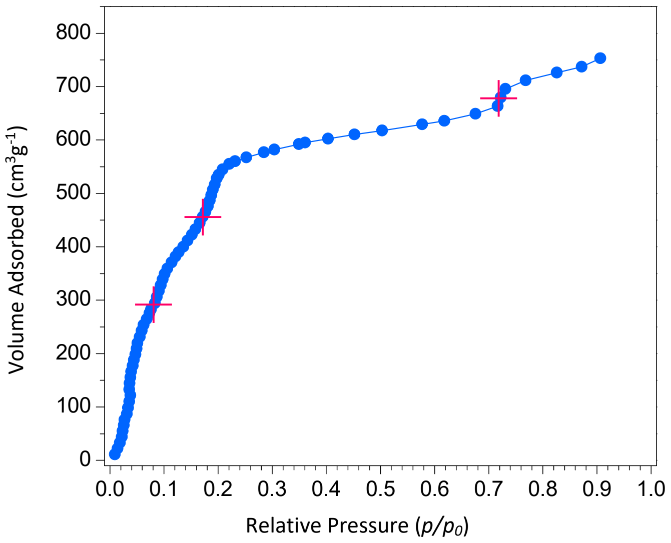

Supplementary Figure 31. Water vapor sorption isotherm of an activated HKUST-1 powder sample.

References

S1. Kim, H. K. et al. A Chemical Route to Activation of Open Metal Sites in the Copper-Based Metal–Organic Framework Materials HKUST-1 and Cu-MOF-2. *J. Am. Chem. Soc.* **137**, 10009-10015 (2015).

S2. Bae, J. et al. Multiple Coordination Exchanges for Room-Temperature Activation of Open-Metal Sites in Metal–Organic Frameworks. *ACS Appl. Mater. Interfaces* **9**, 24743-24752 (2017).

S3. Song, D. et al. Coordinative Reduction of Metal Nodes Enhances the Hydrolytic Stability of a Paddlewheel Metal–Organic Framework. *J. Am. Chem. Soc.* **141**, 7853-7864 (2019).

S4. Shin, J. W.; Eom, K.; Moon, D. BL2D-SMC, the Supramolecular Crystallography Beamline at the Pohang Light Source II, Korea. *J. Synchrotron Rad.* **23**, 369-373 (2016).

S5. Otwinowski, Z. & Minor, W. *in Methods in Enzymology*, **276**, page 307 in *Macromolecular Crystallography Part A* (eds Carter, Jr., C. & Sweet, R., Academic Press, New York, 1997)

S6. Sheldrick, G. M. SHELXT - Integrated space-group and crystal-structure determination. *Acta Cryst.* **A71**, 3–8 (2015).

S7. Sheldrick, G. M. Crystal structure refinement with SHELXL. *Acta Cryst.* **C71**, 3–8 (2015).

S8. Mammone, J. F., Sharma, S. K. & Nicol, M. Raman Spectra of Methanol and Ethanol at Pressures up to 100 Kbar. *J. Phys. Chem.* **84**, 3130-3134 (1980).

S9. Tan, K. et al., Defect Termination in the UiO-66 Family of Metal—Organic Frameworks: The Role of Water and Modulator. *J. Am. Chem. Soc.* **143**, 6328-6882 (2021).
