# Supplementary material for: Crystalline hydrogen bonding of water molecules confined in a metal-organic framework
Source: Commun Chem. 2022 Apr 8;5:51. doi: 10.1038/s42004-022-00666-8 (PMC9814150; doi:10.1038/s42004-022-00666-8)
Supplement: Supplementary file 3 — Supplementary Data 1 [file 42004_2022_666_MOESM3_ESM.zip › 220_Act-HK.rtf]

  Table 1.  Crystal data and structure refinement for Act-HK.
Identification code 	Act-HK
Empirical formula 	C18 H6 Cu3 O12
Formula weight 	604.85
Temperature 	220(2) K
Wavelength 	0.610 Å
Crystal system 	Cubic
Space group 	Fm-3m
Unit cell dimensions	a = 26.267(3) Å	a= 90°.
	b = 26.267(3) Å	b= 90°.
	c = 26.267(3) Å	g = 90°.
Volume	18123(6) Å3
Z	16
Density (calculated)	0.887 Mg/m3
Absorption coefficient	0.938 mm-1
F(000)	4752
Crystal size	0.125 x 0.117 x 0.085 mm3
Theta range for data collection	1.331 to 24.993°.
Index ranges	-36<=h<=36, -36<=k<=36, -36<=l<=36
Reflections collected	46582
Independent reflections	1320 [R(int) = 0.0997]
Completeness to theta = 21.469°	99.9 % 
Absorption correction	Empirical
Max. and min. transmission	1.000 and 0.956
Refinement method	Full-matrix least-squares on F2
Data / restraints / parameters	1320 / 0 / 33
Goodness-of-fit on F2	1.073
Final R indices [I>2sigma(I)]	R1 = 0.0273, wR2 = 0.0730
R indices (all data)	R1 = 0.0275, wR2 = 0.0731
Extinction coefficient	0.000191(18)
Largest diff. peak and hole	0.450 and -0.509 e.Å-3

 Table 2.  Atomic coordinates  ( x 104) and equivalent  isotropic displacement parameters (Å2x 103)
for Act-HK.  U(eq) is defined as one third of  the trace of the orthogonalized Uij tensor.
________________________________________________________________________________ 
	x	y	z	U(eq)
________________________________________________________________________________  
Cu(1)	7834(1)	2834(1)	5000	14(1)
O(1)	7438(1)	3167(1)	4477(1)	26(1)
C(1)	7036(1)	2964(1)	4310(1)	20(1)
C(2)	6786(1)	3214(1)	3863(1)	21(1)
C(3)	6353(1)	2998(1)	3647(1)	21(1)
________________________________________________________________________________ 
 Table 3.   Bond lengths [Å] and angles [°] for  Act-HK.
_____________________________________________________ 
Cu(1)-O(1) 	1.9320(7)
Cu(1)-O(1)#1 	1.9321(7)
Cu(1)-O(1)#2 	1.9321(7)
Cu(1)-O(1)#3 	1.9321(7)
Cu(1)-Cu(1)#4 	2.4849(5)
O(1)-C(1) 	1.2606(9)
C(1)-C(2) 	1.4974(18)
C(2)-C(3) 	1.3926(9)
C(2)-C(3)#5 	1.3926(9)
C(3)-H(3) 	0.9400

O(1)-Cu(1)-O(1)#1	88.94(5)
O(1)-Cu(1)-O(1)#2	172.87(4)
O(1)#1-Cu(1)-O(1)#2	90.61(5)
O(1)-Cu(1)-O(1)#3	90.61(5)
O(1)#1-Cu(1)-O(1)#3	172.87(4)
O(1)#2-Cu(1)-O(1)#3	88.94(5)
O(1)-Cu(1)-Cu(1)#4	86.43(2)
O(1)#1-Cu(1)-Cu(1)#4	86.43(2)
O(1)#2-Cu(1)-Cu(1)#4	86.43(2)
O(1)#3-Cu(1)-Cu(1)#4	86.43(2)
C(1)-O(1)-Cu(1)	120.56(7)
O(1)#6-C(1)-O(1)	125.82(12)
O(1)#6-C(1)-C(2)	117.08(6)
O(1)-C(1)-C(2)	117.08(6)
C(3)-C(2)-C(3)#5	120.16(13)
C(3)-C(2)-C(1)	119.87(7)
C(3)#5-C(2)-C(1)	119.87(7)
C(2)-C(3)-C(2)#7	119.84(13)
C(2)-C(3)-H(3)	120.1
C(2)#7-C(3)-H(3)	120.1
_____________________________________________________________ 
Symmetry transformations used to generate equivalent atoms: 
#1 y+1/2,x-1/2,z    #2 y+1/2,x-1/2,-z+1    #3 x,y,-z+1      
#4 -x+3/2,-y+1/2,-z+1    #5 -y+1,z,-x+1    #6 -y+1,-x+1,z      
#7 -z+1,-x+1,y      

 Table 4.   Anisotropic displacement parameters  (Å2x 103) for Act-HK.  The anisotropic
displacement factor exponent takes the form:  -2p2[ h2 a*2U11 + ...  + 2 h k a* b* U12 ]
______________________________________________________________________________ 
	U11	U22 	U33	U23	U13	U12
______________________________________________________________________________ 
Cu(1)	15(1) 	15(1)	12(1) 	0	0 	-6(1)
O(1)	27(1) 	27(1)	24(1) 	7(1)	-9(1) 	-7(1)
C(1)	23(1) 	23(1)	16(1) 	2(1)	-2(1) 	-1(1)
C(2)	22(1) 	22(1)	18(1) 	3(1)	-3(1) 	-3(1)
C(3)	21(1) 	22(1)	21(1) 	4(1)	-3(1) 	-4(1)
______________________________________________________________________________ 
 Table 5.   Hydrogen coordinates ( x 104) and isotropic  displacement parameters (Å2x 10 3)
for Act-HK.
________________________________________________________________________________ 
	x 	y 	z 	U(eq)
________________________________________________________________________________ 
 
H(3)	6206	2706	3794	26
________________________________________________________________________________ 
 Table 6.  Torsion angles [°] for Act-HK.
________________________________________________________________ 
Cu(1)-O(1)-C(1)-O(1)#6	5.3(2)
Cu(1)-O(1)-C(1)-C(2)	-172.88(8)
O(1)#6-C(1)-C(2)-C(3)	-0.88(19)
O(1)-C(1)-C(2)-C(3)	177.47(13)
O(1)#6-C(1)-C(2)-C(3)#5	-177.46(13)
O(1)-C(1)-C(2)-C(3)#5	0.89(19)
C(3)#5-C(2)-C(3)-C(2)#7	0.3(3)
C(1)-C(2)-C(3)-C(2)#7	-176.31(9)
________________________________________________________________ 
Symmetry transformations used to generate equivalent atoms: 
#1 y+1/2,x-1/2,z    #2 y+1/2,x-1/2,-z+1    #3 x,y,-z+1      
#4 -x+3/2,-y+1/2,-z+1    #5 -y+1,z,-x+1    #6 -y+1,-x+1,z      
#7 -z+1,-x+1,y      

 
 
