# Supplementary material for: Crystalline hydrogen bonding of water molecules confined in a metal-organic framework
Source: Commun Chem. 2022 Apr 8;5:51. doi: 10.1038/s42004-022-00666-8 (PMC9814150; doi:10.1038/s42004-022-00666-8)

checkCIF/PLATON report

You have not supplied any structure factors. As a result the full set of tests cannot be run.

THIS REPORT IS FOR GUIDANCE ONLY. IF USED AS PART OF A REVIEW PROCEDURE FOR PUBLICATION, IT SHOULD NOT REPLACE THE EXPERTISE OF AN EXPERIENCED CRYSTALLOGRAPHIC REFEREE.

No syntax errors found.   CIF dictionary   Interpreting this report

Datablock: H2OF-HK

Bond precision: C-C = 0.0043 A   Wavelength=0.61000

Cell:	a=26.275(3)	b=26.275(3)	c=26.275(3)
	alpha=90	beta=90	gamma=90
Temperature:	220 K		

	Calculated	Reported
Volume	18140(6)	18139(6)
Space group	F m -3 m	F m -3 m
Hall group	-F 4 2 3	-F 4 2 3
Moiety formula	C144 H96 Cu24 O120, 24(H2 O), 24(H2 O)	C18 H12 Cu3 O15 3(H2 O), 3(H2 O)
Sum formula	C144 H192 Cu24 O168	C18 H24 Cu3 O21
Mr	6136.18	766.99
Dx,g cm-3	1.123	1.123
Z	2	16
Mu (mm-1)	0.956	0.956
F000	6192.0	6192.0
F000’	6210.28	
h,k,lmax	36,36,36	36,36,36
Nref	1321	1315
Tmin,Tmax	0.806,0.822	0.868,1.000
Tmin’	0.806	

Correction method= # Reported T Limits: Tmin=0.868 Tmax=1.000
AbsCorr = EMPIRICAL

Data completeness= 0.995   Theta(max)= 24.985

R(reflections)= 0.0511(1121)   wR2(reflections)= 0.1621(1315)

S = 1.158   Npar= 59

The following ALERTS were generated. Each ALERT has the format
test-name_ALERT_alert-type_alert-level.
Click on the hyperlinks for more details of the test.

Alert level A

PLAT601_ALERT_2_A Unit Cell Contains Solvent Accessible VOIDS of . 866 Ang**3

Author Response: The several water molecule was found in the voids sapce. However, remained void space were still found in the crystal structure that did not contain resolved solvent molecules.

Alert level B

PLAT420_ALERT_2_B D-H Bond Without Acceptor O1W --H1WA . Please Check

Author Response: no available acceptors could be found at an acceptable distance from the water hydrogens

PLAT420_ALERT_2_B D-H Bond Without Acceptor O1W --H1WB . Please Check

Author Response: no available acceptors could be found at an acceptable distance from the water hydrogens

PLAT420_ALERT_2_B D-H Bond Without Acceptor O2W --H2WA . Please Check

Author Response: no available acceptors could be found at an acceptable distance from the water hydrogens

---

Alert level C

```
PLAT242_ALERT_2_C Low    'MainMol' Ueq as Compared to Neighbors of        Cu1 Check
PLAT260_ALERT_2_C Large Average Ueq of Residue Including      O2W      0.252 Check
PLAT260_ALERT_2_C Large Average Ueq of Residue Including      O1W      0.256 Check
```

Alert level G

```
FORMU01_ALERT_1_G  There is a discrepancy between the atom counts in the
            _chemical_formula_sum and _chemical_formula_moiety. This is
            usually due to the moiety formula being in the wrong format.
            Atom count from _chemical_formula_sum:   C18 H24 Cu3 O21
            Atom count from _chemical_formula_moiety:C18 H20 Cu3 O157
ABSMU01_ALERT_1_G  Calculation of _exptl_absorpt_correction_mu
                not performed for this radiation type.
PLAT002_ALERT_2_G Number of Distance or Angle Restraints on AtSite          8 Note
PLAT004_ALERT_5_G Polymeric Structure Found with Maximum Dimension          2 Info
PLAT013_ALERT_1_G N.O.K.   _shelx_hkl_checksum Found in CIF ......     Please Check
PLAT019_ALERT_1_G _diffrn_measured_fraction_theta_full/*_max < 1.0      0.998 Report
PLAT042_ALERT_1_G Calc. and Reported Moiety Formula Strings Differ     Please Check
PLAT045_ALERT_1_G Calculated and Reported Z Differ by a Factor ...       0.13 Check
PLAT083_ALERT_2_G SHELXL Second Parameter in WGHT  Unusually Large      29.65 Why ?
PLAT092_ALERT_4_G Check: Wavelength Given is not Cu,Ga,Mo,Ag,In Ka    0.61000 Ang.
```

```
PLAT172_ALERT_4_G The CIF-Embedded .res File Contains DFIX Records          3 Report
PLAT173_ALERT_4_G The CIF-Embedded .res File Contains DANG Records          3 Report
PLAT300_ALERT_4_G Atom Site Occupancy of O2W       Constrained at         0.5 Check
PLAT300_ALERT_4_G Atom Site Occupancy of H2WA      Constrained at         0.5 Check
PLAT300_ALERT_4_G Atom Site Occupancy of H2WB      Constrained at        0.25 Check
PLAT300_ALERT_4_G Atom Site Occupancy of O1W       Constrained at         0.5 Check
PLAT300_ALERT_4_G Atom Site Occupancy of H1WA      Constrained at         0.5 Check
PLAT300_ALERT_4_G Atom Site Occupancy of H1WB      Constrained at         0.5 Check
PLAT302_ALERT_4_G Anion/Solvent/Minor-Residue Disorder (Resd  2  )       100% Note
PLAT302_ALERT_4_G Anion/Solvent/Minor-Residue Disorder (Resd  3  )       100% Note
PLAT720_ALERT_4_G Number of Unusual/Non-Standard Labels ..........          5 Note
PLAT764_ALERT_4_G Overcomplete CIF Bond List Detected (Rep/Expd) .       1.11 Ratio
PLAT794_ALERT_5_G Tentative Bond Valency for Cu1       (II)      .       2.17 Info
PLAT860_ALERT_3_G Number of Least-Squares Restraints .............          9 Note
PLAT883_ALERT_1_G No Info/Value for _atom_sites_solution_primary .     Please Do !
PLAT965_ALERT_2_G The SHELXL WEIGHT Optimisation has not Converged     Please Check
```

```
   1 ALERT level A = Most likely a serious problem - resolve or explain
   3 ALERT level B = A potentially serious problem, consider carefully
   3 ALERT level C = Check. Ensure it is not caused by an omission or oversight
  26 ALERT level G = General information/check it is not something unexpected

   7 ALERT type 1 CIF construction/syntax error, inconsistent or missing data
  10 ALERT type 2 Indicator that the structure model may be wrong or deficient
   1 ALERT type 3 Indicator that the structure quality may be low
  13 ALERT type 4 Improvement, methodology, query or suggestion
   2 ALERT type 5 Informative message, check
```

It is advisable to attempt to resolve as many as possible of the alerts in all categories. Often the minor alerts point to easily fixed oversights, errors and omissions in your CIF or refinement strategy, so attention to these fine details can be worthwhile. In order to resolve some of the more serious problems it may be necessary to carry out additional measurements or structure refinements. However, the purpose of your study may justify the reported deviations and the more serious of these should normally be commented upon in the discussion or experimental section of a paper or in the "special_details" fields of the CIF. checkCIF was carefully designed to identify outliers and unusual parameters, but every test has its limitations and alerts that are not important in a particular case may appear. Conversely, the absence of alerts does not guarantee there are no aspects of the results needing attention. It is up to the individual to critically assess their own results and, if necessary, seek expert advice.

Publication of your CIF in IUCr journals

A basic structural check has been run on your CIF. These basic checks will be run on all CIFs submitted for publication in IUCr journals (*Acta Crystallographica*, *Journal of Applied Crystallography*, *Journal of Synchrotron Radiation*); however, if you intend to submit to *Acta Crystallographica Section C* or *E* or *IUCrData*, you should make sure that full publication checks are run on the final version of your CIF prior to submission.

Publication of your CIF in other journals

Please refer to the *Notes for Authors* of the relevant journal for any special instructions relating to CIF submission.

PLATON version of 03/06/2021; check.def file version of 02/06/2021

Datablock H2OF-HK - ellipsoid plot

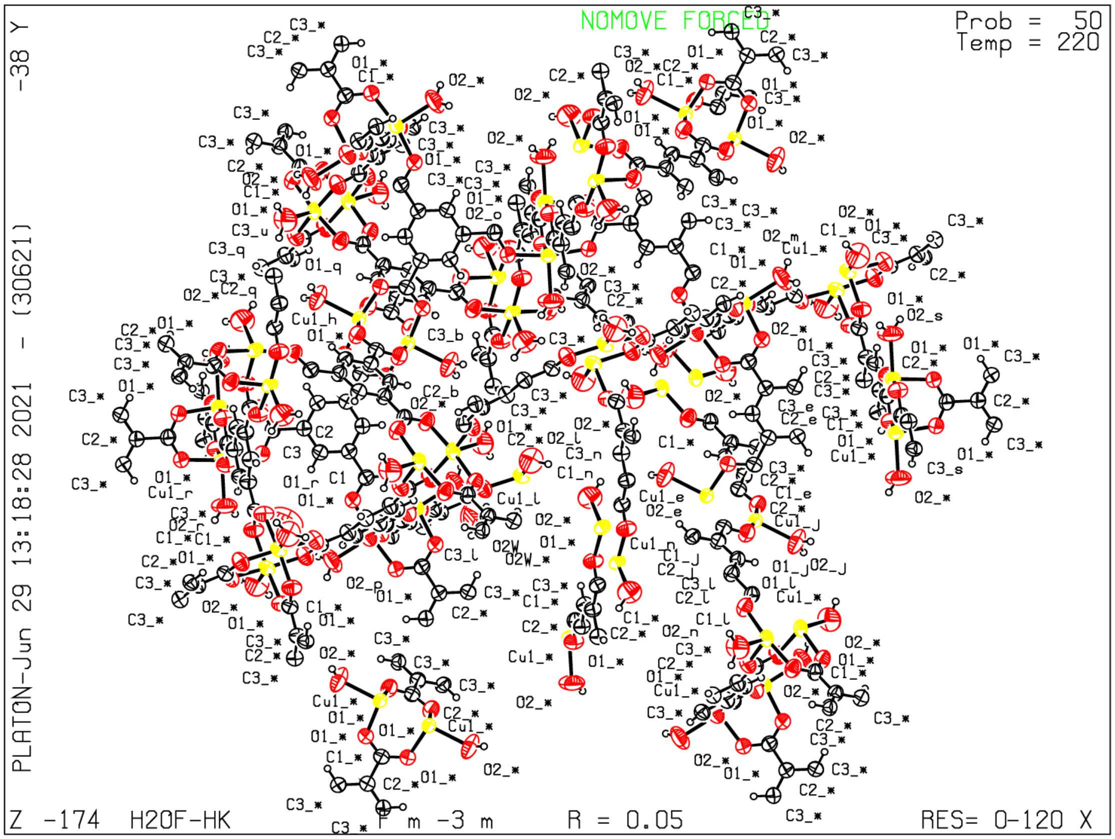

Supplement: Supplementary file 3 — Supplementary Data 1 [file 42004_2022_666_MOESM3_ESM.zip › 220_H2O[F]-HK.pdf]
