# Supplementary material for: Crystalline hydrogen bonding of water molecules confined in a metal-organic framework
Source: Commun Chem. 2022 Apr 8;5:51. doi: 10.1038/s42004-022-00666-8 (PMC9814150; doi:10.1038/s42004-022-00666-8)
Supplement: Supplementary file 3 — Supplementary Data 1 [file 42004_2022_666_MOESM3_ESM.zip › 220_H2O[F]-HK.rtf]

  Table 1.  Crystal data and structure refinement for H2O[F]-HK.
Identification code 	H2O[F]-HK
Empirical formula 	C18 H24 Cu3 O21
Formula weight 	766.99
Temperature 	220(2) K
Wavelength 	0.610 Å
Crystal system 	Cubic
Space group 	Fm-3m
Unit cell dimensions	a = 26.275(3) Å	a= 90°.
	b = 26.275(3) Å	b= 90°.
	c = 26.275(3) Å	g = 90°.
Volume	18139(6) Å3
Z	16
Density (calculated)	1.123 Mg/m3
Absorption coefficient	0.956 mm-1
F(000)	6192
Crystal size	0.225 x 0.215 x 0.205 mm3
Theta range for data collection	2.206 to 24.985°.
Index ranges	-36<=h<=36, -36<=k<=36, -36<=l<=36
Reflections collected	45312
Independent reflections	1315 [R(int) = 0.0917]
Completeness to theta = 21.469°	99.3 % 
Absorption correction	Empirical
Max. and min. transmission	1.000 and 0.868
Refinement method	Full-matrix least-squares on F2
Data / restraints / parameters	1315 / 9 / 59
Goodness-of-fit on F2	1.158
Final R indices [I>2sigma(I)]	R1 = 0.0511, wR2 = 0.1528
R indices (all data)	R1 = 0.0580, wR2 = 0.1621
Extinction coefficient	n/a
Largest diff. peak and hole	0.638 and -0.362 e.Å-3

 Table 2.  Atomic coordinates  ( x 104) and equivalent  isotropic displacement parameters (Å2x 103)
for H2O[F]-HK.  U(eq) is defined as one third of  the trace of the orthogonalized Uij tensor.
________________________________________________________________________________ 
	x	y	z	U(eq)
________________________________________________________________________________  
Cu(1)	2148(1)	5000	2852(1)	52(1)
O(1)	2566(1)	5524(1)	3170(1)	68(1)
O(2)	1557(1)	5000	3443(1)	116(2)
C(1)	2966(1)	5687(1)	2966(1)	58(1)
C(2)	3217(1)	6134(1)	3217(1)	59(1)
C(3)	3650(1)	6350(1)	3003(1)	59(1)
O(1W)	3963(5)	6037(5)	1680(5)	256(10)
O(2W)	746(5)	5000	2737(6)	252(10)
________________________________________________________________________________ 
 Table 3.   Bond lengths [Å] and angles [°] for  H2O[F]-HK.
_____________________________________________________ 
Cu(1)-O(1) 	1.950(2)
Cu(1)-O(1)#1 	1.9499(19)
Cu(1)-O(1)#2 	1.9499(19)
Cu(1)-O(1)#3 	1.9499(19)
Cu(1)-O(2) 	2.199(5)
Cu(1)-Cu(1)#4 	2.6131(11)
O(1)-C(1) 	1.255(3)
O(2)-H(1O2) 	0.950(7)
O(2)-H(1O2)#2 	0.950(7)
C(1)-C(2) 	1.501(5)
C(2)-C(3) 	1.389(3)
C(2)-C(3)#5 	1.389(3)
C(3)-H(3) 	0.9400
O(1W)-H(1WA) 	0.9500(10)
O(1W)-H(1WB) 	0.9500(10)
O(2W)-H(2WA) 	0.9500(10)
O(2W)-H(2WB) 	0.9500(10)

O(1)-Cu(1)-O(1)#1	169.12(12)
O(1)-Cu(1)-O(1)#2	89.04(13)
O(1)#1-Cu(1)-O(1)#2	89.93(13)
O(1)-Cu(1)-O(1)#3	89.93(13)
O(1)#1-Cu(1)-O(1)#3	89.04(13)
O(1)#2-Cu(1)-O(1)#3	169.12(12)
O(1)-Cu(1)-O(2)	95.44(6)
O(1)#1-Cu(1)-O(2)	95.44(6)
O(1)#2-Cu(1)-O(2)	95.44(6)
O(1)#3-Cu(1)-O(2)	95.44(6)
O(1)-Cu(1)-Cu(1)#4	84.56(6)
O(1)#1-Cu(1)-Cu(1)#4	84.56(6)
O(1)#2-Cu(1)-Cu(1)#4	84.56(6)
O(1)#3-Cu(1)-Cu(1)#4	84.56(6)
O(2)-Cu(1)-Cu(1)#4	180.00(3)
C(1)-O(1)-Cu(1)	122.0(2)
Cu(1)-O(2)-H(1O2)	125.7(12)
Cu(1)-O(2)-H(1O2)#2	125.7(12)
H(1O2)-O(2)-H(1O2)#2	109(2)
O(1)#6-C(1)-O(1)	126.8(4)
O(1)#6-C(1)-C(2)	116.59(18)
O(1)-C(1)-C(2)	116.59(18)
C(3)-C(2)-C(3)#5	119.7(4)
C(3)-C(2)-C(1)	120.11(18)
C(3)#5-C(2)-C(1)	120.11(19)
C(2)#7-C(3)-C(2)	120.3(4)
C(2)#7-C(3)-H(3)	119.9
C(2)-C(3)-H(3)	119.9
H(1WA)-O(1W)-H(1WB)	109(2)
H(2WA)-O(2W)-H(2WB)	109(2)
_____________________________________________________________ 
Symmetry transformations used to generate equivalent atoms: 
#1 -z+1/2,-y+1,-x+1/2    #2 -z+1/2,y,-x+1/2    #3 x,-y+1,z      
#4 -x+1/2,-y+1,-z+1/2    #5 z,-x+1,-y+1    #6 z,y,x      
#7 -y+1,-z+1,x      

 Table 4.   Anisotropic displacement parameters  (Å2x 103) for H2O[F]-HK.  The anisotropic
displacement factor exponent takes the form:  -2p2[ h2 a*2U11 + ...  + 2 h k a* b* U12 ]
______________________________________________________________________________ 
	U11	U22 	U33	U23	U13	U12
______________________________________________________________________________ 
Cu(1)	55(1) 	45(1)	55(1) 	0	13(1) 	0
O(1)	71(1) 	64(1)	69(1) 	-14(1)	14(1) 	-13(1)
O(2)	89(2) 	172(6)	89(2) 	0	42(3) 	0
C(1)	63(1) 	49(2)	63(1) 	-1(1)	2(2) 	-1(1)
C(2)	61(1) 	56(2)	61(1) 	-4(1)	4(2) 	-4(1)
C(3)	59(1) 	59(1)	59(2) 	-5(1)	5(1) 	1(2)
O(1W)	323(16) 	323(16)	122(10) 	-18(9)	18(9) 	0(20)
O(2W)	111(9) 	380(30)	265(17) 	0	5(12) 	0
______________________________________________________________________________ 
 Table 5.   Hydrogen coordinates ( x 104) and isotropic  displacement parameters (Å2x 10 3)
for H2O[F]-HK.
________________________________________________________________________________ 
	x 	y 	z 	U(eq)
________________________________________________________________________________ 
 
H(1O2)	1200(4)	5000	3385(7)	140
H(3)	3797	6203	2712	71
H(1WA)	4181(7)	5819(7)	1492(16)	384
H(1WB)	3765(8)	6235(8)	1451(14)	384
H(2WA)	650(30)	5000	2387(7)	379
H(2WB)	483(16)	5160(30)	2930(19)	379
________________________________________________________________________________ 
 Table 6.  Torsion angles [°] for H2O[F]-HK.
________________________________________________________________ 
Cu(1)-O(1)-C(1)-O(1)#6	-5.1(6)
Cu(1)-O(1)-C(1)-C(2)	172.7(2)
O(1)#6-C(1)-C(2)-C(3)	0.7(6)
O(1)-C(1)-C(2)-C(3)	-177.3(4)
O(1)#6-C(1)-C(2)-C(3)#5	177.3(4)
O(1)-C(1)-C(2)-C(3)#5	-0.7(6)
C(3)#5-C(2)-C(3)-C(2)#7	-0.5(8)
C(1)-C(2)-C(3)-C(2)#7	176.1(3)
________________________________________________________________ 
Symmetry transformations used to generate equivalent atoms: 
#1 -z+1/2,-y+1,-x+1/2    #2 -z+1/2,y,-x+1/2    #3 x,-y+1,z      
#4 -x+1/2,-y+1,-z+1/2    #5 z,-x+1,-y+1    #6 z,y,x      
#7 -y+1,-z+1,x      

 Table 7.  Hydrogen bonds for H2O[F]-HK  [Å and °].
____________________________________________________________________________ 
D-H...A	d(D-H)	d(H...A)	d(D...A)	<(DHA)
____________________________________________________________________________ 
 O(2)-H(1O2)...O(2W^a)	0.950(7)	2.08(2)	2.825(16)	134.3(16)
 O(1W)-H(1WB)...O(1W)#8	0.9500(10)	1.68(3)	2.39(3)	128.4(16)
 O(1W)-H(1WB)...O(1W)#9	0.9500(10)	1.68(3)	2.39(3)	128.4(16)
 O(2W^a)-H(2WB^a)...O(2W^a)#10	0.9500(10)	2.07(6)	2.77(2)	130(6)
____________________________________________________________________________ 
Symmetry transformations used to generate equivalent atoms: 
#1 -z+1/2,-y+1,-x+1/2    #2 -z+1/2,y,-x+1/2    #3 x,-y+1,z      
#4 -x+1/2,-y+1,-z+1/2    #5 z,-x+1,-y+1    #6 z,y,x      
#7 -y+1,-z+1,x    #8 -z+1/2,-x+1,y-1/2    #9 -y+1,z+1/2,-x+1/2      
#10 y-1/2,x+1/2,z      

 
 
