# Supplementary material for: Crystalline hydrogen bonding of water molecules confined in a metal-organic framework
Source: Commun Chem. 2022 Apr 8;5:51. doi: 10.1038/s42004-022-00666-8 (PMC9814150; doi:10.1038/s42004-022-00666-8)
Supplement: Supplementary file 3 — Supplementary Data 1 [file 42004_2022_666_MOESM3_ESM.zip › 298_Act-HK.rtf]

  Table 1.  Crystal data and structure refinement for Act-HK.
Identification code 	Act-HK
Empirical formula 	C18 H6 Cu3 O12
Formula weight 	604.85
Temperature 	298(2) K
Wavelength 	0.630 Å
Crystal system 	Cubic
Space group 	Fm-3m
Unit cell dimensions	a = 26.249(3) Å	a= 90°.
	b = 26.249(3) Å	b= 90°.
	c = 26.249(3) Å	g = 90°.
Volume	18086(6) Å3
Z	16
Density (calculated)	0.889 Mg/m3
Absorption coefficient	1.027 mm-1
F(000)	4752
Crystal size	0.054 x 0.052 x 0.050 mm3
Theta range for data collection	1.191 to 25.975°.
Index ranges	-36<=h<=36, -36<=k<=36, -36<=l<=36
Reflections collected	46344
Independent reflections	1323 [R(int) = 0.1856]
Completeness to theta = 22.210°	98.9 % 
Absorption correction	Empirical
Max. and min. transmission	1.000 and 0.824
Refinement method	Full-matrix least-squares on F2
Data / restraints / parameters	1323 / 0 / 32
Goodness-of-fit on F2	1.031
Final R indices [I>2sigma(I)]	R1 = 0.0548, wR2 = 0.1565
R indices (all data)	R1 = 0.0817, wR2 = 0.1707
Extinction coefficient	n/a
Largest diff. peak and hole	0.499 and -0.493 e.Å-3

 Table 2.  Atomic coordinates  ( x 104) and equivalent  isotropic displacement parameters (Å2x 103)
for Act-HK.  U(eq) is defined as one third of  the trace of the orthogonalized Uij tensor.
________________________________________________________________________________ 
	x	y	z	U(eq)
________________________________________________________________________________  
Cu(1)	2165(1)	2835(1)	5000	70(1)
O(1)	2564(1)	3166(1)	5523(1)	83(1)
C(1)	2965(1)	2965(1)	5691(2)	74(1)
C(2)	3212(1)	3212(1)	6140(2)	76(1)
C(3)	3644(1)	3001(2)	6356(1)	78(1)
________________________________________________________________________________ 
 Table 3.   Bond lengths [Å] and angles [°] for  Act-HK.
_____________________________________________________ 
Cu(1)-O(1) 	1.932(2)
Cu(1)-O(1)#1 	1.932(2)
Cu(1)-O(1)#2 	1.932(2)
Cu(1)-O(1)#3 	1.932(2)
Cu(1)-Cu(1)#4 	2.4842(14)
O(1)-C(1) 	1.256(3)
C(1)-C(2) 	1.494(6)
C(2)-C(3)#5 	1.384(3)
C(2)-C(3) 	1.384(3)
C(3)-H(3) 	0.9300

O(1)-Cu(1)-O(1)#1	172.53(13)
O(1)-Cu(1)-O(1)#2	90.51(14)
O(1)#1-Cu(1)-O(1)#2	89.01(14)
O(1)-Cu(1)-O(1)#3	89.01(14)
O(1)#1-Cu(1)-O(1)#3	90.50(14)
O(1)#2-Cu(1)-O(1)#3	172.53(13)
O(1)-Cu(1)-Cu(1)#4	86.26(7)
O(1)#1-Cu(1)-Cu(1)#4	86.26(7)
O(1)#2-Cu(1)-Cu(1)#4	86.26(7)
O(1)#3-Cu(1)-Cu(1)#4	86.26(7)
C(1)-O(1)-Cu(1)	121.0(2)
O(1)#6-C(1)-O(1)	125.4(4)
O(1)#6-C(1)-C(2)	117.3(2)
O(1)-C(1)-C(2)	117.3(2)
C(3)#5-C(2)-C(3)	119.3(4)
C(3)#5-C(2)-C(1)	120.3(2)
C(3)-C(2)-C(1)	120.3(2)
C(2)#7-C(3)-C(2)	120.6(4)
C(2)#7-C(3)-H(3)	119.7
C(2)-C(3)-H(3)	119.7
_____________________________________________________________ 
Symmetry transformations used to generate equivalent atoms: 
#1 -y+1/2,-x+1/2,-z+1    #2 x,y,-z+1    #3 -y+1/2,-x+1/2,z      
#4 -x+1/2,-y+1/2,-z+1    #5 y,-z+1,-x+1    #6 y,x,z      
#7 -z+1,x,-y+1      

 Table 4.   Anisotropic displacement parameters  (Å2x 103) for Act-HK.  The anisotropic
displacement factor exponent takes the form:  -2p2[ h2 a*2U11 + ...  + 2 h k a* b* U12 ]
______________________________________________________________________________ 
	U11	U22 	U33	U23	U13	U12
______________________________________________________________________________ 
Cu(1)	71(1) 	71(1)	67(1) 	0	0 	9(1)
O(1)	86(1) 	83(1)	81(1) 	-12(1)	-12(1) 	14(1)
C(1)	76(2) 	76(2)	69(2) 	1(1)	1(1) 	0(2)
C(2)	76(2) 	76(2)	77(2) 	-4(2)	-4(2) 	6(2)
C(3)	80(2) 	74(2)	80(2) 	-3(2)	0(2) 	3(2)
______________________________________________________________________________ 
 Table 5.   Hydrogen coordinates ( x 104) and isotropic  displacement parameters (Å2x 10 3)
for Act-HK.
________________________________________________________________________________ 
	x 	y 	z 	U(eq)
________________________________________________________________________________ 
 
H(3)	3791	2713	6209	93
________________________________________________________________________________ 
 Table 6.  Torsion angles [°] for Act-HK.
________________________________________________________________ 
Cu(1)-O(1)-C(1)-O(1)#6	5.4(6)
Cu(1)-O(1)-C(1)-C(2)	-172.3(3)
O(1)#6-C(1)-C(2)-C(3)#5	-177.7(4)
O(1)-C(1)-C(2)-C(3)#5	0.2(6)
O(1)#6-C(1)-C(2)-C(3)	-0.2(6)
O(1)-C(1)-C(2)-C(3)	177.7(4)
C(3)#5-C(2)-C(3)-C(2)#7	0.9(9)
C(1)-C(2)-C(3)-C(2)#7	-176.6(3)
________________________________________________________________ 
Symmetry transformations used to generate equivalent atoms: 
#1 -y+1/2,-x+1/2,-z+1    #2 x,y,-z+1    #3 -y+1/2,-x+1/2,z      
#4 -x+1/2,-y+1/2,-z+1    #5 y,-z+1,-x+1    #6 y,x,z      
#7 -z+1,x,-y+1      

 
 
