# Supplementary material for: Crystalline hydrogen bonding of water molecules confined in a metal-organic framework
Source: Commun Chem. 2022 Apr 8;5:51. doi: 10.1038/s42004-022-00666-8 (PMC9814150; doi:10.1038/s42004-022-00666-8)
Supplement: Supplementary file 3 — Supplementary Data 1 [file 42004_2022_666_MOESM3_ESM.zip › 298_H2O-HK(1st).rtf]

  Table 8.  Crystal data and structure refinement for H2O-HK(1st).
Identification code 	H2O-HK(1st)
Empirical formula 	C18 H6.60 Cu3 O12.30
Formula weight 	610.25
Temperature 	298(2) K
Wavelength 	0.630 Å
Crystal system 	Cubic
Space group 	Fm-3m
Unit cell dimensions	a = 26.275(3) Å	a= 90°.
	b = 26.275(3) Å	b= 90°.
	c = 26.275(3) Å	g = 90°.
Volume	18139(6) Å3
Z	16
Density (calculated)	0.894 Mg/m3
Absorption coefficient	1.025 mm-1
F(000)	4800
Crystal size	0.054 x 0.052 x 0.050 mm3
Theta range for data collection	2.749 to 25.989°.
Index ranges	-36<=h<=36, -36<=k<=36, -36<=l<=36
Reflections collected	46856
Independent reflections	1329 [R(int) = 0.1930]
Completeness to theta = 22.210°	99.2 % 
Absorption correction	Empirical
Max. and min. transmission	1.000 and 0.786
Refinement method	Full-matrix least-squares on F2
Data / restraints / parameters	1329 / 9 / 38
Goodness-of-fit on F2	1.052
Final R indices [I>2sigma(I)]	R1 = 0.0638, wR2 = 0.1795
R indices (all data)	R1 = 0.0956, wR2 = 0.1979
Extinction coefficient	n/a
Largest diff. peak and hole	0.559 and -0.331 e.Å-3

 Table 9.  Atomic coordinates  ( x 104) and equivalent  isotropic displacement parameters (Å2x 103)
for H2O-HK(1st).  U(eq) is defined as one third of  the trace of the orthogonalized Uij tensor.
________________________________________________________________________________ 
	x	y	z	U(eq)
________________________________________________________________________________  
Cu(1)	2163(1)	2837(1)	5000	72(1)
O(1)	2567(1)	3165(1)	5524(1)	85(1)
C(1)	2963(1)	2963(1)	5693(2)	75(1)
C(2)	3214(1)	3214(1)	6140(2)	77(1)
C(3)	3646(1)	3004(2)	6354(1)	79(1)
O(1W)	1547(7)	3453(7)	5000	60(8)
________________________________________________________________________________ 
 Table 10.   Bond lengths [Å] and angles [°] for  H2O-HK(1st).
_____________________________________________________ 
Cu(1)-O(1)#1 	1.940(2)
Cu(1)-O(1)#2 	1.940(2)
Cu(1)-O(1)#3 	1.940(2)
Cu(1)-O(1) 	1.940(2)
Cu(1)-O(1W) 	2.29(3)
Cu(1)-Cu(1)#4 	2.5050(16)
O(1)-C(1) 	1.249(3)
C(1)-C(2) 	1.501(7)
C(2)-C(3)#5 	1.381(3)
C(2)-C(3) 	1.381(3)
C(3)-H(3) 	0.9300
O(1W)-H(1O1) 	0.919(7)
O(1W)-H(1O1)#1 	0.919(7)

O(1)#1-Cu(1)-O(1)#2	90.35(16)
O(1)#1-Cu(1)-O(1)#3	89.03(16)
O(1)#2-Cu(1)-O(1)#3	171.60(15)
O(1)#1-Cu(1)-O(1)	171.60(15)
O(1)#2-Cu(1)-O(1)	89.03(16)
O(1)#3-Cu(1)-O(1)	90.35(16)
O(1)#1-Cu(1)-O(1W)	94.20(8)
O(1)#2-Cu(1)-O(1W)	94.20(8)
O(1)#3-Cu(1)-O(1W)	94.20(8)
O(1)-Cu(1)-O(1W)	94.20(8)
O(1)#1-Cu(1)-Cu(1)#4	85.80(8)
O(1)#2-Cu(1)-Cu(1)#4	85.80(8)
O(1)#3-Cu(1)-Cu(1)#4	85.80(8)
O(1)-Cu(1)-Cu(1)#4	85.80(8)
O(1W)-Cu(1)-Cu(1)#4	180.00(4)
C(1)-O(1)-Cu(1)	121.3(3)
O(1)-C(1)-O(1)#6	125.5(5)
O(1)-C(1)-C(2)	117.3(2)
O(1)#6-C(1)-C(2)	117.3(2)
C(3)#5-C(2)-C(3)	119.4(5)
C(3)#5-C(2)-C(1)	120.3(2)
C(3)-C(2)-C(1)	120.3(2)
C(2)#7-C(3)-C(2)	120.5(5)
C(2)#7-C(3)-H(3)	119.7
C(2)-C(3)-H(3)	119.7
Cu(1)-O(1W)-H(1O1)	124.1(13)
Cu(1)-O(1W)-H(1O1)#1	124.1(13)
H(1O1)-O(1W)-H(1O1)#1	112(3)
_____________________________________________________________ 
Symmetry transformations used to generate equivalent atoms: 
#1 -y+1/2,-x+1/2,-z+1    #2 -y+1/2,-x+1/2,z    #3 x,y,-z+1      
#4 -x+1/2,-y+1/2,-z+1    #5 y,-z+1,-x+1    #6 y,x,z      
#7 -z+1,x,-y+1      

 Table 11.   Anisotropic displacement parameters  (Å2x 103) for H2O-HK(1st).  The anisotropic
displacement factor exponent takes the form:  -2p2[ h2 a*2U11 + ...  + 2 h k a* b* U12 ]
______________________________________________________________________________ 
	U11	U22 	U33	U23	U13	U12
______________________________________________________________________________ 
Cu(1)	74(1) 	74(1)	67(1) 	0	0 	7(1)
O(1)	88(2) 	84(2)	82(2) 	-10(1)	-12(1) 	12(1)
C(1)	77(2) 	77(2)	70(3) 	0(2)	0(2) 	2(2)
C(2)	77(2) 	77(2)	76(3) 	-3(2)	-3(2) 	3(2)
C(3)	80(2) 	76(3)	80(2) 	-4(2)	-1(2) 	4(2)
O(1W)	54(9) 	54(9)	72(16) 	0	0 	35(12)
______________________________________________________________________________ 
 Table 12.   Hydrogen coordinates ( x 104) and isotropic  displacement parameters (Å2x 10 3)
for H2O-HK(1st).
________________________________________________________________________________ 
	x 	y 	z 	U(eq)
________________________________________________________________________________ 
 
H(3)	3795	2719	6205	95
H(1O1)	1203(8)	3387(10)	5000	90
________________________________________________________________________________ 
 Table 13.  Torsion angles [°] for H2O-HK(1st).
________________________________________________________________ 
Cu(1)-O(1)-C(1)-O(1)#6	6.6(7)
Cu(1)-O(1)-C(1)-C(2)	-172.7(3)
O(1)-C(1)-C(2)-C(3)#5	0.8(7)
O(1)#6-C(1)-C(2)-C(3)#5	-178.5(4)
O(1)-C(1)-C(2)-C(3)	178.5(4)
O(1)#6-C(1)-C(2)-C(3)	-0.8(7)
C(3)#5-C(2)-C(3)-C(2)#7	2.0(11)
C(1)-C(2)-C(3)-C(2)#7	-175.7(3)
________________________________________________________________ 
Symmetry transformations used to generate equivalent atoms: 
#1 -y+1/2,-x+1/2,-z+1    #2 -y+1/2,-x+1/2,z    #3 x,y,-z+1      
#4 -x+1/2,-y+1/2,-z+1    #5 y,-z+1,-x+1    #6 y,x,z      
#7 -z+1,x,-y+1      

 
 
