# Supplementary material for: Crystalline hydrogen bonding of water molecules confined in a metal-organic framework
Source: Commun Chem. 2022 Apr 8;5:51. doi: 10.1038/s42004-022-00666-8 (PMC9814150; doi:10.1038/s42004-022-00666-8)
Supplement: Supplementary file 3 — Supplementary Data 1 [file 42004_2022_666_MOESM3_ESM.zip › 298_H2O-HK(3rd).rtf]

  Table 1.  Crystal data and structure refinement for H2O-HK(3rd).
Identification code 	H2O-HK(3rd)
Empirical formula 	C18 H13.68 Cu3 O15.84
Formula weight 	674.03
Temperature 	298(2) K
Wavelength 	0.630 Å
Crystal system 	Cubic
Space group 	Fm-3m
Unit cell dimensions	a = 26.282(3) Å	a= 90°.
	b = 26.282(3) Å	b= 90°.
	c = 26.282(3) Å	g = 90°.
Volume	18155(6) Å3
Z	16
Density (calculated)	0.986 Mg/m3
Absorption coefficient	1.032 mm-1
F(000)	5366
Crystal size	0.054 x 0.052 x 0.050 mm3
Theta range for data collection	2.748 to 25.981°.
Index ranges	-36<=h<=36, -36<=k<=36, -36<=l<=36
Reflections collected	46830
Independent reflections	1324 [R(int) = 0.2113]
Completeness to theta = 22.210°	98.6 % 
Absorption correction	Empirical
Max. and min. transmission	1.000 and 0.787
Refinement method	Full-matrix least-squares on F2
Data / restraints / parameters	1324 / 18 / 53
Goodness-of-fit on F2	0.996
Final R indices [I>2sigma(I)]	R1 = 0.0699, wR2 = 0.2200
R indices (all data)	R1 = 0.1176, wR2 = 0.2522
Extinction coefficient	n/a
Largest diff. peak and hole	0.553 and -0.379 e.Å-3

 Table 2.  Atomic coordinates  ( x 104) and equivalent  isotropic displacement parameters (Å2x 103)
for H2O-HK(3rd).  U(eq) is defined as one third of  the trace of the orthogonalized Uij tensor.
________________________________________________________________________________ 
	x	y	z	U(eq)
________________________________________________________________________________  
Cu(1)	2150(1)	2850(1)	5000	78(1)
O(1)	2567(1)	3171(1)	5523(1)	95(1)
C(1)	2961(2)	2961(2)	5689(3)	84(2)
C(2)	3213(2)	3213(2)	6146(2)	88(2)
C(3)	3642(2)	3004(3)	6358(2)	89(2)
O(1W)	1562(2)	3438(2)	5000	149(4)
O(2W)	746(15)	2700(9)	5121(11)	114(14)
________________________________________________________________________________ 
 Table 3.   Bond lengths [Å] and angles [°] for  H2O-HK(3rd).
_____________________________________________________ 
Cu(1)-O(1)#1 	1.950(3)
Cu(1)-O(1)#2 	1.950(3)
Cu(1)-O(1)#3 	1.950(3)
Cu(1)-O(1) 	1.950(3)
Cu(1)-O(1W) 	2.184(8)
Cu(1)-Cu(1)#4 	2.605(2)
O(1)-C(1) 	1.251(4)
C(1)-C(2) 	1.524(10)
C(2)-C(3)#5 	1.372(5)
C(2)-C(3) 	1.372(5)
C(3)-H(3) 	0.9300
O(1W)-H(1O1) 	0.918(7)
O(1W)-H(1O1)#1 	0.918(7)
O(2W)-H(1O2) 	0.9200(11)
O(2W)-H(2O2) 	0.9200(11)

O(1)#1-Cu(1)-O(1)#2	89.7(2)
O(1)#1-Cu(1)-O(1)#3	89.3(2)
O(1)#2-Cu(1)-O(1)#3	169.4(2)
O(1)#1-Cu(1)-O(1)	169.4(2)
O(1)#2-Cu(1)-O(1)	89.3(2)
O(1)#3-Cu(1)-O(1)	89.7(2)
O(1)#1-Cu(1)-O(1W)	95.31(10)
O(1)#2-Cu(1)-O(1W)	95.31(10)
O(1)#3-Cu(1)-O(1W)	95.31(10)
O(1)-Cu(1)-O(1W)	95.31(10)
O(1)#1-Cu(1)-Cu(1)#4	84.69(10)
O(1)#2-Cu(1)-Cu(1)#4	84.69(10)
O(1)#3-Cu(1)-Cu(1)#4	84.69(10)
O(1)-Cu(1)-Cu(1)#4	84.69(10)
O(1W)-Cu(1)-Cu(1)#4	180.00(5)
C(1)-O(1)-Cu(1)	121.4(4)
O(1)-C(1)-O(1)#6	127.5(7)
O(1)-C(1)-C(2)	116.3(3)
O(1)#6-C(1)-C(2)	116.3(3)
C(3)#5-C(2)-C(3)	119.6(7)
C(3)#5-C(2)-C(1)	120.2(3)
C(3)-C(2)-C(1)	120.2(3)
C(2)#7-C(3)-C(2)	120.4(7)
C(2)#7-C(3)-H(3)	119.8
C(2)-C(3)-H(3)	119.8
Cu(1)-O(1W)-H(1O1)	123.7(13)
Cu(1)-O(1W)-H(1O1)#1	123.7(13)
H(1O1)-O(1W)-H(1O1)#1	113(3)
H(1O2)-O(2W)-H(2O2)	111.40(18)
_____________________________________________________________ 
Symmetry transformations used to generate equivalent atoms: 
#1 -y+1/2,-x+1/2,-z+1    #2 -y+1/2,-x+1/2,z    #3 x,y,-z+1      
#4 -x+1/2,-y+1/2,-z+1    #5 y,-z+1,-x+1    #6 y,x,z      
#7 -z+1,x,-y+1      

 Table 4.   Anisotropic displacement parameters  (Å2x 103) for H2O-HK(3rd).  The anisotropic
displacement factor exponent takes the form:  -2p2[ h2 a*2U11 + ...  + 2 h k a* b* U12 ]
______________________________________________________________________________ 
	U11	U22 	U33	U23	U13	U12
______________________________________________________________________________ 
Cu(1)	81(1) 	81(1)	73(1) 	0	0 	13(1)
O(1)	95(2) 	94(2)	94(2) 	-15(2)	-18(2) 	19(2)
C(1)	88(2) 	88(2)	77(4) 	0(2)	0(2) 	-2(3)
C(2)	92(3) 	92(3)	79(4) 	-7(2)	-7(2) 	6(3)
C(3)	89(2) 	90(4)	89(2) 	-8(2)	-9(3) 	8(2)
O(1W)	132(4) 	132(4)	184(9) 	0	0 	52(6)
O(2W)	94(16) 	140(20)	110(20) 	-5(16)	-5(16) 	6(15)
______________________________________________________________________________ 
 Table 5.   Hydrogen coordinates ( x 104) and isotropic  displacement parameters (Å2x 10 3)
for H2O-HK(3rd).
________________________________________________________________________________ 
	x 	y 	z 	U(eq)
________________________________________________________________________________ 
 
H(3)	3789	2718	6211	107
H(1O1)	1219(4)	3369(8)	5000	224
H(1O2)	780(40)	2429(10)	4904(16)	171
H(2O2)	640(30)	2986(9)	4950(20)	171
________________________________________________________________________________ 
 Table 6.  Torsion angles [°] for H2O-HK(3rd).
________________________________________________________________ 
Cu(1)-O(1)-C(1)-O(1)#6	7.6(10)
Cu(1)-O(1)-C(1)-C(2)	-171.9(4)
O(1)-C(1)-C(2)-C(3)#5	0.9(10)
O(1)#6-C(1)-C(2)-C(3)#5	-178.7(6)
O(1)-C(1)-C(2)-C(3)	178.7(6)
O(1)#6-C(1)-C(2)-C(3)	-0.9(10)
C(3)#5-C(2)-C(3)-C(2)#7	1.5(16)
C(1)-C(2)-C(3)-C(2)#7	-176.4(5)
________________________________________________________________ 
Symmetry transformations used to generate equivalent atoms: 
#1 -y+1/2,-x+1/2,-z+1    #2 -y+1/2,-x+1/2,z    #3 x,y,-z+1      
#4 -x+1/2,-y+1/2,-z+1    #5 y,-z+1,-x+1    #6 y,x,z      
#7 -z+1,x,-y+1      

 Table 7.  Hydrogen bonds for H2O-HK(3rd)  [Å and °].
____________________________________________________________________________ 
D-H...A	d(D-H)	d(H...A)	d(D...A)	<(DHA)
____________________________________________________________________________ 
 O(1W)-H(1O1)...O(2W^a)	0.918(7)	2.18(3)	2.91(4)	136(2)
 O(1W)-H(1O1)...O(2W^a)#3	0.918(7)	2.18(3)	2.91(4)	136(2)
____________________________________________________________________________ 
Symmetry transformations used to generate equivalent atoms: 
#1 -y+1/2,-x+1/2,-z+1    #2 -y+1/2,-x+1/2,z    #3 x,y,-z+1      
#4 -x+1/2,-y+1/2,-z+1    #5 y,-z+1,-x+1    #6 y,x,z      
#7 -z+1,x,-y+1      

 
 
