# Supplementary material for: Crystalline hydrogen bonding of water molecules confined in a metal-organic framework
Source: Commun Chem. 2022 Apr 8;5:51. doi: 10.1038/s42004-022-00666-8 (PMC9814150; doi:10.1038/s42004-022-00666-8)
Supplement: Supplementary file 3 — Supplementary Data 1 [file 42004_2022_666_MOESM3_ESM.zip › 298_H2O-HK(4th).rtf]

  Table 1.  Crystal data and structure refinement for H2O-HK(4th).
Identification code 	H2O-HK(4th)
Empirical formula 	C18 H14.40 Cu3 O16.20
Formula weight 	680.51
Temperature 	298(2) K
Wavelength 	0.630 Å
Crystal system 	Cubic
Space group 	Fm-3m
Unit cell dimensions	a = 26.296(3) Å	a= 90°.
	b = 26.296(3) Å	b= 90°.
	c = 26.296(3) Å	g = 90°.
Volume	18184(6) Å3
Z	16
Density (calculated)	0.994 Mg/m3
Absorption coefficient	1.031 mm-1
F(000)	5424
Crystal size	0.054 x 0.052 x 0.050 mm3
Theta range for data collection	1.373 to 25.998°.
Index ranges	-36<=h<=36, -36<=k<=36, -36<=l<=36
Reflections collected	46990
Independent reflections	1326 [R(int) = 0.2499]
Completeness to theta = 22.210°	98.4 % 
Absorption correction	Empirical
Max. and min. transmission	1.000 and 0.836
Refinement method	Full-matrix least-squares on F2
Data / restraints / parameters	1326 / 18 / 53
Goodness-of-fit on F2	0.941
Final R indices [I>2sigma(I)]	R1 = 0.0841, wR2 = 0.2496
R indices (all data)	R1 = 0.1469, wR2 = 0.2930
Extinction coefficient	n/a
Largest diff. peak and hole	0.612 and -0.435 e.Å-3

 Table 2.  Atomic coordinates  ( x 104) and equivalent  isotropic displacement parameters (Å2x 103)
for H2O-HK(4th).  U(eq) is defined as one third of  the trace of the orthogonalized Uij tensor.
________________________________________________________________________________ 
	x	y	z	U(eq)
________________________________________________________________________________  
Cu(1)	2148(1)	2852(1)	5000	84(1)
O(1)	2570(2)	3172(2)	5525(2)	100(1)
C(1)	2963(2)	2963(2)	5690(3)	90(2)
C(2)	3220(2)	3220(2)	6139(3)	97(2)
C(3)	3646(2)	3007(3)	6354(2)	99(2)
O(1W)	1558(2)	3442(2)	5000	145(4)
O(2W)	739(14)	2744(9)	5129(10)	111(13)
________________________________________________________________________________ 
 Table 3.   Bond lengths [Å] and angles [°] for  H2O-HK(4th).
_____________________________________________________ 
Cu(1)-O(1)#1 	1.962(4)
Cu(1)-O(1)#2 	1.962(4)
Cu(1)-O(1)#3 	1.962(4)
Cu(1)-O(1) 	1.962(4)
Cu(1)-O(1W) 	2.192(9)
Cu(1)-Cu(1)#4 	2.621(3)
O(1)-C(1) 	1.247(5)
C(1)-C(2) 	1.519(12)
C(2)-C(3)#5 	1.375(6)
C(2)-C(3) 	1.375(6)
C(3)-H(3) 	0.9300
O(1W)-H(1O1) 	0.919(7)
O(1W)-H(1O1)#1 	0.919(7)
O(2W)-H(1O2) 	0.9200(11)
O(2W)-H(2O2) 	0.9200(12)

O(1)#1-Cu(1)-O(1)#2	89.5(2)
O(1)#1-Cu(1)-O(1)#3	89.4(3)
O(1)#2-Cu(1)-O(1)#3	168.8(2)
O(1)#1-Cu(1)-O(1)	168.8(2)
O(1)#2-Cu(1)-O(1)	89.4(3)
O(1)#3-Cu(1)-O(1)	89.5(2)
O(1)#1-Cu(1)-O(1W)	95.62(12)
O(1)#2-Cu(1)-O(1W)	95.62(12)
O(1)#3-Cu(1)-O(1W)	95.62(12)
O(1)-Cu(1)-O(1W)	95.62(12)
O(1)#1-Cu(1)-Cu(1)#4	84.38(12)
O(1)#2-Cu(1)-Cu(1)#4	84.38(12)
O(1)#3-Cu(1)-Cu(1)#4	84.38(12)
O(1)-Cu(1)-Cu(1)#4	84.38(12)
O(1W)-Cu(1)-Cu(1)#4	180.00(6)
C(1)-O(1)-Cu(1)	121.7(5)
O(1)-C(1)-O(1)#6	127.5(8)
O(1)-C(1)-C(2)	116.2(4)
O(1)#6-C(1)-C(2)	116.2(4)
C(3)#5-C(2)-C(3)	119.7(9)
C(3)#5-C(2)-C(1)	120.1(4)
C(3)-C(2)-C(1)	120.1(4)
C(2)-C(3)-C(2)#7	120.3(9)
C(2)-C(3)-H(3)	119.8
C(2)#7-C(3)-H(3)	119.8
Cu(1)-O(1W)-H(1O1)	124.1(13)
Cu(1)-O(1W)-H(1O1)#1	124.1(13)
H(1O1)-O(1W)-H(1O1)#1	112(3)
H(1O2)-O(2W)-H(2O2)	111.40(18)
_____________________________________________________________ 
Symmetry transformations used to generate equivalent atoms: 
#1 -y+1/2,-x+1/2,-z+1    #2 -y+1/2,-x+1/2,z    #3 x,y,-z+1      
#4 -x+1/2,-y+1/2,-z+1    #5 y,-z+1,-x+1    #6 y,x,z      
#7 -z+1,x,-y+1      

 Table 4.   Anisotropic displacement parameters  (Å2x 103) for H2O-HK(4th).  The anisotropic
displacement factor exponent takes the form:  -2p2[ h2 a*2U11 + ...  + 2 h k a* b* U12 ]
______________________________________________________________________________ 
	U11	U22 	U33	U23	U13	U12
______________________________________________________________________________ 
Cu(1)	87(1) 	87(1)	78(1) 	0	0 	14(1)
O(1)	105(3) 	97(3)	99(3) 	-16(2)	-18(2) 	20(2)
C(1)	92(3) 	92(3)	86(5) 	2(3)	2(3) 	3(4)
C(2)	101(3) 	101(3)	90(5) 	-5(3)	-5(3) 	11(4)
C(3)	96(3) 	104(5)	96(3) 	-8(3)	-6(4) 	8(3)
O(1W)	133(5) 	133(5)	168(8) 	0	0 	50(6)
O(2W)	90(15) 	144(19)	100(20) 	12(15)	-4(14) 	0(15)
______________________________________________________________________________ 
 Table 5.   Hydrogen coordinates ( x 104) and isotropic  displacement parameters (Å2x 10 3)
for H2O-HK(4th).
________________________________________________________________________________ 
	x 	y 	z 	U(eq)
________________________________________________________________________________ 
 
H(3)	3790	2718	6210	118
H(1O1)	1215(4)	3375(8)	5000	217
H(1O2)	820(40)	2488(10)	4904(17)	166
H(2O2)	710(50)	3052(9)	4967(18)	166
________________________________________________________________________________ 
 Table 6.  Torsion angles [°] for H2O-HK(4th).
________________________________________________________________ 
Cu(1)-O(1)-C(1)-O(1)#6	7.7(12)
Cu(1)-O(1)-C(1)-C(2)	-172.9(5)
O(1)-C(1)-C(2)-C(3)#5	2.5(13)
O(1)#6-C(1)-C(2)-C(3)#5	-178.0(8)
O(1)-C(1)-C(2)-C(3)	178.1(8)
O(1)#6-C(1)-C(2)-C(3)	-2.5(13)
C(3)#5-C(2)-C(3)-C(2)#7	-0.3(19)
C(1)-C(2)-C(3)-C(2)#7	-175.8(6)
________________________________________________________________ 
Symmetry transformations used to generate equivalent atoms: 
#1 -y+1/2,-x+1/2,-z+1    #2 -y+1/2,-x+1/2,z    #3 x,y,-z+1      
#4 -x+1/2,-y+1/2,-z+1    #5 y,-z+1,-x+1    #6 y,x,z      
#7 -z+1,x,-y+1      

 Table 7.  Hydrogen bonds for H2O-HK(4th)  [Å and °].
____________________________________________________________________________ 
D-H...A	d(D-H)	d(H...A)	d(D...A)	<(DHA)
____________________________________________________________________________ 
 O(1W)-H(1O1)...O(2W^a)	0.919(7)	2.11(3)	2.85(3)	137.1(19)
 O(1W)-H(1O1)...O(2W^a)#3	0.919(7)	2.11(3)	2.85(3)	137.1(19)
____________________________________________________________________________ 
Symmetry transformations used to generate equivalent atoms: 
#1 -y+1/2,-x+1/2,-z+1    #2 -y+1/2,-x+1/2,z    #3 x,y,-z+1      
#4 -x+1/2,-y+1/2,-z+1    #5 y,-z+1,-x+1    #6 y,x,z      
#7 -z+1,x,-y+1      

 
 
