# Supplementary material for: Crystalline hydrogen bonding of water molecules confined in a metal-organic framework
Source: Commun Chem. 2022 Apr 8;5:51. doi: 10.1038/s42004-022-00666-8 (PMC9814150; doi:10.1038/s42004-022-00666-8)
Supplement: Supplementary file 3 — Supplementary Data 1 [file 42004_2022_666_MOESM3_ESM.zip › 298_H2O-HK(7th).rtf]

  Table 1.  Crystal data and structure refinement for H2O-HK(7th).
Identification code 	H2O-HK(7th)
Empirical formula 	C18 H18.72 Cu3 O18.36
Formula weight 	719.43
Temperature 	298(2) K
Wavelength 	0.630 Å
Crystal system 	Cubic
Space group 	Fm-3m
Unit cell dimensions	a = 26.265(3) Å	a= 90°.
	b = 26.265(3) Å	b= 90°.
	c = 26.265(3) Å	g = 90°.
Volume	18118(6) Å3
Z	16
Density (calculated)	1.055 Mg/m3
Absorption coefficient	1.039 mm-1
F(000)	5770
Crystal size	0.054 x 0.052 x 0.050 mm3
Theta range for data collection	1.190 to 26.000°.
Index ranges	-36<=h<=36, -36<=k<=36, -36<=l<=36
Reflections collected	46041
Independent reflections	1319 [R(int) = 0.3929]
Completeness to theta = 22.210°	98.5 % 
Absorption correction	Empirical
Max. and min. transmission	1.000 and 0.834
Refinement method	Full-matrix least-squares on F2
Data / restraints / parameters	1319 / 19 / 47
Goodness-of-fit on F2	1.014
Final R indices [I>2sigma(I)]	R1 = 0.1144, wR2 = 0.3254
R indices (all data)	R1 = 0.2286, wR2 = 0.3950
Extinction coefficient	n/a
Largest diff. peak and hole	0.662 and -0.323 e.Å-3

 Table 2.  Atomic coordinates  ( x 104) and equivalent  isotropic displacement parameters (Å2x 103)
for H2O-HK(7th).  U(eq) is defined as one third of  the trace of the orthogonalized Uij tensor.
________________________________________________________________________________ 
	x	y	z	U(eq)
________________________________________________________________________________  
Cu(1)	2147(1)	2853(1)	5000	104(1)
O(1)	2567(2)	3172(2)	5518(3)	122(2)
C(1)	2965(3)	2965(3)	5696(4)	110(4)
C(2)	3224(3)	3224(3)	6140(4)	110(4)
C(3)	3633(4)	3005(5)	6367(4)	114(4)
O(1W)	1572(4)	3428(4)	5000	169(6)
O(2W)	795(5)	2300(6)	5000(70)	260(13)
________________________________________________________________________________ 
 Table 3.   Bond lengths [Å] and angles [°] for  H2O-HK(7th).
_____________________________________________________ 
Cu(1)-O(1)#1 	1.940(7)
Cu(1)-O(1) 	1.941(7)
Cu(1)-O(1)#2 	1.941(7)
Cu(1)-O(1)#3 	1.941(7)
Cu(1)-O(1W) 	2.136(16)
Cu(1)-Cu(1)#4 	2.621(4)
O(1)-C(1) 	1.268(7)
C(1)-C(2) 	1.5102(10)
C(2)-C(3) 	1.358(8)
C(2)-C(3)#5 	1.358(8)
C(3)-H(3) 	0.9300
O(1W)-H(1O1) 	0.919(7)
O(1W)-H(1O1)#2 	0.919(7)
O(2W)-H(1O2) 	0.920(2)
O(2W)-H(2O2) 	0.920(3)

O(1)#1-Cu(1)-O(1)	89.0(4)
O(1)#1-Cu(1)-O(1)#2	90.0(4)
O(1)-Cu(1)-O(1)#2	168.9(4)
O(1)#1-Cu(1)-O(1)#3	168.9(4)
O(1)-Cu(1)-O(1)#3	90.0(4)
O(1)#2-Cu(1)-O(1)#3	89.0(4)
O(1)#1-Cu(1)-O(1W)	95.54(18)
O(1)-Cu(1)-O(1W)	95.54(18)
O(1)#2-Cu(1)-O(1W)	95.54(18)
O(1)#3-Cu(1)-O(1W)	95.54(18)
O(1)#1-Cu(1)-Cu(1)#4	84.46(18)
O(1)-Cu(1)-Cu(1)#4	84.46(18)
O(1)#2-Cu(1)-Cu(1)#4	84.46(18)
O(1)#3-Cu(1)-Cu(1)#4	84.46(18)
O(1W)-Cu(1)-Cu(1)#4	180.0(4)
C(1)-O(1)-Cu(1)	122.8(6)
O(1)-C(1)-O(1)#6	124.8(10)
O(1)-C(1)-C(2)	117.6(5)
O(1)#6-C(1)-C(2)	117.6(5)
C(3)-C(2)-C(3)#5	118.5(13)
C(3)-C(2)-C(1)	120.4(6)
C(3)#5-C(2)-C(1)	120.4(6)
C(2)-C(3)-C(2)#7	121.2(13)
C(2)-C(3)-H(3)	119.4
C(2)#7-C(3)-H(3)	119.4
Cu(1)-O(1W)-H(1O1)	124.0(13)
Cu(1)-O(1W)-H(1O1)#2	124.0(13)
H(1O1)-O(1W)-H(1O1)#2	112(3)
H(1O2)-O(2W)-H(2O2)	112.4(6)
_____________________________________________________________ 
Symmetry transformations used to generate equivalent atoms: 
#1 x,y,-z+1    #2 -y+1/2,-x+1/2,-z+1    #3 -y+1/2,-x+1/2,z      
#4 -x+1/2,-y+1/2,-z+1    #5 y,-z+1,-x+1    #6 y,x,z      
#7 -z+1,x,-y+1      

 Table 4.   Anisotropic displacement parameters  (Å2x 103) for H2O-HK(7th).  The anisotropic
displacement factor exponent takes the form:  -2p2[ h2 a*2U11 + ...  + 2 h k a* b* U12 ]
______________________________________________________________________________ 
	U11	U22 	U33	U23	U13	U12
______________________________________________________________________________ 
Cu(1)	106(1) 	106(1)	101(2) 	0	0 	11(1)
O(1)	122(5) 	112(5)	131(5) 	-6(4)	-19(4) 	20(4)
C(1)	109(6) 	109(6)	110(9) 	4(5)	4(5) 	-12(8)
C(2)	116(6) 	116(6)	99(8) 	-8(5)	-8(5) 	-3(8)
C(3)	115(6) 	114(9)	115(6) 	-3(6)	-26(8) 	3(6)
O(1W)	157(7) 	157(7)	193(12) 	0	0 	32(9)
O(2W)	199(17) 	340(20)	245(19) 	0(20)	0(20) 	-19(16)
______________________________________________________________________________ 
 Table 5.   Hydrogen coordinates ( x 104) and isotropic  displacement parameters (Å2x 10 3)
for H2O-HK(7th).
________________________________________________________________________________ 
	x 	y 	z 	U(eq)
________________________________________________________________________________ 
 
H(3)	3760	2700	6240	137
H(1O1)	1228(5)	3361(8)	5000	254
H(1O2)	889	1963	5018	391
H(2O2)	1070	2514	4959	391
________________________________________________________________________________ 
 Table 6.  Torsion angles [°] for H2O-HK(7th).
________________________________________________________________ 
Cu(1)-O(1)-C(1)-O(1)#6	9.4(18)
Cu(1)-O(1)-C(1)-C(2)	-173.0(7)
O(1)-C(1)-C(2)-C(3)	175.9(11)
O(1)#6-C(1)-C(2)-C(3)	-6.3(19)
O(1)-C(1)-C(2)-C(3)#5	6.3(19)
O(1)#6-C(1)-C(2)-C(3)#5	-175.9(11)
C(3)#5-C(2)-C(3)-C(2)#7	-8(3)
C(1)-C(2)-C(3)-C(2)#7	-177.9(9)
________________________________________________________________ 
Symmetry transformations used to generate equivalent atoms: 
#1 x,y,-z+1    #2 -y+1/2,-x+1/2,-z+1    #3 -y+1/2,-x+1/2,z      
#4 -x+1/2,-y+1/2,-z+1    #5 y,-z+1,-x+1    #6 y,x,z      
#7 -z+1,x,-y+1      

 Table 7.  Hydrogen bonds for H2O-HK(7th)  [Å and °].
____________________________________________________________________________ 
D-H...A	d(D-H)	d(H...A)	d(D...A)	<(DHA)
____________________________________________________________________________ 
 O(2W^a)-H(2O2^a)...O(1)#3	0.920(3)	2.483(7)	3.05(10)	120(9)
 O(2W^a)-H(2O2^a)...O(1)#2	0.920(3)	2.363(7)	3.06(7)	132(7)
____________________________________________________________________________ 
Symmetry transformations used to generate equivalent atoms: 
#1 x,y,-z+1    #2 -y+1/2,-x+1/2,-z+1    #3 -y+1/2,-x+1/2,z      
#4 -x+1/2,-y+1/2,-z+1    #5 y,-z+1,-x+1    #6 y,x,z      
#7 -z+1,x,-y+1      

 
 
