# Supplementary material for: Crystalline hydrogen bonding of water molecules confined in a metal-organic framework
Source: Commun Chem. 2022 Apr 8;5:51. doi: 10.1038/s42004-022-00666-8 (PMC9814150; doi:10.1038/s42004-022-00666-8)

checkCIF/PLATON report

You have not supplied any structure factors. As a result the full set of tests cannot be run.

THIS REPORT IS FOR GUIDANCE ONLY. IF USED AS PART OF A REVIEW PROCEDURE FOR PUBLICATION, IT SHOULD NOT REPLACE THE EXPERTISE OF AN EXPERIENCED CRYSTALLOGRAPHIC REFEREE.

No syntax errors found. CIF dictionary Interpreting this report

Datablock: H2O-HK8th

```
Bond precision:  C-C = 0.0127 A          Wavelength=0.63000

Cell:              a=26.393(3)      b=26.393(3)      c=26.393(3)
                   alpha=90         beta=90          gamma=90
Temperature:       298 K

                Calculated                  Reported
Volume          18385(6)                    18385(6)
Space group     F m -3 m                    F m -3 m
Hall group      -F 4 2 3                    -F 4 2 3
                2(C3 H2 Cu0.50 O2.50),      C18 H6 Cu3 O12 3(H2 O),
Moiety formula  5.12(H0.08 O0.04), 4(H0.40  3.36(H2 O), 0.48(H2 O),
                O0.20)                      2.4(H2 O)
Sum formula     C6 H8.16 Cu O7.08           C18 H24.48 Cu3 O21.24
Mr              257.12                      771.31
Dx,g cm-3       1.115                       1.115
Z               48                          16
Mu (mm-1)       1.030                       1.030
F000            6230.4                      6230.0
F000'           6249.07
h,k,lmax        36,36,36                    36,36,36
Nref            1348                        1334
Tmin,Tmax       0.946,0.950                 0.815,1.000
Tmin'           0.946

Correction method= # Reported T Limits: Tmin=0.815 Tmax=1.000
AbsCorr = EMPIRICAL

Data completeness= 0.990          Theta(max)= 25.989

R(reflections)= 0.1070( 470)      wR2(reflections)= 0.3769( 1334)

S = 1.035                   Npar= 65
```

The following ALERTS were generated. Each ALERT has the format
test-name_ALERT_alert-type_alert-level.
Click on the hyperlinks for more details of the test.

Alert level A

RINTA01_ALERT_3_A The value of Rint is greater than 0.25
Rint given 0.352

Author Response: The pristine crystal is going to continuously exposure under the small amount of water mist, it cause as the gradually lost crystallinity. However the structure was determined and refined properly.

PLAT020_ALERT_3_A The Value of Rint is Greater Than 0.12 .... 0.352 Report

Author Response: The pristine crystal is going to continuously exposure under the small amount of water mist, it cause as the gradually lost crystallinity. However the structure was determined and refined properly.

PLAT601_ALERT_2_A Unit Cell Contains Solvent Accessible VOIDS of . 285 Ang**3

Author Response: This crystal have exposured into very small amount of water mist. It has observed that the unit cell contains large accesible voids in the crystal during the sturucture analysis. The structure solvent of water molecle have almolst occupied in the voids, but it has still got a solvent accesible sites.

Alert level B

PLAT026_ALERT_3_B Ratio Observed / Unique Reflections (too) Low .. 35% Check

Author Response: The crystal of limited quality due to exposure under exposure water mist does not diffract X-ray well and the numbers of observed reflections are reduced than pristine crystal.

PLAT084_ALERT_3_B High wR2 Value (i.e. > 0.25) .... 0.38 Report

Author Response: wR2 is large because the quality of the diffraction data is sub-optimal. However, the refinements converge well and yield a completely reasonable structure.

Alert level C

```
PLAT082_ALERT_2_C High R1 Value ..................................      0.11 Report
PLAT241_ALERT_2_C High   'MainMol' Ueq as Compared to Neighbors of        O1 Check
PLAT242_ALERT_2_C Low    'MainMol' Ueq as Compared to Neighbors of       Cu1 Check
PLAT260_ALERT_2_C Large Average Ueq of Residue Including       Cu1     0.110 Check
PLAT260_ALERT_2_C Large Average Ueq of Residue Including       O2W     0.192 Check
PLAT260_ALERT_2_C Large Average Ueq of Residue Including       O3W     0.200 Check
PLAT260_ALERT_2_C Large Average Ueq of Residue Including       O4W     0.243 Check
```

```
PLAT341_ALERT_3_C Low Bond Precision on  C-C Bonds ..............    0.01267 Ang.
```

Alert level G

```
FORMU01_ALERT_1_G  There is a discrepancy between the atom counts in the
            _chemical_formula_sum and _chemical_formula_moiety. This is
            usually due to the moiety formula being in the wrong format.
            Atom count from _chemical_formula_sum:   C18 H24.48 Cu3 O21.24
            Atom count from _chemical_formula_moiety:C18 H20.48 Cu3 O130.2400
ABSMU01_ALERT_1_G  Calculation of _exptl_absorpt_correction_mu
                not performed for this radiation type.
PLAT002_ALERT_2_G Number of Distance or Angle Restraints on AtSite         13 Note
PLAT003_ALERT_2_G Number of Uiso or Uij Restrained non-H Atoms ...          4 Report
PLAT004_ALERT_5_G Polymeric Structure Found with Maximum Dimension          3 Info
PLAT007_ALERT_5_G Number of Unrefined Donor-H Atoms .............           6 Report
PLAT013_ALERT_1_G N.O.K.   _shelx_hkl_checksum Found in CIF ......     Please Check
PLAT019_ALERT_1_G _diffrn_measured_fraction_theta_full/*_max < 1.0      0.994 Report
PLAT042_ALERT_1_G Calc. and Reported Moiety Formula Strings Differ     Please Check
PLAT045_ALERT_1_G Calculated and Reported Z Differ by a Factor ...       3.00 Check
PLAT068_ALERT_1_G Reported F000 Differs from Calcd (or Missing)...     Please Check
PLAT092_ALERT_4_G Check: Wavelength Given is not Cu,Ga,Mo,Ag,In Ka    0.63000 Ang.
PLAT172_ALERT_4_G The CIF-Embedded .res File Contains DFIX Records          5 Report
PLAT173_ALERT_4_G The CIF-Embedded .res File Contains DANG Records          4 Report
PLAT177_ALERT_4_G The CIF-Embedded .res File Contains DELU Records          1 Report
PLAT178_ALERT_4_G The CIF-Embedded .res File Contains SIMU Records          1 Report
PLAT186_ALERT_4_G The CIF-Embedded .res File Contains ISOR Records          4 Report
PLAT300_ALERT_4_G Atom Site Occupancy of O2W        Constrained at       0.28 Check
PLAT300_ALERT_4_G Atom Site Occupancy of H1O2       Constrained at       0.28 Check
PLAT300_ALERT_4_G Atom Site Occupancy of H2O2       Constrained at       0.28 Check
PLAT300_ALERT_4_G Atom Site Occupancy of O3W        Constrained at       0.04 Check
PLAT300_ALERT_4_G Atom Site Occupancy of H1O3       Constrained at       0.04 Check
PLAT300_ALERT_4_G Atom Site Occupancy of H2O3       Constrained at       0.04 Check
PLAT300_ALERT_4_G Atom Site Occupancy of O4W        Constrained at        0.2 Check
PLAT300_ALERT_4_G Atom Site Occupancy of H1O4       Constrained at        0.2 Check
PLAT300_ALERT_4_G Atom Site Occupancy of H2O4       Constrained at        0.2 Check
PLAT302_ALERT_4_G Anion/Solvent/Minor-Residue Disorder (Resd  2  )       100% Note
PLAT302_ALERT_4_G Anion/Solvent/Minor-Residue Disorder (Resd  3  )       100% Note
PLAT302_ALERT_4_G Anion/Solvent/Minor-Residue Disorder (Resd  4  )       100% Note
PLAT720_ALERT_4_G Number of Unusual/Non-Standard Labels ..........          7 Note
PLAT764_ALERT_4_G Overcomplete CIF Bond List Detected (Rep/Expd) .       1.11 Ratio
PLAT789_ALERT_4_G Atoms with Negative _atom_site_disorder_group  #          9 Check
PLAT794_ALERT_5_G Tentative Bond Valency for Cu1       (II)      .       2.19 Info
PLAT860_ALERT_3_G Number of Least-Squares Restraints ............          37 Note
PLAT883_ALERT_1_G No Info/Value for _atom_sites_solution_primary .     Please Do !
PLAT933_ALERT_2_G Number of OMIT Records in Embedded .res File ...         13 Note
```

   3 **ALERT level A** = Most likely a serious problem - resolve or explain
   2 **ALERT level B** = A potentially serious problem, consider carefully
   8 **ALERT level C** = Check. Ensure it is not caused by an omission or oversight
  36 **ALERT level G** = General information/check it is not something unexpected

```
   8 ALERT type 1 CIF construction/syntax error, inconsistent or missing data
  11 ALERT type 2 Indicator that the structure model may be wrong or deficient
   6 ALERT type 3 Indicator that the structure quality may be low
  21 ALERT type 4 Improvement, methodology, query or suggestion
   3 ALERT type 5 Informative message, check
```

It is advisable to attempt to resolve as many as possible of the alerts in all categories. Often the minor alerts point to easily fixed oversights, errors and omissions in your CIF or refinement strategy, so attention to these fine details can be worthwhile. In order to resolve some of the more serious problems it may be necessary to carry out additional measurements or structure refinements. However, the purpose of your study may justify the reported deviations and the more serious of these should normally be commented upon in the discussion or experimental section of a paper or in the "special_details" fields of the CIF. checkCIF was carefully designed to identify outliers and unusual parameters, but every test has its limitations and alerts that are not important in a particular case may appear. Conversely, the absence of alerts does not guarantee there are no aspects of the results needing attention. It is up to the individual to critically assess their own results and, if necessary, seek expert advice.

Publication of your CIF in IUCr journals

A basic structural check has been run on your CIF. These basic checks will be run on all CIFs submitted for publication in IUCr journals (*Acta Crystallographica*, *Journal of Applied Crystallography*, *Journal of Synchrotron Radiation*); however, if you intend to submit to *Acta Crystallographica Section C* or *E* or *IUCrData*, you should make sure that full publication checks are run on the final version of your CIF prior to submission.

Publication of your CIF in other journals

Please refer to the *Notes for Authors* of the relevant journal for any special instructions relating to CIF submission.

PLATON version of 22/03/2021; check.def file version of 19/03/2021

Datablock H2O-HK8th - ellipsoid plot

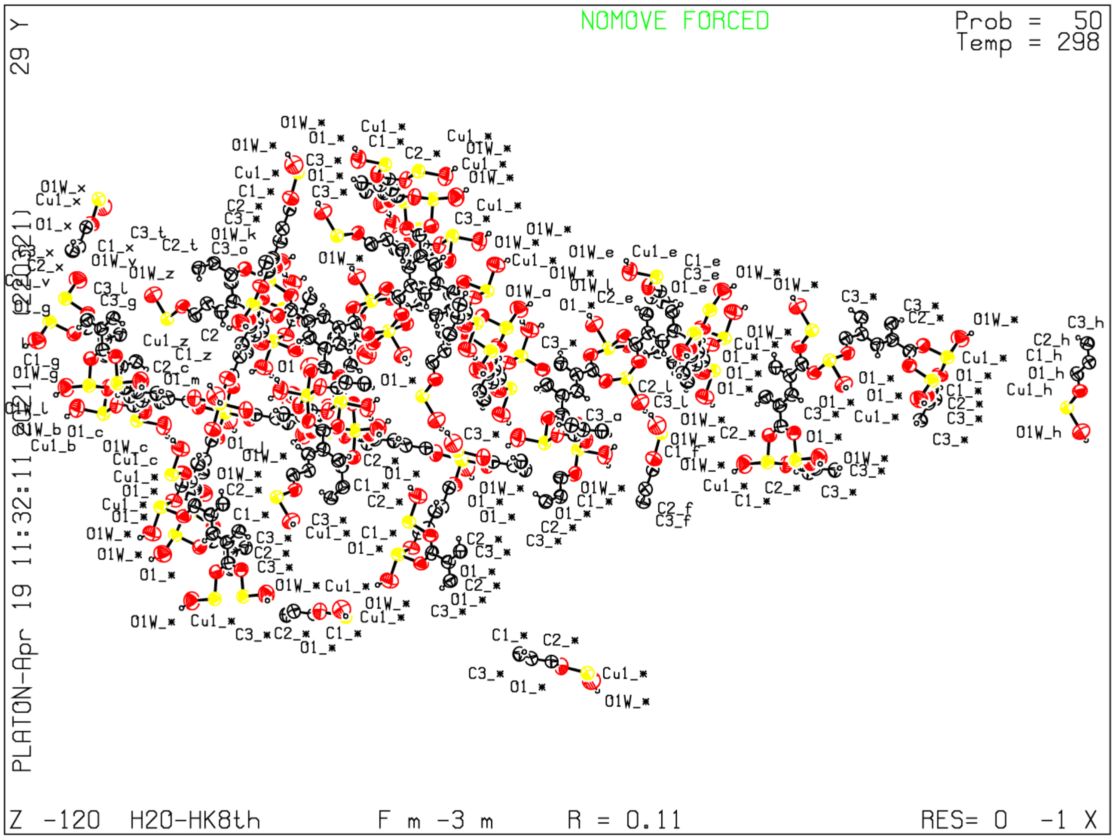

Supplement: Supplementary file 3 — Supplementary Data 1 [file 42004_2022_666_MOESM3_ESM.zip › 298_H2O-HK(8th).pdf]
