# Supplementary material for: Crystalline hydrogen bonding of water molecules confined in a metal-organic framework
Source: Commun Chem. 2022 Apr 8;5:51. doi: 10.1038/s42004-022-00666-8 (PMC9814150; doi:10.1038/s42004-022-00666-8)
Supplement: Supplementary file 3 — Supplementary Data 1 [file 42004_2022_666_MOESM3_ESM.zip › 298_H2O-HK(8th).rtf]

  Table 1.  Crystal data and structure refinement for H2O-HK(8th).
Identification code 	H2O-HK(8th)
Empirical formula 	C18 H24.48 Cu3 O21.24
Formula weight 	771.31
Temperature 	298(2) K
Wavelength 	0.630 Å
Crystal system 	Cubic
Space group 	Fm-3m
Unit cell dimensions	a = 26.393(3) Å	a= 90°.
	b = 26.393(3) Å	b= 90°.
	c = 26.393(3) Å	g = 90°.
Volume	18385(6) Å3
Z	16
Density (calculated)	1.115 Mg/m3
Absorption coefficient	1.030 mm-1
F(000)	6230
Crystal size	0.054 x 0.052 x 0.050 mm3
Theta range for data collection	1.185 to 25.989°.
Index ranges	-36<=h<=36, -36<=k<=36, -36<=l<=36
Reflections collected	46968
Independent reflections	1334 [R(int) = 0.3525]
Completeness to theta = 22.210°	98.4 % 
Absorption correction	Empirical
Max. and min. transmission	1.000 and 0.815
Refinement method	Full-matrix least-squares on F2
Data / restraints / parameters	1334 / 37 / 65
Goodness-of-fit on F2	1.035
Final R indices [I>2sigma(I)]	R1 = 0.1070, wR2 = 0.3189
R indices (all data)	R1 = 0.1989, wR2 = 0.3769
Extinction coefficient	n/a
Largest diff. peak and hole	0.599 and -0.419 e.Å-3

 Table 2.  Atomic coordinates  ( x 104) and equivalent  isotropic displacement parameters (Å2x 103)
for H2O-HK(8th).  U(eq) is defined as one third of  the trace of the orthogonalized Uij tensor.
________________________________________________________________________________ 
	x	y	z	U(eq)
________________________________________________________________________________  
Cu(1)	2149(1)	2851(1)	5000	94(1)
O(1)	2565(2)	3169(2)	5526(2)	112(2)
C(1)	2962(3)	2962(3)	5697(4)	96(3)
C(2)	3223(3)	3223(3)	6135(4)	100(3)
C(3)	3645(3)	3008(5)	6355(3)	103(3)
O(1W)	1585(4)	3415(4)	5000	155(5)
O(2W)	792(12)	2294(2)	4900(1)	193(11)
O(3W)	765(11)	809(7)	5822(14)	200(40)
O(4W)	713(7)	3177(13)	5663(2)	243(18)
________________________________________________________________________________ 
 Table 3.   Bond lengths [Å] and angles [°] for  H2O-HK(8th).
_____________________________________________________ 
Cu(1)-O(1)#1 	1.958(6)
Cu(1)-O(1)#2 	1.958(6)
Cu(1)-O(1)#3 	1.958(6)
Cu(1)-O(1) 	1.958(6)
Cu(1)-O(1W) 	2.105(14)
Cu(1)-Cu(1)#4 	2.619(4)
O(1)-C(1) 	1.265(7)
C(1)-C(2) 	1.5102(10)
C(2)-C(3)#5 	1.378(7)
C(2)-C(3) 	1.378(7)
C(3)-H(3) 	0.9300
O(1W)-H(1O1) 	0.919(7)
O(1W)-H(1O1)#1 	0.919(7)
O(2W)-H(1O2) 	0.9200(10)
O(2W)-H(2O2) 	0.9201(10)
O(3W)-H(1O3) 	0.9200(10)
O(3W)-H(2O3) 	0.9200(10)
O(4W)-H(1O4) 	0.9200(10)
O(4W)-H(2O4) 	0.9200(10)

O(1)#1-Cu(1)-O(1)#2	90.2(4)
O(1)#1-Cu(1)-O(1)#3	88.8(4)
O(1)#2-Cu(1)-O(1)#3	169.3(3)
O(1)#1-Cu(1)-O(1)	169.3(3)
O(1)#2-Cu(1)-O(1)	88.8(4)
O(1)#3-Cu(1)-O(1)	90.2(4)
O(1)#1-Cu(1)-O(1W)	95.33(17)
O(1)#2-Cu(1)-O(1W)	95.33(17)
O(1)#3-Cu(1)-O(1W)	95.33(17)
O(1)-Cu(1)-O(1W)	95.33(17)
O(1)#1-Cu(1)-Cu(1)#4	84.67(17)
O(1)#2-Cu(1)-Cu(1)#4	84.67(17)
O(1)#3-Cu(1)-Cu(1)#4	84.67(17)
O(1)-Cu(1)-Cu(1)#4	84.67(17)
O(1W)-Cu(1)-Cu(1)#4	180.00(10)
C(1)-O(1)-Cu(1)	122.1(5)
O(1)#6-C(1)-O(1)	126.0(9)
O(1)#6-C(1)-C(2)	117.0(4)
O(1)-C(1)-C(2)	117.0(4)
C(3)#5-C(2)-C(3)	119.0(11)
C(3)#5-C(2)-C(1)	120.3(6)
C(3)-C(2)-C(1)	120.3(6)
C(2)#7-C(3)-C(2)	121.0(11)
C(2)#7-C(3)-H(3)	119.5
C(2)-C(3)-H(3)	119.5
Cu(1)-O(1W)-H(1O1)	124.2(13)
Cu(1)-O(1W)-H(1O1)#1	124.2(13)
H(1O1)-O(1W)-H(1O1)#1	112(3)
H(1O2)-O(2W)-H(2O2)	115.20(14)
H(1O3)-O(3W)-H(2O3)	96.93(11)
H(1O4)-O(4W)-H(2O4)	96.96(10)
_____________________________________________________________ 
Symmetry transformations used to generate equivalent atoms: 
#1 -y+1/2,-x+1/2,-z+1    #2 -y+1/2,-x+1/2,z    #3 x,y,-z+1      
#4 -x+1/2,-y+1/2,-z+1    #5 y,-z+1,-x+1    #6 y,x,z      
#7 -z+1,x,-y+1      

 Table 4.   Anisotropic displacement parameters  (Å2x 103) for H2O-HK(8th).  The anisotropic
displacement factor exponent takes the form:  -2p2[ h2 a*2U11 + ...  + 2 h k a* b* U12 ]
______________________________________________________________________________ 
	U11	U22 	U33	U23	U13	U12
______________________________________________________________________________ 
Cu(1)	94(1) 	94(1)	93(1) 	0	0 	10(1)
O(1)	112(4) 	109(4)	114(4) 	-12(3)	-21(3) 	27(4)
C(1)	100(5) 	100(5)	88(7) 	2(4)	2(4) 	-15(7)
C(2)	105(5) 	105(5)	91(7) 	-2(4)	-2(4) 	5(7)
C(3)	101(5) 	108(8)	101(5) 	-5(5)	-8(7) 	5(5)
O(1W)	145(6) 	145(6)	174(11) 	0	0 	38(9)
O(2W)	159(15) 	227(17)	191(19) 	-42(17)	-24(17) 	-15(14)
O(3W)	200(40) 	200(40)	200(40) 	0(20)	0(20) 	0(20)
O(4W)	240(20) 	250(20)	240(20) 	14(19)	19(18) 	9(19)
______________________________________________________________________________ 
 Table 5.   Hydrogen coordinates ( x 104) and isotropic  displacement parameters (Å2x 10 3)
for H2O-HK(8th).
________________________________________________________________________________ 
	x 	y 	z 	U(eq)
________________________________________________________________________________ 
 
H(3)	3783	2715	6217	124
H(1O1)	1243(5)	3350(8)	5000	232
H(1O2)	750	1972	5027	289
H(2O2)	850	2541	5138	289
H(1O3)	1011	577	5907	297
H(2O3)	599	610	5588	297
H(1O4)	842	3168	5987	365
H(2O4)	414	3339	5741	365
________________________________________________________________________________ 
 Table 6.  Torsion angles [°] for H2O-HK(8th).
________________________________________________________________ 
Cu(1)-O(1)-C(1)-O(1)#6	7.8(17)
Cu(1)-O(1)-C(1)-C(2)	-174.6(7)
O(1)#6-C(1)-C(2)-C(3)#5	-177.3(10)
O(1)-C(1)-C(2)-C(3)#5	4.9(17)
O(1)#6-C(1)-C(2)-C(3)	-4.9(17)
O(1)-C(1)-C(2)-C(3)	177.3(10)
C(3)#5-C(2)-C(3)-C(2)#7	-3(3)
C(1)-C(2)-C(3)-C(2)#7	-175.3(8)
________________________________________________________________ 
Symmetry transformations used to generate equivalent atoms: 
#1 -y+1/2,-x+1/2,-z+1    #2 -y+1/2,-x+1/2,z    #3 x,y,-z+1      
#4 -x+1/2,-y+1/2,-z+1    #5 y,-z+1,-x+1    #6 y,x,z      
#7 -z+1,x,-y+1      

 Table 7.  Hydrogen bonds for H2O-HK(8th)  [Å and °].
____________________________________________________________________________ 
D-H...A	d(D-H)	d(H...A)	d(D...A)	<(DHA)
____________________________________________________________________________ 
 O(1W)-H(1O1)...O(4W^a)	0.919(7)	2.29(2)	2.96(2)	129.7(4)
 O(1W)-H(1O1)...O(4W^a)#3	0.919(7)	2.29(2)	2.96(2)	129.7(4)
 O(1W)-H(1O1)...O(4W^a)#8	0.919(7)	2.467(12)	3.139(10)	130.1(4)
 O(1W)-H(1O1)...O(4W^a)#9	0.919(7)	2.467(12)	3.139(10)	130.1(4)
 O(4W^a)-H(2O4^a)...O(1W)#10	0.9200(10)	2.489(8)	3.139(10)	127.9(8)
____________________________________________________________________________ 
Symmetry transformations used to generate equivalent atoms: 
#1 -y+1/2,-x+1/2,-z+1    #2 -y+1/2,-x+1/2,z    #3 x,y,-z+1      
#4 -x+1/2,-y+1/2,-z+1    #5 y,-z+1,-x+1    #6 y,x,z      
#7 -z+1,x,-y+1    #8 z-1/2,y,-x+1/2    #9 z-1/2,y,x+1/2      
#10 -z+1/2,-x+1/2,-y+1      

 
 
