# Supplementary material for: Crystalline hydrogen bonding of water molecules confined in a metal-organic framework
Source: Commun Chem. 2022 Apr 8;5:51. doi: 10.1038/s42004-022-00666-8 (PMC9814150; doi:10.1038/s42004-022-00666-8)

checkCIF/PLATON report

You have not supplied any structure factors. As a result the full set of tests cannot be run.

THIS REPORT IS FOR GUIDANCE ONLY. IF USED AS PART OF A REVIEW PROCEDURE FOR PUBLICATION, IT SHOULD NOT REPLACE THE EXPERTISE OF AN EXPERIENCED CRYSTALLOGRAPHIC REFEREE.

No syntax errors found.   CIF dictionary   Interpreting this report

Datablock: Act-HK

```
Bond precision:  C-C = 0.0050 A          Wavelength=0.63000

Cell:              a=26.249(3)      b=26.249(3)      c=26.249(3)
                   alpha=90         beta=90          gamma=90
Temperature:       298 K
```

	Calculated	Reported
Volume	18086(6)	18086(6)
Space group	F m -3 m	F m -3 m
Hall group	-F 4 2 3	-F 4 2 3
Moiety formula	C6 H2 Cu O4	C18 H6 Cu3 O12
Sum formula	C6 H2 Cu O4	C18 H6 Cu3 O12
Mr	201.63	604.85
Dx,g cm-3	0.889	0.889
Z	48	16
Mu (mm-1)	1.027	1.027
F000	4752.0	4752.0
F000’	4769.64	
h,k,lmax	36,36,36	36,36,36
Nref	1333	1323
Tmin,Tmax	0.946,0.950	0.824,1.000
Tmin’	0.946	

```
Correction method= # Reported T Limits: Tmin=0.824 Tmax=1.000
AbsCorr = EMPIRICAL

Data completeness= 0.992          Theta(max)= 25.975

R(reflections)= 0.0548( 793)      wR2(reflections)= 0.1707( 1323)

S = 1.031                 Npar= 32
```

```
The following ALERTS were generated. Each ALERT has the format
       test-name_ALERT_alert-type_alert-level.
Click on the hyperlinks for more details of the test.
```

Alert level A

```
PLAT601_ALERT_2_A Unit Cell Contains Solvent Accessible VOIDS of .      12465 Ang**3
```

Author Response: This crystal sample have fully dried under vacuum 16hour at 298K. It has observed that the unit cell contains large solvent accesible area in the crystal during the structure analysis. And no significant structure solvent coul be crystallophically located residing in the voids.

Alert level B

```
RINTA01_ALERT_3_B  The value of Rint is greater than 0.18
            Rint given   0.186
```

Author Response: The crystal was very small and weakly diffracting even after prolonged exposure time from challenging sample. However, the structure was determined and refined properly.

```
PLAT049_ALERT_1_B Calculated Density Less Than 1.0 gcm-3 .........     0.8886 Check
```

Author Response: Large voids account for the low density of the structure.

Alert level G

```
ABSMU01_ALERT_1_G  Calculation of _exptl_absorpt_correction_mu
                not performed for this radiation type.
PLAT004_ALERT_5_G Polymeric Structure Found with Maximum Dimension        3 Info
PLAT013_ALERT_1_G N.O.K.   _shelx_hkl_checksum Found in CIF ......     Please Check
PLAT019_ALERT_1_G _diffrn_measured_fraction_theta_full/*_max < 1.0      0.997 Report
PLAT020_ALERT_3_G The Value of Rint is Greater Than 0.12 .........      0.186 Report
PLAT042_ALERT_1_G Calc. and Reported Moiety Formula Strings Differ     Please Check
PLAT045_ALERT_1_G Calculated and Reported Z Differ by a Factor ...       3.00 Check
PLAT092_ALERT_4_G Check: Wavelength Given is not Cu,Ga,Mo,Ag,In Ka    0.63000 Ang.
PLAT764_ALERT_4_G Overcomplete CIF Bond List Detected (Rep/Expd) .       1.13 Ratio
PLAT802_ALERT_4_G CIF Input Record(s) with more than 80 Characters        1 Info
PLAT883_ALERT_1_G No Info/Value for _atom_sites_solution_primary .     Please Do !
PLAT933_ALERT_2_G Number of OMIT Records in Embedded .res File ...        7 Note
```

```
   1 ALERT level A = Most likely a serious problem - resolve or explain
   2 ALERT level B = A potentially serious problem, consider carefully
   0 ALERT level C = Check. Ensure it is not caused by an omission or oversight
  12 ALERT level G = General information/check it is not something unexpected

   7 ALERT type 1 CIF construction/syntax error, inconsistent or missing data
   2 ALERT type 2 Indicator that the structure model may be wrong or deficient
   2 ALERT type 3 Indicator that the structure quality may be low
   3 ALERT type 4 Improvement, methodology, query or suggestion
   1 ALERT type 5 Informative message, check
```

It is advisable to attempt to resolve as many as possible of the alerts in all categories. Often the minor alerts point to easily fixed oversights, errors and omissions in your CIF or refinement strategy, so attention to these fine details can be worthwhile. In order to resolve some of the more serious problems it may be necessary to carry out additional measurements or structure refinements. However, the purpose of your study may justify the reported deviations and the more serious of these should normally be commented upon in the discussion or experimental section of a paper or in the "special_details" fields of the CIF. checkCIF was carefully designed to identify outliers and unusual parameters, but every test has its limitations and alerts that are not important in a particular case may appear. Conversely, the absence of alerts does not guarantee there are no aspects of the results needing attention. It is up to the individual to critically assess their own results and, if necessary, seek expert advice.

Publication of your CIF in IUCr journals

A basic structural check has been run on your CIF. These basic checks will be run on all CIFs submitted for publication in IUCr journals (*Acta Crystallographica*, *Journal of Applied Crystallography*, *Journal of Synchrotron Radiation*); however, if you intend to submit to *Acta Crystallographica Section C* or *E* or *IUCrData*, you should make sure that full publication checks are run on the final version of your CIF prior to submission.

Publication of your CIF in other journals

Please refer to the *Notes for Authors* of the relevant journal for any special instructions relating to CIF submission.

PLATON version of 22/03/2021; check.def file version of 19/03/2021

Datablock Act-HK - ellipsoid plot

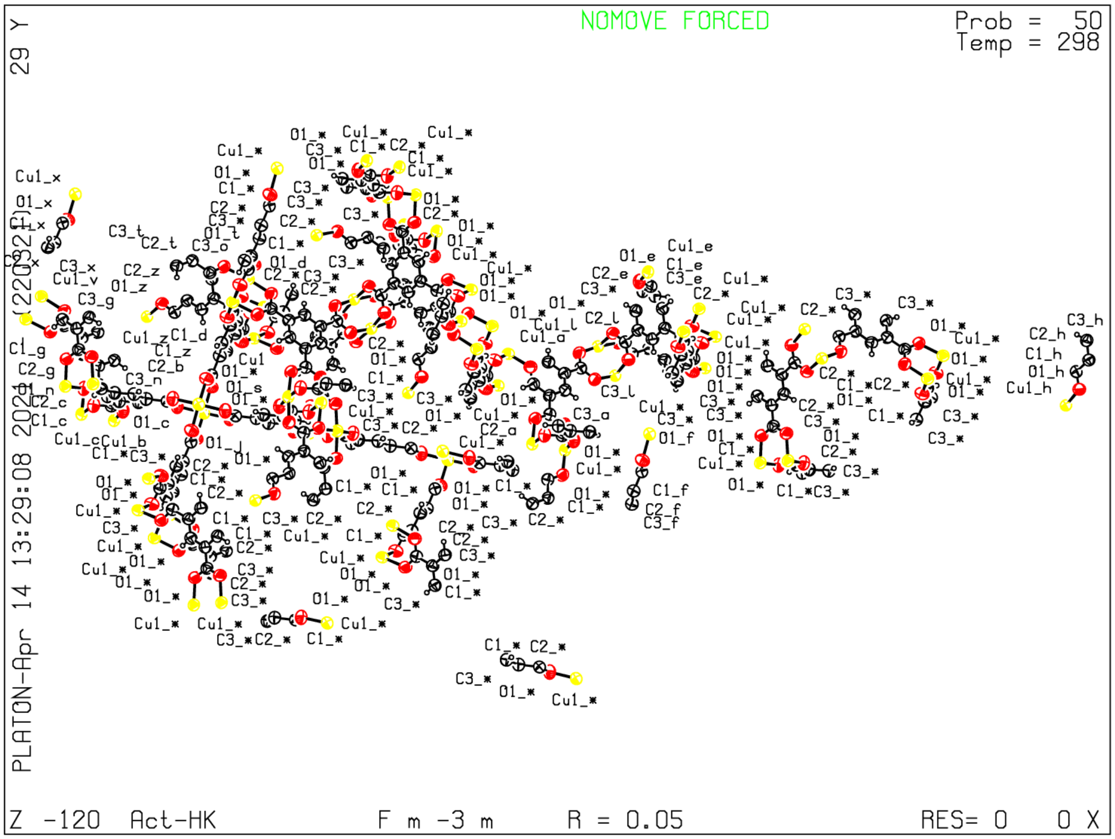

Supplement: Supplementary file 3 — Supplementary Data 1 [file 298_Act-HK.pdf › 298_Act-HK.pdf]
