# Supplementary material for: Crystalline hydrogen bonding of water molecules confined in a metal-organic framework
Source: Commun Chem. 2022 Apr 8;5:51. doi: 10.1038/s42004-022-00666-8 (PMC9814150; doi:10.1038/s42004-022-00666-8)

checkCIF/PLATON report

You have not supplied any structure factors. As a result the full set of tests cannot be run.

THIS REPORT IS FOR GUIDANCE ONLY. IF USED AS PART OF A REVIEW PROCEDURE FOR PUBLICATION, IT SHOULD NOT REPLACE THE EXPERTISE OF AN EXPERIENCED CRYSTALLOGRAPHIC REFEREE.

No syntax errors found. CIF dictionary Interpreting this report

Datablock: Act-HK

```
Bond precision:  C-C = 0.0016 A          Wavelength=0.61000

Cell:              a=26.267(3)      b=26.267(3)      c=26.267(3)
                   alpha=90         beta=90          gamma=90
Temperature:       220 K

                Calculated                 Reported
Volume          18123(6)                   18123(6)
Space group     F m -3 m                   F m -3 m
Hall group      -F 4 2 3                   -F 4 2 3
Moiety formula  C6 H2 Cu O4                C18 H6 Cu3 O12
Sum formula     C6 H2 Cu O4                C18 H6 Cu3 O12
Mr              201.63                     604.85
Dx,g cm-3       0.887                      0.887
Z               48                         16
Mu (mm-1)       0.938                      0.938
F000            4752.0                     4752.0
F000’           4769.37
h,k,lmax        36,36,36                   36,36,36
Nref            1321                       1320
Tmin,Tmax       0.889,0.923                0.956,1.000
Tmin’           0.889

Correction method= # Reported T Limits: Tmin=0.956 Tmax=1.000
AbsCorr = EMPIRICAL

Data completeness= 0.999          Theta(max)= 24.993

R(reflections)= 0.0273( 1303)     wR2(reflections)= 0.0731( 1320)

S = 1.073                  Npar= 33
```

```
The following ALERTS were generated. Each ALERT has the format
       test-name_ALERT_alert-type_alert-level.
Click on the hyperlinks for more details of the test.
```

Alert level A

```
PLAT601_ALERT_2_A Unit Cell Contains Solvent Accessible VOIDS of .      12485 Ang**3
```

Author Response: This crystal sample have fully dried under vacuum in the glove box. It has observed that the unit cell contains large solvent accesible area in the crystal during the structure analysis. And no significant structure solvent coul be crystallophically located residing in the voids.

Alert level B

```
PLAT049_ALERT_1_B Calculated Density Less Than 1.0 gcm-3 .........     0.8868 Check
```

Author Response: Large voids account for the low density of the structure.

Alert level G

```
ABSMU01_ALERT_1_G  Calculation of _exptl_absorpt_correction_mu
                not performed for this radiation type.
PLAT004_ALERT_5_G Polymeric Structure Found with Maximum Dimension          2 Info
PLAT013_ALERT_1_G N.O.K.   _shelx_hkl_checksum Found in CIF ......     Please Check
PLAT042_ALERT_1_G Calc. and Reported Moiety Formula Strings Differ     Please Check
PLAT045_ALERT_1_G Calculated and Reported Z Differ by a Factor ...       3.00 Check
PLAT083_ALERT_2_G SHELXL Second Parameter in WGHT  Unusually Large      19.04 Why ?
PLAT092_ALERT_4_G Check: Wavelength Given is not Cu,Ga,Mo,Ag,In Ka    0.61000 Ang.
PLAT764_ALERT_4_G Overcomplete CIF Bond List Detected (Rep/Expd) .       1.13 Ratio
PLAT802_ALERT_4_G CIF Input Record(s) with more than 80 Characters          1 Info
PLAT883_ALERT_1_G No Info/Value for _atom_sites_solution_primary .     Please Do !
```

```
   1 ALERT level A = Most likely a serious problem - resolve or explain
   1 ALERT level B = A potentially serious problem, consider carefully
   0 ALERT level C = Check. Ensure it is not caused by an omission or oversight
  10 ALERT level G = General information/check it is not something unexpected

   6 ALERT type 1 CIF construction/syntax error, inconsistent or missing data
   2 ALERT type 2 Indicator that the structure model may be wrong or deficient
   0 ALERT type 3 Indicator that the structure quality may be low
   3 ALERT type 4 Improvement, methodology, query or suggestion
   1 ALERT type 5 Informative message, check
```

It is advisable to attempt to resolve as many as possible of the alerts in all categories. Often the minor alerts point to easily fixed oversights, errors and omissions in your CIF or refinement strategy, so attention to these fine details can be worthwhile. In order to resolve some of the more serious problems it may be necessary to carry out additional measurements or structure refinements. However, the purpose of your study may justify the reported deviations and the more serious of these should normally be commented upon in the discussion or experimental section of a paper or in the "special_details" fields of the CIF. checkCIF was carefully designed to identify outliers and unusual parameters, but every test has its limitations and alerts that are not important in a particular case may appear. Conversely, the absence of alerts does not guarantee there are no aspects of the results needing attention. It is up to the individual to critically assess their own results and, if necessary, seek expert advice.

Publication of your CIF in IUCr journals

A basic structural check has been run on your CIF. These basic checks will be run on all CIFs submitted for publication in IUCr journals (*Acta Crystallographica*, *Journal of Applied Crystallography*, *Journal of Synchrotron Radiation*); however, if you intend to submit to *Acta Crystallographica Section C* or *E* or *IUCrData*, you should make sure that full publication checks are run on the final version of your CIF prior to submission.

Publication of your CIF in other journals

Please refer to the *Notes for Authors* of the relevant journal for any special instructions relating to CIF submission.

PLATON version of 03/06/2021; check.def file version of 02/06/2021

Datablock Act-HK - ellipsoid plot

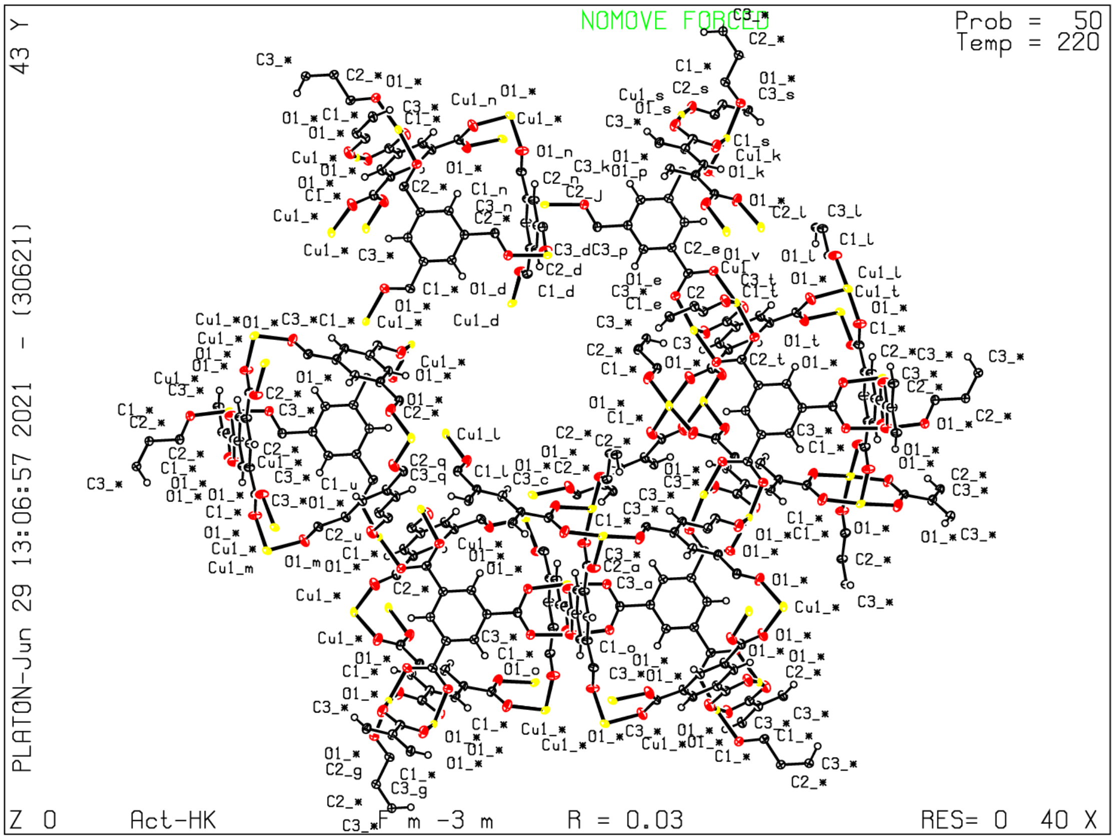

Supplement: Supplementary file 3 — Supplementary Data 1 [file 220_Act-HK.pdf › 220_Act-HK.pdf]
